# Beyond the Pain: A Critical Examination of the Psychopathological and Neuropsychological Dimensions of Primary Headaches in Pediatric Populations

**DOI:** 10.3390/life15101641

**Published:** 2025-10-21

**Authors:** Giuseppe Accogli, Valentina Nicolardi, Camilla Ferrante, Giorgia Carlucci, Sara Scoditti, Antonio Trabacca

**Affiliations:** 1 Scientific Institute IRCCS “E. Medea”, Scientific Direction, 23842 Bosisio Parini, Italy; giuseppe.accogli@lanostrafamiglia.it (G.A.); valentina.nicolardi@lanostrafamiglia.it (V.N.); 2 Associazione “La Nostra Famiglia”—IRCCS “E. Medea”—Scientific Hospital for Neurorehabilitation—Unit for Severe Disabilities in Developmental Age and Young Adults (Developmental Neurology and Neurorehabilitation), 72100 Brindisi, Italy; camilla.ferrante@lanostrafamiglia.it (C.F.); giorgia.carlucci@lanostrafamiglia.it (G.C.); sara.scoditti@lanostrafamiglia.it (S.S.)

**Keywords:** primary headaches, pediatric, psychopathology, neurocognitive functioning, quality of life

## Abstract

**Background:** Primary headaches in children and adolescents impose emotional, cognitive, and functional burdens beyond pain. This review synthesizes their psychopathological and neuropsychological dimensions and outlines implications for assessment and care. **Methods:** We performed a comprehensive review with systematic searches of PubMed, Scopus, and Embase (2015–2025). We included observational/experimental studies and evidence syntheses on 0–18-year-olds with migraine, tension-type, or cluster headache; treatment-only reports were excluded. **Results:** Across population and clinic samples, primary headaches co-occur with elevated anxiety/depression, frequent ADHD/learning problems, and pervasive sleep disturbances with likely bidirectionality. Interictally, small to moderate neurocognitive differences are most consistent in attention/executive control, processing speed, and verbal memory. Quality of life and school participation are reduced. Standardized tools (e.g., PedMIDAS, PedsQL/KIDSCREEN, SDQ/CBCL, SDSC±actigraphy, NEPSY-II/BRIEF) support multidisciplinary assessment. **Conclusions:** Care should look beyond pain counts, integrating routine screening of mood, sleep, and cognition; active family involvement; and school–healthcare coordination within stepped-care pathways (education and sleep hygiene for all; targeted CBT for catastrophizing/avoidance) with monitoring that pairs headache frequency with functional outcomes.

## 1. Introduction

Headache disorders are among the most common neurological conditions worldwide and are highly disabling, disrupting daily activities, school or work performance, and family and social functioning. Beyond pain, recurrent attacks and anticipatory worry add a substantial emotional burden, with frequent co-occurrence of anxiety and depression. Headache disorders rank among the top causes of years lived with disability (YLDs) [1,2]. These features are relevant across the lifespan and set the stage for age-specific considerations, addressed in the next section.

The International Classification of Headache Disorders, 3rd edition (ICHD-3), distinguishes primary headaches—occurring independently—from secondary headaches due to other medical conditions [3,4]. Primary headaches include migraine, tension-type headache (TTH), trigeminal autonomic cephalalgias (TACs), and rarer subtypes such as exertional, cold-stimulus, and epicranial headaches (e.g., stabbing, nummular) [5]. ICHD-3 offers operational diagnostic criteria based on intensity, duration, frequency, and associated symptoms, which promote consistency across settings and clarify when headache features warrant evaluation for secondary causes [6].

Among primary headaches, migraine and TTH are the most prevalent. Migraine typically involves moderate to severe, often unilateral, pulsating pain accompanied by nausea, photophobia/phonophobia, and movement intolerance; about one-third of patients experience transient aura symptoms, most commonly visual. Current pathophysiological models emphasize altered sensory processing within the trigeminovascular system, with roles for CGRP and genetic susceptibility. Interictal fatigue, mood changes, and cognitive complaints have also been reported [4,7]. TTH usually presents as bilateral, pressing/tightening pain of mild to moderate intensity, typically without nausea or vomiting; both episodic and chronic forms are recognized. Mechanisms are multifactorial, combining peripheral muscle-related factors with central sensitization and reduced descending inhibition, especially in chronic presentations [4,8,9]. TACs, though less common, are distinctive for strictly unilateral orbital/temporal pain with cranial autonomic symptoms (e.g., tearing, rhinorrhea); subtypes differ by attack duration, frequency, and treatment response, with hypothalamic involvement implicated [4]. Across primary headaches, psychiatric comorbidity is frequent and likely bidirectional; in migraine, depression is linked to a higher risk of chronification [2,8].

Building on this framework, the following section focuses on pediatric presentations, epidemiology, and age-specific diagnostic considerations.

### 1.1. Pediatric Perspective

In childhood and adolescence, primary headaches are common and consequential: they are leading medical causes of school absenteeism and affect quality of life at home, during leisure, and at school [10]. Recurrent pediatric headaches are associated with increased risk of later physical and mental health problems, particularly anxiety and depression [10]. Prevalence rises with age, from roughly 6–38% in preschoolers to ~38–50% in school-age children and up to ~75% in adolescents, though estimates vary by method. TTH is often under-recognized because of overlap with migraine and developmental shifts in phenotype; sex differences are modest for TTH (≈1.2:1 female:male) compared to migraine (≈3:1). A non-trivial proportion of preschool headaches remains unclassified or falls under childhood periodic syndromes, reflecting the limits of early-age phenotyping [10].

Applying formal diagnostic criteria in real-world pediatric clinics remains challenging. In a large neurology cohort, only ~58% of children received a specific IHS/ICHD diagnosis, leaving ~42% unspecified despite clear functional impairment; migraine and TTH were similarly prevalent and associated with frequent episodes, missed school days, and emotional difficulties [11]. ICHD-3 acknowledges that some presentations cannot be confidently categorized (e.g., “Headache unspecified”), underscoring classification constraints when information is limited (ICHD-3). Comorbidity is common: sleep problems and anxiety recur across pediatric phenotypes (e.g., sleep ≈ 8% in TTH, ≈12% in migraine; anxiety ≈3% in TTH, ≈16% in migraine), and psychiatric diagnoses appear more frequent in migraine than in TTH or controls. Longitudinally, recurrent headaches in youth predict a higher risk of adult psychopathology [10].

Psychosocial adversity amplifies vulnerability. Adverse childhood experiences (ACEs)—including abuse, neglect, bereavement, parental separation/divorce, and family psychiatric history—are cumulatively associated with higher rates of recurrent pain (including headaches), greater frequency, and increased anxiety/mood disorders; exposure to more than two traumatic events is linked to roughly a two-fold increase in headache prevalence. Alexithymia has been suggested as a potential therapeutic target in youths with recurrent primary headache and somatoform features [10]. At the population level, the burden remains substantial: migraine and TTH together account for a large share of prevalence and global YLDs, with recent trends showing rising disability among females and a specific increase in adolescent migraine [12].

### 1.2. Comorbidity, Psychosocial Factors, and Neurocognitive Profile

Primary headache disorders rank among the top contributors to disability globally (GBD 2019) and are the leading cause in ages 15–49; migraine alone accounts for a sizable share of all-cause YLDs. Multimorbidity is common—neurological, metabolic, cardiovascular, sleep, and mental health conditions frequently co-occur—complicating diagnosis, treatment, and causal interpretation [13]. Psychiatric comorbidity is particularly prominent: pooled estimates indicate elevated rates of anxiety, depression, and PTSD, with bidirectional links in which affective symptoms may precede frequent headaches or arise in their wake [13].

In pediatric cohorts, overlap with affective symptoms is especially evident among youths with chronic daily headache in specialty settings. Risk markers for greater physical and psychological burden include female sex, higher attack frequency, longer headache history, medication overuse, and specific personality features; early-life health and behavioral difficulties; and family history—especially maternal headache—also relate to later headache, though effects are smaller in community samples [14]. Health-related quality of life (HRQoL) is consistently reduced across diagnoses; while migraine is highly disabling and economically costly, TTH also shows broad HRQoL impairments, even when associated with fewer missed days. Evidence for cluster headache in youth remains limited due to small samples [15].

Individual differences and transdiagnostic processes add explanatory value. Chronic presentations tend to co-occur with higher neurotic/anxious–depressive traits and greater pain catastrophizing, with the most maladaptive profiles in frequent–chronic and medication-overuse forms—clear targets for psychological intervention [16]. Fear of pain, stress, and anxiety sensitivity show medium associations with disability, consistent with fear-avoidance mechanisms that sustain functional impairment [17]. Family context matters: children with primary headaches report more negative affect and internalizing symptoms than controls, and maternal perceptions often mirror this burden; maternal emotion-regulation difficulties—particularly lower emotional awareness and goal-directed behavior—are associated with higher child headache frequency and adjustment problems, supporting family-level assessment [18]. Consistently, in young individuals with migraine, the presence of migraine equivalents is associated with greater anxiety and somatization [19]. Notably, in clinical studies, an ambivalent attachment style has been associated with greater frequency/severity of attacks and with more anxiety-depressive symptoms [20].

Neurodevelopmental comorbidity is salient. Migraine is associated with ADHD (less so TTH), and both ADHD and ASD can co-occur with recurrent headaches; proposed mechanisms include catecholaminergic and GABAergic pathways (ADHD) and sensory hyperreactivity (ASD). Clinicians should also consider that ADHD medications (e.g., methylphenidate, atomoxetine, guanfacine) may precipitate headaches in some cases [21]. Cognitively, meta-analytic evidence indicates small to moderate interictal weaknesses in young people with primary headaches—most consistently in migraine—spanning motor and executive functions, learning/memory, language, processing speed, intelligence, and visuospatial skills. Reviews recommend routine cognitive screening in pediatric headache (especially migraine) given recurrent observations of attention, processing speed, and memory difficulties and possible higher rates of learning problems versus TTH; adult findings converge on executive function, processing speed, and memory deficits, with limited evidence for dementia risk and mixed results for TTH. Despite heterogeneous methods, several studies highlight perceptual and executive vulnerabilities in migraine and call for phase-specific and longitudinal designs [22,23,24,25].

In this comprehensive review, we provide a critical synthesis of the evidence on the psychopathological and neuropsychological profiles of children and adolescents with primary headaches. We aim to offer a broad overview of the association between primary headaches and youth’s emotional–behavioral difficulties, neurocognitive functioning, and overall psychosocial adjustment, including quality of life and school participation. Furthermore, we map the various assessment instruments used in the field to support comparability across studies and facilitate clinical translation.

This work is structured as a comprehensive narrative review, integrating findings from empirical and review studies published over the past decade to provide an updated overview of current knowledge in this field.

## 2. Methods

### 2.1. Design and Scope

We conducted a comprehensive, narrative review with a systematic search strategy to map the psychological and neuropsychological correlates of primary headaches in pediatric populations. Eligible evidence included peer-reviewed systematic reviews, meta-analyses, and narrative reviews, as well as observational or descriptive studies; reports focused exclusively on clinical or pharmacological treatments were not considered within scope.

### 2.2. Eligibility Criteria

Inclusion criteria

- Population: Children and adolescents aged 0–18 years. Mixed samples (e.g., pediatric+adult) were eligible only when pediatric-specific data or analyses were reported.
- Condition: Primary headache disorders—migraine (and subtypes), tension-type headache, and cluster headache—classified according to ICHD-3 and national guideline terminology (SISC).
- Outcomes: At least one psychopathological outcome (e.g., anxiety, depression, ADHD, conduct problems) and/or neuropsychological functioning (e.g., attention, memory, executive functions), including emotional/behavioral profiles.
- Publication characteristics: Articles in English, published 2015–2025, with full text available.
- No geographical restrictions were applied; studies from any country were eligible if all other inclusion criteria were met.

Exclusion criteria

- Studies not clearly distinguishing primary from secondary headaches, or where headache was only a marginal manifestation of broader genetic/systemic conditions.
- Publications not reporting data relevant to either psychopathology or neuropsychology.
- Editorials, opinion pieces, unstructured single-case reports, and records without retrievable full text.
- Adult-only samples or mixed samples without extractable pediatric data.

### 2.3. Information Sources and Search Strategy

Searches were run in Scopus, Embase, and PubMed. Boolean strings combined three concept blocks—condition, population, and outcomes—and were adapted to each database. Examples include the following:

- Scopus: (Title/Abstract/Keywords): (“primary headache” OR migraine OR “tension-type headache” OR “cluster headache”) AND (child* OR adolescen* OR pediatric OR paediatric OR youth) AND (psychopathology* OR neuropsychology* OR “cognitive function*” OR “executive function*” OR attention OR memory OR “behavioral problem*” OR “emotional problem*” OR “mental health”).
- Embase: (ti,ab,kw): analogous terms with year (2015–2025) and English-language limits applied.
- PubMed: MeSH and title/abstract terms for primary headache disorders, pediatric populations, and psychopathology/neuropsychology constructs; English and 2015–2025 limits were applied.

The research was concluded on 11 July 2025. The complete database search strings and filters are provided in Table 1.

### 2.4. Study Selection

**Figure 1:**
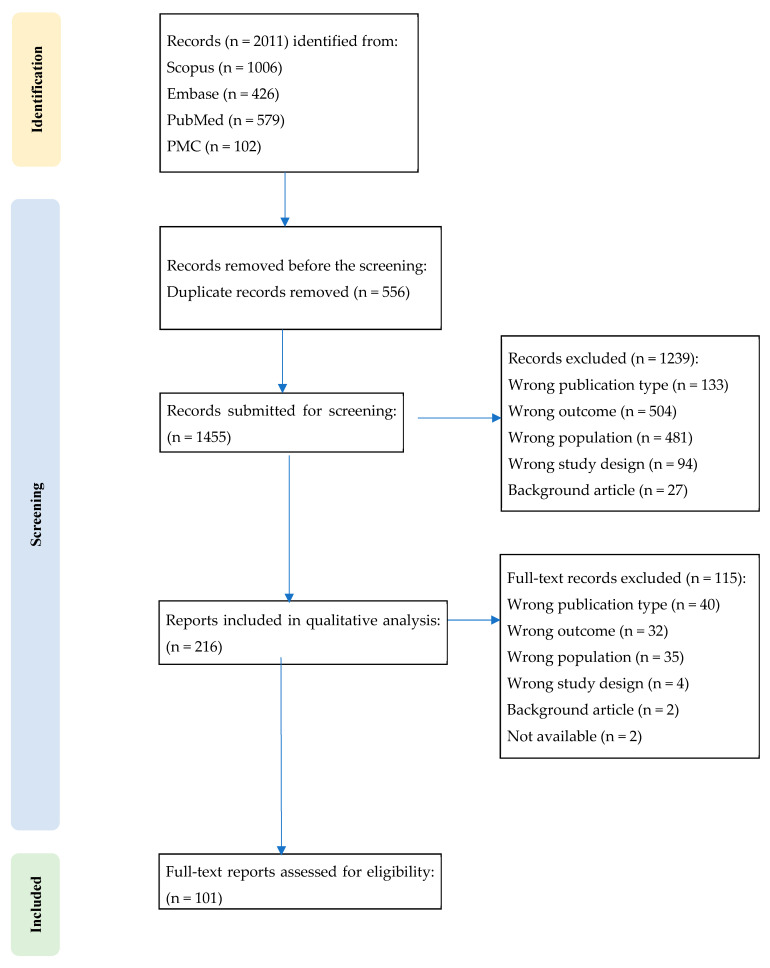
PRISMA flow diagram.

All records were imported, and duplicates were removed prior to screening. Title/abstract screening was followed by full-text assessment against eligibility criteria. Reasons for exclusion were recorded (e.g., wrong publication type, outcome, population, or design). Two researchers independently conducted the literature search, and any conflicts between the two researchers were resolved with the intervention of a third party. A PRISMA flow diagram was prepared (Figure 1), in line with PRISMA guidelines [26], to enhance transparency and replicability in the reporting of the selection process. Given the comprehensive and narrative nature of the review, no formal risk-of-bias assessment was performed, as it is not mandatory in this type of review [27].

### 2.5. Data Charting and Synthesis

From each included article, we charted key study descriptors (publication type, design), sample characteristics, outcome domains, assessment instruments (psychopathological and neuropsychological), and conceptual focus. Given heterogeneity in designs and outcomes, the findings were synthesized narratively, grouping evidence by headache subtype and by outcome domain (psychopathology vs. neuropsychology), with attention to developmental considerations. No quantitative meta-analysis was planned. We predefined two evidence streams and synthesized them separately: (i) primary empirical studies (experimental and observational) and (ii) secondary evidence (narrative/systematic reviews and meta-analyses). Data extraction and quality appraisal were performed by stream, and the results are reported in distinct subsections to avoid double counting and to preserve interpretability across study designs. Study characteristics were tabulated and selection steps visualized using a PRISMA flow diagram (Figure 1). The characteristics of the primary empirical studies are summarized in Table S1 (parts 1–2), organized by aim, experimental sample(s), control group, psychopathological and cognitive variables analyzed, assessment instruments, study design, main results, and limitations. The characteristics of the reviews and meta-analyses are summarized in Table S2 (parts 1–2), which charts for each work the main purpose of the review, research question(s), methods, number and type of studies included, main synthesized results/emerging themes, general conclusions, identified limitations of the literature, and future research directions. Given heterogeneity in populations, outcomes, and methods, we applied a narrative, theme-based grouping for synthesis rather than statistical pooling.

Given their length and level of detail, the tables are provided in the Supplementary Materials as Table S1 (parts 1–2) and Table S2 (parts 1–2), while the main text summarizes only the key findings.

### 2.6. Flow of Analysis and Synthesis

**Figure 2:**
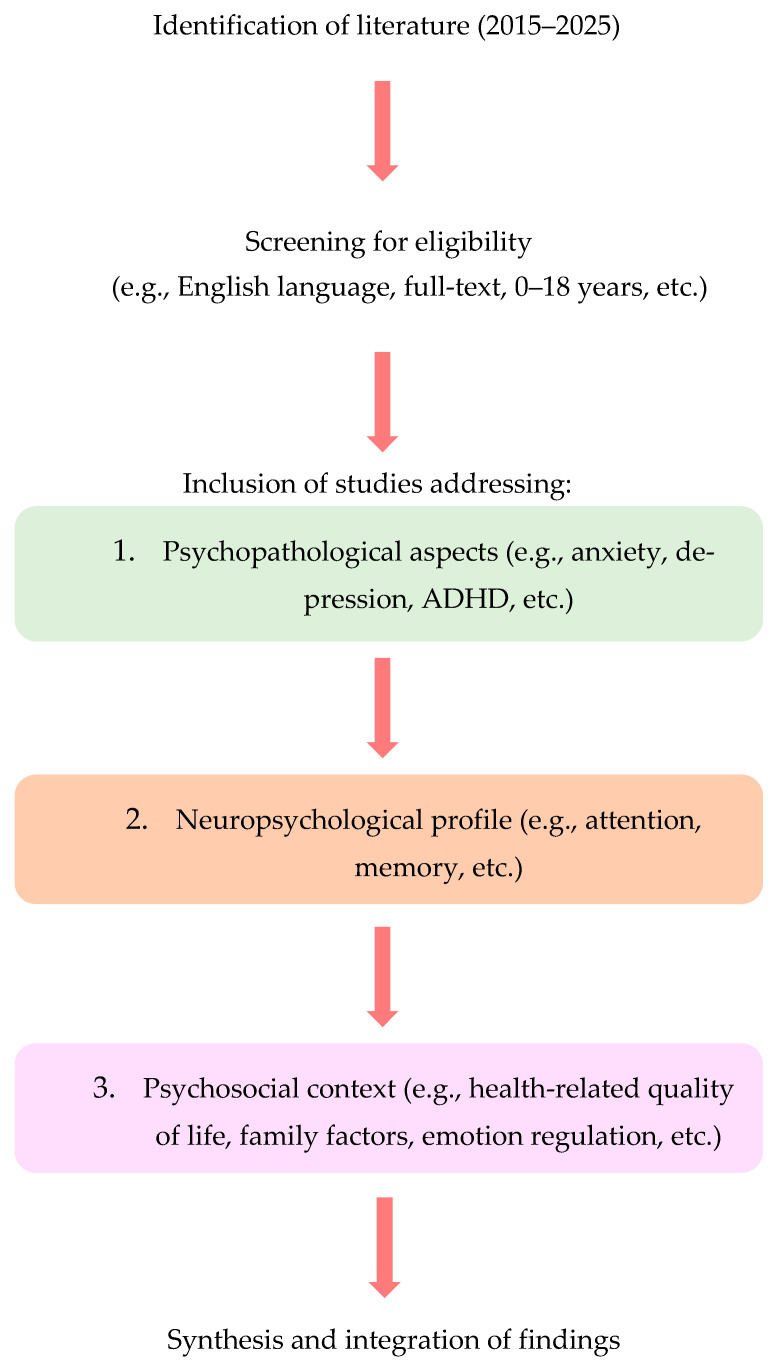
Flow chart summarizing the review process and thematic domains.

To provide a visual overview of the analytic process, a flow chart was developed (Figure 2). The diagram outlines the main steps of the review, from literature identification and eligibility screening to the synthesis of evidence across three thematic domains: psychopathological, neuropsychological, and psychosocial contexts. This visualization complements the narrative synthesis described above and clarifies how the studies were grouped and analyzed within the review.

## 3. Results

A total of 2011 records were identified through the initial database search. Following the removal of 556 duplicates, 1455 records were retained for screening. Based on titles and abstracts, 1239 records were excluded as not meeting the inclusion criteria. A total of 216 full-text articles were subsequently assessed for eligibility. Of these, 101 studies fulfilled the inclusion criteria and were incorporated into the qualitative content analysis.

Across population- and clinic-based studies, primary headaches in youth were consistently linked to elevated emotional–behavioral difficulties, selective neurocognitive differences, and impaired psychosocial adjustment (quality of life and school participation), with clinical and psychosocial features modulating severity.

We report findings in two parts: first the primary studies (experimental/observational), and then a separate synthesis of reviews and meta-analyses.

### 3.1. Results from Primary Studies (Experimental/Observational Evidence)

#### 3.1.1. Emotional–Behavioral Difficulties and Psychiatric Comorbidity

In a nationwide Brazilian school survey, adolescents with migraine—more than tension-type headache (TTH)—showed worse global psychosocial adjustment on the Strengths and Difficulties Questionnaire (SDQ), with symptom burden scaling with core migraine features [28]. In Canadian surveillance, adolescents with migraine had markedly higher odds of mood and anxiety disorders than peers [29]; statewide/insurance datasets echoed this excess mental health comorbidity among youths with migraine [30,31].

A large adolescent survey using WHO-CIDI with ICHD-based algorithms likewise linked migraine to a higher overall psychiatric burden, reinforcing the population-level association [32].

In community cohorts of children with long-term conditions (including migraine/severe headache), mental health remained poorer and school absence higher both cross-sectionally and at 3-year follow-up [33]. Within pediatric psychiatry settings, headache was common and tied to substantial disability [34], and children with anxiety disorders had significantly more migraine-like headaches than controls [35]. Clinic cohorts converge on high comorbidity: 82% of adolescents with primary headaches met criteria for a DSM diagnosis—most commonly anxiety—with profiles varying by headache features [36]; more than half of those with chronic migraine had at least one DSM-5 disorder, and attention problems tracked headache severity and disability [37]. A narrative update similarly estimated roughly a fourfold increase in internalizing disorders in primary headaches versus controls [38]. Longitudinal signals were mixed: depressive—but not anxiety—symptoms predicted greater disability over 5 years [39], whereas in a prospective clinic cohort depression was the only psychological factor strongly correlated with higher headache-related disability (r = 0.52); depression correlated positively with stress, anxiety, and sleep disturbance, and negatively with mindfulness, self-compassion, and resilience, while inflammatory markers and vagal tone (HRV) showed no association [40].

Population and outpatient data further show higher internalizing symptoms in youth with headache: adolescents with headache had increased anxiety/depression at follow-up in the Young-HUNT cohort [41]; in a Korean neurology clinic, brief screens identified a subgroup with clinically significant depressive/anxiety symptoms [42]. Consistently, in preschoolers, children with primary headache exhibited significantly more internalizing problems, whereas children with incontinence showed more externalizing problems (especially conduct problems). Primary headache was a significant predictor of internalizing symptoms, while constipation and fecal incontinence predicted externalizing symptoms [43].

In an Iranian case–control study, adolescents with migraine reported significantly higher anxiety and depression than controls, with more severe anxiety linked to more frequent/longer attacks [44].

A clinic sample from Turkey found elevated psychopathology and lower QoL in pediatric migraine patients vs. controls [45].

In a large German registry-based study, pediatric migraine was associated with the development of additional disorders during transition [46].

In clinic-based comparisons, children with primary headaches had higher CBCL/SAFA scores than healthy peers, with no significant psychopathology differences between migraine and other headache subtypes; mother–child internalizing reports were concordant, and familial recurrence was more prominent along the paternal line [47].

In a low-resource boarding-school cohort, migraine—relative to TTH and no headache—was associated with higher SDQ emotional problems and total difficulties, alongside greater pain (VAS) and disability (PedMIDAS) [48].

#### 3.1.2. Neurodevelopmental Conditions and Learning

ADHD co-occurs non-trivially with pediatric migraine across population-based and administrative datasets: in a large Brazilian school survey, ADHD symptoms were comorbid with migraine but not with tension-type headache, and risk was higher in chronic migraine [49]; in a nationwide cohort, individuals with ADHD had nearly double the subsequent hazard of receiving a migraine diagnosis [50]. In real-world neurology clinics using ICHD-3, ADHD was frequent across diagnoses and highest in adolescents with migraine; compared with TTH, migraine clustered with nausea/vomiting, photophobia/phonophobia, moderate to severe pain, activity impairment, and greater analgesic use, while emotional problems were more common in TTH [11]. Mixed headache (migraine+TTH) further carried elevated odds of formally diagnosed learning disabilities relative to migraine alone [51].

Clinically, ADHD also carried prognostic weight: among inpatients with status migrainosus, non-response to intensive protocols was predicted by ADHD and severe anxiety [52]. In a school sample, children with migraine scored higher on an ADHD screening measure (SNAP-IV), although the proportion with a clinically diagnosed ADHD did not differ from controls; moreover, a significantly larger share of migraineurs reported >1 h/day of screen time, whereas this association did not appear when stratifying by clinically diagnosed ADHD [53].

Headache co-occurring with a diagnosed learning disability was linked to higher anxiety, and in chronic TTH specifically, LD was associated with substantially greater absenteeism [54].

#### 3.1.3. Sleep Alterations and Clinical Impact

Sleep problems were pervasive and clinically meaningful. Parent-rated sleep difficulties co-varied with internalizing symptoms in pediatric primary headaches [55]. Polysomnography documented altered sleep architecture in pediatric migraine, with a higher percentage of N2 sleep and a lower percentage of slow-wave (N3) sleep in clinic-referred cohorts [56]; in a matched case–control study, migraineurs had 5.6-fold higher odds of a PSG-defined sleep disorder than controls [57]. A multidimensional “sleep health” composite (actigraphy + symptoms) aligned with disability and daily anxiety in adolescent females with frequent migraine [58]. Population analyses also suggested subtype-specific sleep disturbance patterns in adolescents [59].

Imaging/psychophysiology work also ties sleep-related processing to migraine in adolescents, complementing PSG and actigraphy findings [60].

Earlier clinical data additionally linked sleep disturbance to headache-related disability in youth cohorts [61]. In a community-based Turkish sample (N = 4151), headache co-occurred with restless legs syndrome (RLS). Adolescents with RLS showed higher Epworth Sleepiness Scale scores and poorer academic success, with the strongest signal in migraine [62].

#### 3.1.4. Neurocognitive Profile and Brain Function

Neuropsychological testing indicated domain-specific weaknesses. Children with migraine without aura performed below typically developing peers on NEPSY-II executive, language, memory, sensorimotor, and visuospatial domains, with some relative strengths in visual attention; several scores covaried with attack frequency/intensity [63]. Case–control work found poorer verbal memory, attention/executive performance, and slower information processing in adolescents with migraine [64]. Visual attention appeared particularly sensitive: untreated migraine groups underperformed across multiple measures (Trail Making, Letter Cancellation, computerized attention), while youths on effective prophylaxis resembled controls—suggesting potential reversibility [65]. In contrast, visually guided associative learning (acquired equivalence) was preserved in newly diagnosed pediatric migraine without aura [66]. Outside vision, central auditory processing differences emerged in duration patterning and speech-in-noise tasks [67].

Resting-state fMRI suggested broader connectivity alterations in adolescents (e.g., default-mode and cerebellar networks) relative to young adults with migraine, with age-specific relationships to duration/frequency [68].

Executive function weaknesses and lower QoL during the COVID-19 period in children with migraine have been confirmed in a BRIEF-based case–control study [69].

Beyond core EF, social cognition skills (emotion recognition, theory of mind) were poorer in migraine and correlated with EF, versus healthy peers [70].

These neuropsychological and imaging findings indicate selective, interictal differences in attention/executive and network connectivity among youths with migraine.

#### 3.1.5. Pain-Related Cognitions, Affective Traits, and Personality

Osmophobia and odor-triggered attacks were highly specific for migraine and marked a more severe clinical profile [71]; in tertiary clinics, osmophobia correlated with higher disability (PedMIDAS), anxiety/depression, and pain catastrophizing [72]. Pain catastrophizing—though not always different between episodic and chronic—tracked poorer QoL, higher anxiety/depression, and sensitization signs [73]. Health mindsets were clinically meaningful: a “growth” health mindset related to lower threat appraisal, less passive coping, and better QoL in pediatric chronic headache [74]. Early maladaptive schemas were elevated in adolescents with migraine—especially in chronic forms—and associated with disability and internalizing symptoms; patterns differed between episodic and chronic groups [75,76]. Personality/affect studies have pointed to greater internalizing problems and distinct affect regulation on performance-based measures [77]. Alexithymia findings were mixed across diagnoses but included higher scores in adolescents and mothers with migraine [78] and, in other samples, higher alexithymia in TTH than migraine [79]; focused comparisons in migraine without aura reported consistently elevated alexithymia [80,81]. During the COVID-19 period, adolescents with headache showed increased conscientiousness and fewer reported school/bullying problems in small, uncontrolled series [82]. Coping styles also mattered: adolescents with both chronic headache and mental health problems relied more on internal/avoidant strategies and less on help-seeking than healthy peers [83].

Sensory avoidance predicted disability (PedMIDAS) and was linked to catastrophizing and lower sensation-seeking in adolescents with migraine [84].

Parenting stress tracked with adolescent internalizing symptoms and headache frequency in primary headache [85].

#### 3.1.6. Quality of Life, School Participation, and Health-Related Behaviors (Including Eating-Related Symptoms)

Population studies showed large, graded decrements in health-related QoL and functioning with increasing headache frequency, together with measurable impacts on absenteeism and activity restriction [86,87]; Spanish adolescents with recurrent headache reported higher SDQ difficulties and disability [88], and clinic samples also showed poorer QoL in migraine vs. controls [45,89]. Qualitative interviews underscored pervasive impacts on emotional well-being, daily routines, and the school environment [90]. In low-resource contexts, 1-year prevalence and burden remained high, with migraine exerting the largest impact on QoL and schooling [91]. Disordered-eating symptoms (EAT-26/BITE)—particularly bulimic features—were more frequent in adolescents with migraine, especially females [92].

Recent outpatient series reinforce the severity gradient and service needs in pediatric migraine [93].

In clinical comparisons, the co-occurrence of primary headache with LD was associated with higher anxiety rates and—in chronic tension-type headache—substantially greater school absenteeism, underscoring an educational risk subgroup [54].

#### 3.1.7. Measures Used Across Studies

Psychopathology was assessed with SCARED [45,94], STAI/STAIC [34,89], and CDI/RCMAS-2 [45,61]. For self-management and family context, studies employed the Psychosocial Assessment Tool (PAT) [95] and the Parenting Attitude Test for Youth (PAT-Y) [96]. Headache burden and HRQoL commonly used PedMIDAS, FDI, PedsQL, KIDSCREEN, and HARDSHIP [61,86,88]. Sleep was captured with SDSC/SHIP, actigraphy, and polysomnography [55,56,57,58,59]. Neurocognition relied on NEPSY-II, BRIEF, Wechsler/Leiter-3, and domain tasks [63,64,65,66,67,97].

The SDQ+PedMIDAS bundle effectively differentiated emotional/behavioral burden and disability between migraine, TTH, and controls in a disadvantaged school cohort, offering a pragmatic screening template [48]. Additional practical screens used in clinics include brief CDI/RCMAS-based triage [42].

Overall, pediatric primary headaches co-occur with higher internalizing symptomatology, frequent ADHD/learning problems, sleep disturbance, and selective neurocognitive inefficiencies (especially attention/executive and auditory/visual attention), with osmophobia, catastrophizing, and maladaptive schemas marking more severe clinical profiles. The cumulative impact on QoL and school participation is substantial across settings—including low-resource contexts—and a consistent assessment toolkit now supports cross-study comparability and clinical translation. The expanded synthesis integrates additional experimental and observational studies cataloged in Table S1 (parts 1–2) (e.g., [41,44,45,46,60,61,69,70,84,85,93]).

### 3.2. Results from Reviews and Meta-Analyses (Secondary Evidence)

To avoid double counting, the review/meta-analytic findings are summarized as higher-level evidence and not merged with individual primary estimates already reported in Section 3.1.

Across narrative and systematic reviews (including meta-analyses), pediatric primary headaches were consistently linked to emotional–behavioral symptoms, sleep disturbance, neurodevelopmental conditions (notably ADHD), school/academic impacts, and selective neurocognitive differences, with several reviews proposing shared biobehavioral mechanisms.

#### 3.2.1. Psychopathology (Anxiety/Depression) and Headache

A meta-analysis of 80 observational studies (51 meta-analyzed) found significantly higher anxiety (large effect) and depressive symptoms (moderate effect) in youths with migraine versus healthy controls; odds of clinical anxiety/depression were about doubled in migraine, with similar effects in clinical and community samples [98].

Narrative syntheses similarly concluded that internalizing symptoms co-occur with pediatric migraine/TTH and that risk increases with headache frequency, while emphasizing likely bidirectionality [99,100].

In contrast, a critical narrative appraisal argued that many clinic samples show only slightly higher anxiety/depression scores that are rarely in the clinical range, with no clear population-level elevation, highlighting measurement confounding and selection bias [101].

A targeted systematic review of children ≤ 12 years reported robust associations between headaches and both internalizing and externalizing symptoms, and frequent links with stressful environments/adverse events (e.g., bullying) and family conflict, with sleep problems commonly co-occurring [102].

A broader narrative review cataloged emotional problems, noting alexithymia, anxiety/depression, and neurodevelopmental disorders as recurring correlates [21].

A contemporary clinical narrative review recommends universal screening for anxiety and depression in all children with migraine and highlights equity gaps and limited pediatric-specific evidence for several treatments [103].

#### 3.2.2. Sleep and Headache

A systematic review of 12 studies (*n* = 16,474; ages 2–18) concluded that headaches and sleep disorders are strongly and bidirectionally associated; worse headache (frequency/intensity) paralleled more severe sleep problems. Methodologically, no study used ICSD diagnostic criteria, relying instead on questionnaires [104].

Narrative work converged on a mutual relationship (sleep disturbances can trigger headaches; sleep can terminate migraine), with frequent comorbidity with parasomnias, sleep-disordered breathing, restless legs syndrome, and potential early-life origins (e.g., infantile colic) [105].

#### 3.2.3. ADHD and Other Neurodevelopmental Conditions

A PRISMA meta-analysis (11 studies meta-analyzed) found a specific association between ADHD and migraine (OR ≈ 1.32), with no significant association for ADHD with TTH or “headache” in general; heterogeneity and potential stimulant confounding were noted [106].

Narrative reviews outlined plausible shared mechanisms—dopaminergic dysfunction, brain iron metabolism, and sleep regulation—as overlapping pathways between headache and ADHD, while describing conflicting epidemiologic evidence [107].

A broader narrative synthesis further noted frequent comorbidity with ASD and emphasized diagnostic challenges in neurodevelopmental groups [21,108].

#### 3.2.4. Neurocognition

A PRISMA meta-analysis of 16 studies reported poorer overall neuropsychological performance in pediatric primary headache (Hedges’ g = −0.31), with deficits across motor, executive, learning/memory, language, processing speed, intelligence, and visuospatial domains; the effects were primarily driven by migraine samples (non-significant for TTH) [23].

A narrative neurocognitive review concluded that despite normal general intelligence, interictal profiles in pediatric migraine often show non-homogeneous patterns with difficulties in verbal skills, attention, processing speed, and verbal memory, potentially impacting school performance [25].

#### 3.2.5. School/Academics, Learning, and QoL

Narrative syntheses reported that learning disabilities are common in children with primary headaches (especially migraine), with specific deficits in memory, attention, and processing speed and multifactorial contributors [109].

Reviews focused on education described an interplay of ADHD, sleep problems, anxiety/depression, and psychosocial stressors (e.g., bullying, perfectionism) in academic difficulties [110].

A quality-of-life review concluded that pediatric primary headaches negatively affect QoL across domains (psychological, academic, social), often more than other chronic illnesses, and highlighted school absenteeism and impaired peer relations [111].

#### 3.2.6. Obesity and Migraine

A narrative review summarized an association between obesity and pediatric headaches, with less certainty for a migraine-specific link; proposed mechanisms included hypothalamic dysfunction, serotonergic alterations, and pro-inflammatory states, with some signals that weight loss may reduce headache frequency [112].

#### 3.2.7. Integrated/Biobehavioral Models

A comprehensive narrative review described migraine in childhood as a biobehavioral/psychosomatic disorder arising from interactions between genetic/neurological factors (e.g., DMN/insula alterations), psychological factors (stress, internalizing symptoms, alexithymia), and family/environmental contributors (dysfunctional dynamics, insecure attachment, parental psychiatric illness, ACEs) [113].

An additional narrative overview emphasized shared mechanisms with psychiatric comorbidities (e.g., serotonin, genetics), diagnostic complexity in ASD/ADHD, and treatment interactions that underscore integrated care pathways [108].

#### 3.2.8. Methodological Limitations and Research Agenda

Systematic reviews noted heterogeneity of measures, predominance of cross-sectional designs, and limited adjustment for confounders; some domains (e.g., trauma- and stressor-related disorders, screen-time impacts) were under-studied [98,104,110].

Several reviews called for longitudinal designs, use of validated sleep/psychiatric diagnostics (including ICSD criteria), larger/representative samples, and standardized neurocognitive batteries to enable comparability [23,25,99,104].

## 4. Discussion

Overall, primary data delineate developmental-age headaches as conditions with a systemic impact. Beyond the pain itself, higher levels of internalizing symptoms, a greater prevalence of neurodevelopmental comorbidities (particularly ADHD), and a sleep disturbance profile consistently emerge as correlated of—and are associated with—the condition’s persistence and disability [98,104].

This co-occurrence is repeatedly described as bidirectional in narrative and PRISMA reviews of pediatric sleep–headache links and psychiatric comorbidity [104,105], while the co-occurrence with internalizing psychopathology is consistently reported [99].

**Figure 3:**
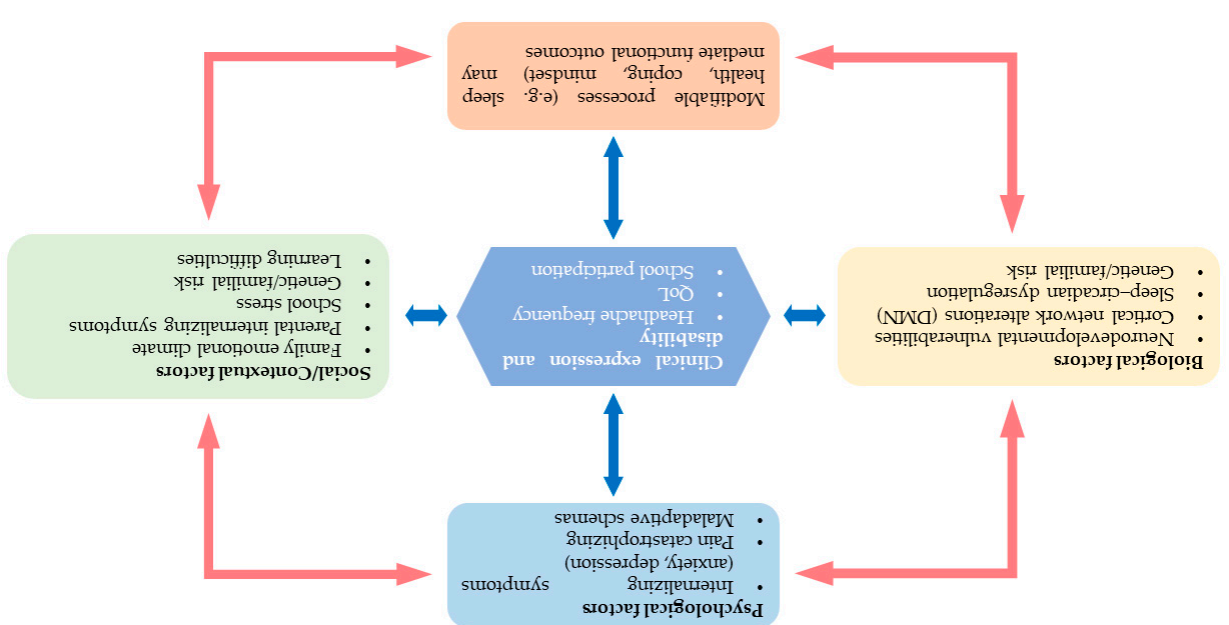
Conceptual model of biopsychosocial interactions in pediatric primary headaches. Boxes represent biological (neurodevelopmental vulnerabilities, sleep–circadian dysfunction), psychological (anxiety/depression, catastrophizing, maladaptive schemas), and social/family factors (family emotional climate, school stress, learning difficulties). Bidirectional arrows indicate reciprocal influences among domains and between each domain and the central clinical outcome (clinical expression and disability: headache frequency, quality of life, school participation). Modifiable processes (e.g., sleep health, coping, mindset) may mediate functional outcomes.

In parallel, neurocognitive differences—of small to moderate magnitude and concentrated in attentional-executive domains, verbal memory, and processing speed—are associated with functional outcomes beyond the nociceptive dimension [64,67,114]. A recent meta-analysis reports an overall deficit (Hedges’ g ≈ −0.31), driven by migraine rather than TTH and spanning multiple domains [23], with converging narrative evidence [25]. A biobehavioral framing that integrates neural network alterations (e.g., DMN), psychological traits, and family factors is also endorsed [113]. Taken together, the evidence supports a biopsychosocial model in which neurobiological vulnerabilities and sleep–circadian dysregulation interface with emotional–cognitive factors and family–school contexts to shape clinical expression and daily functioning (Figure 3).

Taken together, the evidence supports a biopsychosocial model: neurobiological vulnerabilities and sleep–circadian dysregulations are thought to interact with emotional-cognitive factors and family–school contexts and may modulate clinical expression and daily functioning [113]. Broader psychiatric–migraine syntheses similarly argue for integrated assessment and care [108].

Clinic-based comparisons also show higher CBCL/SAFA scores in youth with primary headaches versus healthy peers—without clear differences between migraine and other subtypes—and a mother–child concordance for internalizing symptoms alongside a stronger paternal-line family recurrence signal [47]. Consistently, SDQ data show more emotional problems in migraine vs. TTH in outpatient cohorts [115], while both phenotypes display high psychiatric comorbidity in clinic samples [36].

In disadvantaged school settings, adolescents with migraine display higher SDQ emotional problems and total difficulties than those with TTH or no headache, together with greater pain severity and disability [48]. Review work on “pediatric migraine and academics” reinforces the contribution of sleep, internalizing symptoms, and ADHD to school difficulties [110]. Functionally, the impact includes lower quality of life and difficulties with school participation with a clear severity gradient. Psychological and functional impairments increase as attack frequency or chronicity rises, and the most dysfunctional profiles are seen in chronic forms and/or medication overuse (e.g., [28,37,86,93,116,117]). Large database and clinic studies add that migraine in adolescence predicts a higher 10-year incidence of affective and stress-related disorders [46] and that obesity frequently co-occurs with pediatric migraine with elevated OSAS rates [118].

Co-occurring learning disabilities identify a high-risk subgroup: in clinical comparisons, headache+LD relates to higher anxiety, and among chronic TTH specifically, LD is associated with markedly higher school absenteeism (50% vs. 22.2%) [54]. A narrative review specifically connects primary headaches with LD and executive/attention weaknesses relevant to classroom performance [109].

### 4.1. Psychopathology

Primary headaches in children and adolescents appear to be associated with greater internalizing symptomatology (e.g., anxiety and depression) (e.g., [38,41,42,116]), neurodevelopmental comorbidities (particularly ADHD) (e.g., [49,51,53]), and numerous sleep problems (in a complex and multifaceted bidirectional relationship) (e.g., [45,58,59]). Psychiatric-clinic and population data converge: children evaluated for psychiatric symptoms report more headaches [34], and those with chronic headache plus mental health problems show distinct coping profiles [83].

Contemporary clinical guidance further recommends that all children with migraine be screened for anxiety and depression and highlights equity gaps and limited pediatric-specific evidence for many treatments [103].

The most parsimonious interpretation of the primary data suggests the confluence of multiple mechanisms. From a neurofunctional perspective, adolescents with migraine exhibit resting-state connectivity differences in cognitive–affective networks, with the involvement of the DMN and cerebellum, a profile compatible with the attentional and emotional correlates observed at the behavioral level [68]. Reviews of pediatric migraine also describe DMN/insula alterations within a biobehavioral framework [113].

### 4.2. Sleep

Sleep emerges as a central node. Polysomnography and actigraphy document architectural alterations (e.g., variations in N2/N3) and a more generally unfavorable “sleep health” profile. These patterns are associated with greater disability (PedMIDAS) and daily anxiety, even beyond pain intensity [56,57,58,59]. In clinical samples, higher CBCL internalizing scores map onto specific SDSC sleep-disturbance clusters (e.g., difficulty initiating and maintaining sleep, arousal, sleep–wake transition) [55], and syntheses reaffirm strong sleep–headache comorbidity [104,105]. Neurophysiological evidence complements these signals, linking sleep-related pain processing to adolescent migraine and strengthening the rationale for systematic sleep screening and intervention [60]. Case–control neuroimaging with concurrent sleep measures similarly ties subjective insomnia to higher disability [60].

### 4.3. Family and Process-Level Factors

Process-level factors also modulate outcomes: pain catastrophizing, osmophobia, and sensory avoidance profiles predict disability and quality of life; in adolescents with chronic forms, more marked maladaptive schemas (understood as dispositional traits) are observed, whereas a growth–health mindset relates to lower perceived threat, less passive coping, and better quality of life [18,71,72,73,74,75,76,84]. Coping/style results echo population data on coping in chronic headache [83].

Personality-level correlates (e.g., neuroticism) and transdiagnostic fear-avoidance processes show medium associations with disability, highlighting cognitive–affective targets for intervention [16,17].

Sensory avoidance independently predicts disability and couples with catastrophizing and lower sensation seeking, suggesting a tractable behavioral target [84]. Findings replicate in independent adolescent clinic cohorts [75,76].Family factors contribute independently: psychosocial risk shows six-month predictive validity for child, parent, and family outcomes; in mother–child dyads, higher attack frequency is associated with greater emotion regulation difficulties, and the child’s internalizing symptoms are explained by the child’s negative affect, parental internalizing symptoms, and less verbal sharing of emotions; additionally, higher alexithymic traits have been described in pediatric TTH compared to migraine and controls, with a mother–child correlation present in migraine and controls but not in TTH [18,79,95]. Age-graded differences in perceived parenting attitudes also relate to headache burden [96].

This pattern is consistent with the hypothesis that process-level targets (e.g., catastrophizing/avoidance, sleep health) may be more closely tied to changes in clinical outcomes than global internalizing symptom levels per se (see [119]).

The mother–child internalizing concordance and paternal-line recurrence observed in clinic samples argue for routine, family-centered assessment (structured family history; parent-reported measures) alongside child-reported screening [47].

In the school context, qualitative and school-based studies report bright lights and noise as frequent triggers, with repercussions in terms of absences and concentration difficulties; in school and population samples, higher rates of headache/migraine-related absenteeism are documented [33,86,89,90,91]. Marked school and social impairment is also described in the pediatric NDPH series [117].

For TTH, school/population studies indicate lower school-related quality of life and more missed school days as frequency increases, confirming a gradient of burden and activity limitations, even in the absence of the typical sensory profile of migraine [86,87].

Given the stronger absenteeism signal when LD co-occurs—especially in chronic TTH—educational liaisons (screening for LD; tailored accommodations) should be embedded within headache care pathways [54]. See also the LD–headache linkage outlined in [109].

### 4.4. Neurocognition

Regarding neurocognition, primary studies converge on small to medium, domain-specific differences, with sometimes discordant outcomes. Compared to controls, lower performance is frequently reported in verbal memory, attention/executive functions, and processing speed [64,68,69,97]. Meta-analytic evidence confirms worse overall performance in pediatric primary headache—especially migraine—across multiple domains [23], while narrative reviews emphasize non-homogeneous interictal profiles [25].

In a broad assessment with the NEPSY-II, lower scores are observed in several subdomains, alongside circumscribed strengths (e.g., visual attention and memory for faces), confirming a profile that is not globally deficient [63]. A clinically relevant finding is that attentional deficits appear more pronounced in untreated youth and tend to normalize in patients on prophylaxis, consistent with a possible association with treatment response [65]. On the perceptual level, differences have been described in central auditory processing on some tests (DPT/SSI/NVDT) but not others (gap-in-noise), and in newly diagnosed subjects, visual associative learning appears preserved, confirming the heterogeneity of patterns depending on the domain and clinical stage [66,67]. In severe inpatient cohorts (status migrainosus), comorbid ADHD and severe anxiety track with poorer treatment response, underscoring neurocognitive/psychological complexity in refractory cases [52].

It should also be emphasized that almost all neurocognitive assessments were conducted in the interictal phase; the absence of peri-ictal data in the included primary studies limits inference about the phase-specific dynamics of cognitive profiles.

The comparison between phenotypes helps clarify clinical specificity. In real-world outpatient settings, migraine, compared to TTH, is more often associated with nausea, photophobia/phonophobia, moderate to severe pain, greater analgesic use, and greater impairment of daily activities. TTH, while lacking the more pronounced sensory profile, still entails substantial repercussions on quality of life and functioning [11,45,86,89]. Across both phenotypes, clinic samples report high psychiatric comorbidity burdens [36].

Sleep disturbances can be more pronounced in TTH/new daily persistent headache (NDPH) than in migraine and are associated with both disability and emotional symptoms across phenotypes [61]. Moreover, adolescents with headache+RLS reported greater daytime sleepiness and lower academic success, underscoring sleepiness as a plausible mediator of school impact [62].

However, recurring limitations warrant caution in making causal inferences. Prospectively, baseline anxiety and depressive symptoms did not predict changes in either headache frequency or migraine-related disability, underscoring the need to test mechanistic mediators directly [119]. A combined reading of the primary studies shows a clear prevalence of cross-sectional designs, often small samples, and heterogeneous measures across studies and domains. Typical examples include neurocognitive assessments (NEPSY-II vs. various test batteries, with not always convergent results: [63,64]), auditory processing profiles (differences on some tests but not others: [67]), and resting-state neuroconnectivity studies (rs-fMRI in small samples: [68]). Even in studies on sleep and functioning, potential confounders (internalizing/ADHD comorbidities, medications, sleep quality, socioeconomic status) are not always controlled for consistently across studies [56,57,61]. These elements, together with the small to medium magnitude of the reported cognitive differences and the variability of the results, recommend caution in causal inference and highlight the need for longitudinal studies with standardized batteries. Methodological caution from narrative critiques (e.g., measurement issues, clinic-sample selection) reinforces these caveats [100,101].

Available syntheses (systematic, meta-analytic, and narrative reviews) converge on several key points. On the psychopathological front, a meta-analysis of 80 studies (51 meta-analyzed) documents higher levels of anxiety (large effect) and depression (moderate effect) in youth with migraine compared to controls, with approximately doubled odds of a clinical diagnosis; the results are similar in clinical and community samples [98]. Narrative reviews indicate that internalizing symptoms and headache co-occur and that the risk increases with attack frequency [99,100]. The relationship with sleep is reaffirmed by a PRISMA systematic review (12 studies; *n* ≈ 16.5 k): a strong, bidirectional association between sleep disorders and pediatric headache, with sleep severity paralleling headache frequency/intensity; methodologically, no study used ICSD criteria, relying instead on questionnaires [104,105]. In the neurocognitive domain, a meta-analysis reports worse overall performance in children/adolescents with primary headache (Hedges’ g ≈ −0.31), with differences across motor, executive, memory/learning, language, processing speed, intelligence, and visuospatial domains; the effects are driven by migraine samples, while they are not significant for TTH [23]. Finally, reviews on ADHD support a specific association with migraine (OR ≈ 1.3), with heterogeneity and possible confounding from stimulant medication [106].

Complementing these syntheses, a recent clinical narrative review endorses universal anxiety/depression screening in pediatric migraine and underscores the need for equitable access and pediatric-specific evidence, with many treatments extrapolated from adults [103].

Alongside these convergences, narrative and systematic reviews highlight limitations to consider in interpretation: some of the internalizing differences in clinical samples fall within subclinical ranges, with possible selection and measurement biases [101]; the literature on sleep–headache suffers from heterogeneity of tools and scarce use of standardized diagnostic criteria [104]; inter-ictal neurocognitive profiles are not homogeneous across tests and samples, with not always convergent results, and require more standardized batteries [25]. Reviews also call for clarity on school-context variables (e.g., bullying, digital load) and trauma/adverse childhood experiences (ACEs) [102,110].

In short, methodological quality and variability of measures condition the strength of inferences and motivate longitudinal and phase-specific designs.

### 4.5. Clinical and Educational Implications

On the service delivery front, secondary evidence supports integrated neuro-psycho-educational pathways: structured screening for emotional symptoms, sleep, and comorbidities, and school–health interoperability (accommodations and gradual re-entry), with the development of modular packages (sleep hygiene, CBT for pain/anxiety, executive training, parent training) and shared functional outcomes [100,102,104,105]. General QoL reviews further emphasize school absenteeism and psychosocial impact as key clinical targets [111]. Importantly, clinical data show that a considerable share of pediatric headache patients present psychiatric comorbidities (especially anxiety and depression), highlighting the need for routine screening [120]. Practical screening can leverage brief tools already validated in disadvantaged contexts (e.g., SDQ+PedMIDAS pairing) to triage psychological burden and disability where specialist resources are scarce [48].

Several areas remain under-researched: the role of trauma/stress (noted as a gap in reviews), screen time and digital load, and dietary habits; furthermore, the distinction between phenotypic subtypes and ictal phases is often incomplete. Quantitative syntheses on pediatric TTH are few, and signals from meta-analyses indicate non-significant effects for TTH compared to controls [23]. Cluster headache in the pediatric population remains under-represented in reviews, with scarce dedicated synthesis [5,15]. Reviews on school/academic impact draw attention to digital and psychosocial factors (e.g., bullying), but highlight limited data and the need for prospective studies [110]; preliminary signals on screen time come from small/retrospective or COVID-era studies [53,121]. Obesity and sleep-related breathing disorders represent additional, potentially modifiable comorbidity targets [112,118]. These gaps delineate priorities for future systematic reviews and prospective registries.

This review integrates primary studies and secondary evidence while maintaining a clear distinction between the two types of sources. This methodological approach prevents the risk of double counting and allows for a clear discernment between data originating from original research and those drawn from literature syntheses. The adopted perspective is multidimensional, encompassing the assessment of the emotional profile, sleep disturbances (including objective measures), school functioning and quality of life (QoL), neurocognitive aspects, family and dyadic dynamics, and a comparison between migraine and tension-type headache (TTH). Furthermore, a mapping of clinically applicable assessment tools is provided (e.g., PedMIDAS/FDI, PedsQL/KIDSCREEN, CBCL, SDSC/actigraphy, NEPSY-II/BRIEF, PAT), which supports the implementation of a structured assessment pathway within healthcare services. Finally, the review places specific emphasis on modifiable processes (e.g., catastrophizing, trigger avoidance, sleep health, schemas, mindset), explicitly linking the research findings to concrete intervention targets.

### 4.6. Methodological Considerations and Limitations

This paper is a narrative review and, as such, has several methodological limitations. No protocol was pre-registered, and neither was a formal risk of bias assessment conducted systematically. The search strategy relied primarily on two provided sets of articles and on targeted retrievals and may therefore be subject to incompleteness as well as publication and linguistic biases. The conceptual and instrumental heterogeneity across studies precluded robust aggregate estimates (i.e., no meta-analysis was performed); thus, the synthesis is qualitative and does not model confounders in a comparable way across studies. The decision to separate primary studies from reviews and meta-analyses reduces, but does not entirely eliminate, the risk of double counting, as the same datasets may appear in multiple secondary syntheses. Finally, some domains (e.g., TTH, cluster headache, digital media/lifestyle habits) are under-represented even within our search perimeter, limiting the generalizability of the conclusions to these subgroups. Moreover, the restriction to English-language publications may have introduced a potential publication bias, limiting the inclusion of relevant studies published in other languages.

Another limitation concerns the lack of formal subgroup or cross-regional analyses. Due to the heterogeneity of available studies and inconsistent reporting of demographic information, comparisons across countries, age groups, gender, and ethnicity could not be systematically performed. Future research should better characterize these sources of variability to clarify potential developmental and sociocultural moderators.

In addition, given the predominantly cross-sectional and observational nature of the included studies, the reported associations cannot be interpreted as causal. Terms such as “associated with” or “linked to” are used throughout the text to emphasize correlational, rather than directional, relationships.

## 5. Conclusions

In the management of children and adolescents with primary headache, it is imperative to look beyond the sole dimension of pain. Internalizing symptoms, quality of life, and school participation account for a significant portion of the burden [86,98], while sleep often represents a clinical nexus that fuels disability and daily anxiety [56,57,58,61]. Reviews underscore a bidirectional sleep–headache link and recommend routine sleep screening in pediatric care [104,105]. A multidimensional assessment should therefore include, alongside a pain diary and frequency tracking, brief, validated measures of anxiety/depression [98], functional disability, and QoL [86], a sleep screening with SDSC and—when indicated—actigraphy [56,57,58,61], a core neurocognitive profile targeted to attention/executive functions, processing speed, and verbal memory [63,64], as well as a family-centered assessment including structured family history and parent-reported measures [18,47,95]. When school functioning is affected—particularly in chronic TTH—screening for learning disabilities and liaison with education services should be included [54].

Operationally, care should follow a stepped-care model: education and sleep hygiene for all; CBT modules focused on catastrophizing, avoidance, and management of sensory/environmental triggers when disability persists [72,73,74,84]; and parental involvement and school coordination for simple accommodations (breaks, light/noise management, gradual re-entry) [33,86,87,90,91]. Stratification by frequency—and attention to potential medication overuse—should guide intensity [117]; comorbidities such as ADHD must be identified and managed collaboratively [49,51]. Neurocognitive differences are typically small to moderate and more evident in migraine than TTH; monitoring should prioritize functional outcomes and process-level targets (e.g., sleep health, catastrophizing/avoidance) rather than pain days alone [23,104]. Follow-up should monitor functional outcomes (e.g., PedMIDAS/FDI, QoL, school absenteeism) in addition to headache days; if these do not improve, the care plan should be recalibrated [86]. Universal screening for anxiety/depression is advisable in pediatric migraine pathways and supports timely access to appropriate care [103].

## Figures and Tables

**Table 1:** Search queries used for each database with the corresponding filters applied.

Database	Search Query	Filters
Scopus	(“primary headache” OR migraine OR “tension-type headache” OR “cluster headache”) AND (child* OR adolescen* OR pediatric OR paediatric OR youth) AND (psychopathology* OR neuropsychology* OR “cognitive function*” OR “executive function*” OR attention OR memory OR “behavioral problem*” OR “emotional problem*” OR “mental health”)	Language: English Title/abstract/keywords Last 10 years
Embase	(‘primary headache’:ti,ab,kw OR migraine:ti,ab,kw OR ‘tension-type headache’:ti,ab,kw OR ‘cluster headache’:ti,ab,kw) AND (child*:ti,ab,kw OR adolescen*:ti,ab,kw OR pediatric:ti,ab,kw OR paediatric:ti,ab,kw OR youth:ti,ab,kw) AND (psychopathology:ti,ab,kw OR neuropsychology*:ti,ab,kw OR ‘cognitive function*’:ti,ab,kw OR ‘executive function*’:ti,ab,kw OR attention:ti,ab,kw OR memory:ti,ab,kw OR ‘behavioral problem*’:ti,ab,kw OR ‘emotional problem*’:ti,ab,kw OR ‘mental health’:ti,ab,kw) AND [2015–2025]/py AND [english]/lim	Language: English Title/abstract/keywords Last 10 years
PubMed	((“Primary Headache Disorders”[MeSH Terms] OR “Migraine Disorders”[MeSH Terms] OR “Tension-Type Headache”[MeSH Terms] OR “Cluster Headache”[MeSH Terms] OR “primary headache”[Title/Abstract] OR migraine[Title/Abstract] OR “tension-type headache”[Title/Abstract] OR “cluster headache”[Title/Abstract]) AND (“Child”[MeSH Terms] OR “Adolescent”[MeSH Terms] OR child*[Title/Abstract] OR adolescen*[Title/Abstract] OR pediatric[Title/Abstract] OR paediatric[Title/Abstract] OR youth[Title/Abstract]) AND (“Psychopathology”[MeSH Terms] OR “Neuropsychology”[MeSH Terms] OR “Cognition”[MeSH Terms] OR “Mental Disorders”[MeSH Terms] OR “Executive Function”[MeSH Terms] OR psychopathology[Title/Abstract] OR neuropsychology*[Title/Abstract] OR “cognitive function*”[Title/Abstract] OR “executive function*”[Title/Abstract] OR attention[Title/Abstract] OR memory[Title/Abstract] OR “behavioral problem*”[Title/Abstract] OR “emotional problem*”[Title/Abstract] OR “mental health”[Title/Abstract]))	Language: English Title/abstract/keywords Last 10 years

## Data Availability

Data will be made available on request from the corresponding author. Sharing on request ensures a version-controlled, well-documented bundle of screening/extraction materials and is preferable given their length/volume. No primary data were collected; all sources are publicly available.

## Supplementary Materials

The following supporting information can be downloaded at https://www.mdpi.com/article/10.3390/life15101641/s1: Tables S1 and S2 are provided in a separate Supplementary Materials file. Table S1 (parts 1–2): Characteristics of primary empirical studies (aim, sample[s], control group, psychopathological and cognitive variables, instruments, design, main results, limitations). Table S2 (parts 1–2): Characteristics of reviews and meta-analyses (purpose, questions, methods, included evidence, synthesized results, conclusions, limitations, and future directions). Due to formatting constraints, each table is split into two parts; the content is continuous across parts.

## Abbreviations

The following abbreviations are used in this manuscript:

ICHD-3	International Classification of Headache Disorders, 3rd edition
TTH	Tension-Type Headache
YLDs	Years Lived with Disability
ASD	Autism Spectrum Disorder
ADHD	Attention Deficit Hyperactivity Disorder
QoL	Quality of Life
TAC	Trigeminal Autonomic Cephalalgia
LD	Learning Disabilities
RLS	Restless Legs Syndrome
PSG	Polysomnography
ACE	Adverse Childhood Experience
OSAS	Obstructive Sleep Apnea Syndrome
