# Supplementary material for: Beyond the Pain: A Critical Examination of the Psychopathological and Neuropsychological Dimensions of Primary Headaches in Pediatric Populations
Source: Life (Basel). 2025 Oct 21;15(10):1641. doi: 10.3390/life15101641 (PMC12565120; doi:10.3390/life15101641)
Supplement: Supplementary file 1 [file life-15-01641-s001.zip › life-3931313-supplementary.pdf]

**Table S1 (part 1 of 2).** Primary studies that met the inclusion criteria. *Note: Table S1 is presented in two parts; see part 2 for continuation.*

| Authors and Year | Title | Purpose | Experimental Group(s) | Control Group | Tools Used |
|---|---|---|---|---|---|
| **Caruso et al. (2022)** | Health Mindsets in Pediatric Chronic Headache | To evaluate whether growth health mindsets are related to adaptive outcomes (such as cognitive appraisals, coping, quality of life, life satisfaction, and functional impairment) in children and adolescents with chronic headache. | 88 children and adolescents (age 10-17; mean age 15.2±1.9 years) with a diagnosis of headache or migraine. One parent for each child also participated. | This study did not include a healthy control group for comparison. | A series of self-report questionnaires were used, including the Health Mindset Scale, measures of Cognitive Appraisals (based on vignettes), the Pain Response Inventory (PRI) for general coping, the Pediatric Quality of Life Inventory (PedsQL), the Satisfaction with Life Scale-Children (SWLS-C), and the Functional Disability Inventory (FDI). |
| **Açıkel et al. (2021)** | Comparison of Children with Migraine and Those with Tension-Type Headache for Psychiatric Symptoms and Quality of Life | To compare psychiatric symptoms and quality of life (QoL) in children with migraine, tension-type headache (TTH), and a healthy control group. | Group 1: 37 children with migraine; group 2: 22 children with tension-type headache (TTH). Age range for both groups: 8-18 years. | 35 healthy controls (age 8-18 years) | Child Depression Inventory (CDI); Screen for Child Anxiety Related Emotional Disorders (SCARED); Children's Somatization Inventory (CSI); Children's Sleep Habits Questionnaire (CSHQ); Childhood Anxiety Sensitivity Index (CASI); Pediatric Quality of Life Inventory, Parent version (PedsQL-P). |
| **Agessi et al. (2017)** | Auditory Processing in Children with Migraine: A Controlled Study | To investigate and compare central auditory processing performance in children who have migraines with a control group of children who do not experience headaches. | 14 children, aged 8 to 12 years, who were diagnosed with migraine (with or without aura). | 14 children of the same age range who had no history of headaches. | Gaps-in-Noise (GIN): To test the ability to detect small silences in noise; Duration Pattern Test (DPT): To test the ability to recognize patterns of long and short sound; Synthetic Sentence Identification (SSI): To test the ability to identify sentences in the presence of competing noise; Nonverbal Dichotic Test (NVDT): To test the ability to focus on sounds presented to one ear while ignoring sounds in the other. |
| **Güler Aksu et al. (2022)** | Early maladaptive schemas differing according to sex may contribute to migraine among the youth | To examine how early maladaptive schemas (EMS)—which are deeply ingrained, negative patterns of thinking—differ between male and female adolescents with migraine. | 171 adolescents (115 females, 56 males) between the ages of 12 and 18, all diagnosed with migraine. | This study did not include a control group of adolescents without migraine. It only compared males and females within the migraine group. | Headache Questionnaire Form: To gather detailed information about the clinical characteristics of migraines (frequency, severity, symptoms, etc.); Early Maladaptive Schema Questionnaires Set for Children and Adolescents (SQS): A self-report questionnaire used to identify and measure 15 different early maladaptive schemas. |

**Table S1 (part 1 of 2).** Primary studies that met the inclusion criteria. *Note: Table S1 is presented in two parts; see part 2 for continuation.*

| | | | | | |
|---|---|---|---|---|---|
| **Güler Aksu et al. (2023)** | Early maladaptive schemas in episodic and chronic migraine in adolescents | To investigate and compare early maladaptive schemas (EMS) in adolescents with episodic migraine (EM) versus those with chronic migraine (CM). | Group 1: 140 adolescents (aged 12-18) with episodic migraine (EM) Group 2: 27 adolescents (aged 12-18) with chronic migraine (CM) | This study did not include a healthy control group. It only compared the two different migraine groups. | Headache Questionnaire Form: To collect data on headache characteristics. Kovacs Children's Depression Inventory (CDI): To measure depressive symptoms. Screen for Child Anxiety Related Emotional Disorders (SCARED): To measure anxiety symptoms. Early Maladaptive Schema (EMS) Questionnaire: To measure deeply ingrained negative thought patterns. |
| **Amouroux et al. (2017)** | Longitudinal study shows that depression in childhood is associated with a worse evolution of headaches in adolescence | To see how headaches in children change over a five-year period as they become teenagers. The study also looked at what factors might predict if the headaches get better or worse. | 122 children who were treated for headaches at a clinic in Paris. At the start of the study, the average age was about 10 years. | This study did not have a control group. Instead, it followed the same group of children over time to see how their headaches evolved. | Revised Children's Manifest Anxiety Scale (RCMAS): A yes/no questionnaire to measure anxiety symptoms. Multiscore Depression Inventory for Children (MDIC): A yes/no questionnaire to measure symptoms of depression. Paediatric Migraine Disability Assessment (PedMIDAS): A questionnaire to measure how much migraines interfere with a child's daily life. |
| **Arruda et al. (2015)** | Psychosocial Adjustment of Children With Migraine and Tension-Type Headache—A Nationwide Study | To look at the emotional and behavioral traits of children with migraines and tension-type headaches (TTH), and to compare them with children who do not have headaches. | Group 1: Children with episodic migraine Group 2: Children with episodic tension-type headache (TTH) (The total sample was 5,671 children, aged 5-12 years) | A group of children from the same study who did not have headaches. | Headache Questionnaires: Validated questionnaires to diagnose different types of headaches based on the official ICHD-2 criteria. Strengths and Difficulties Questionnaire (SDQ): A widely used questionnaire, filled out by parents, to measure a child's emotional and behavioral problems across five key areas. |
| **Arruda et al. (2017)** | ADHD Is Comorbid to Migraine in Childhood: A Population-Based Study | To see if there is a connection between ADHD and different types of headaches in children, and to find out if the frequency of headaches makes a difference. | Group 1: Children with migraine Group 2: Children with tension-type headaches (The total sample was 5,671 children, aged 5-12 years) | A group of children from the same study who did not have headaches. | Headache Questionnaires: Validated questionnaires to diagnose different types of headaches. MTA-SNAP-IV Scale: A questionnaire to assess symptoms of ADHD. Strengths and Difficulties Questionnaire (SDQ): A questionnaire to measure a child's emotional and behavioral problems. |

**Table S1 (part 1 of 2).** Primary studies that met the inclusion criteria. *Note: Table S1 is presented in two parts; see part 2 for continuation.*

| | | | | | |
|---|---|---|---|---|---|
| **Attygalle et al. (2020)** | Migraine, attention deficit hyperactivity disorder and screen time in children attending a Sri Lankan tertiary care facility: are they associated? | To see if there is a connection between migraine and ADHD in children. The study also looked at how much time children with these conditions spend in front of screens. | 141 children, aged 5 to 14, who were being treated for migraines at a hospital in Sri Lanka. | 85 children of the same age who were at the same hospital for minor health issues but did not have migraines. | Swanson, Nolan, and Pelham (SNAP-IV) Questionnaire: A screening tool used to check for symptoms of ADHD. Clinical Interview: Children who scored high on the SNAP-IV questionnaire were then interviewed by a psychiatrist to confirm an ADHD diagnosis. |
| **Balottin et al. (2018)** | Rorschach Evaluation of Personality and Emotional Characteristics in Adolescents With Migraine Versus Epilepsy and Controls | To investigate the specific personality characteristics, emotion regulation, and coping strategies of adolescents with migraine, comparing them to adolescents with epilepsy and a healthy control group. | Group 1: 21 adolescents (age 11-17) diagnosed with migraine. Group 2: 20 adolescents (age 11-17) diagnosed with epilepsy (serving as a clinical control group). | 1 healthy adolescents (age 12-17) with no history of neurological or psychological issues. | Youth Self-Report (YSR) and Child Behavior Checklist (CBCL): Questionnaires for adolescents and their parents to report on emotional and behavioral symptoms. Rorschach Test (Exner Comprehensive System): A projective test using inkblots to assess deeper personality structures like emotion regulation, stress management, and thought processes. |
| **Basile, C. et al. (2023)** | Personality traits through psychological dimensions in adolescents with headache: an investigation on how social isolation during the pandemic influenced mental health—a pilot study | To see how the COVID-19 pandemic and the lockdown that followed affected the personality traits of teenagers with headaches. | 15 teenagers (aged 13-18) who were being treated for headaches at a clinic. | This study did not have a control group. Instead, it compared the same group of teenagers at two different times: before and after the lockdown. | Big Five Inventory (BFI): A questionnaire that measures five key personality traits: extraversion, agreeableness, conscientiousness, neuroticism, and openness. Child Behavior Checklist (CBCL): A questionnaire to check for mental health issues like anxiety and depression, somatic grievances, etc. |
| **Albanês Oliveira Bernardo et al. (2020)** | Osmophobia and Odor-Triggered Headaches in Children and Adolescents: Prevalence, Associated Factors, and Importance in the Diagnosis of Migraine | To investigate how common osmophobia (aversion to smells) and odor-triggered headaches are in children and to see how useful these symptoms are for diagnosing migraine. | Group 1: 253 children and adolescents (age 6-17) with migraine. Group 2: 47 children and adolescents (age 6-17) with tension-type headache. | There was no healthy control group; the tension-type headache group served as the main comparison. | Semi-structured questionnaire: To gather data on headache characteristics, osmophobia, and odor triggers. Wong-Baker FACES Pain Rating Scale: To measure headache intensity. Pediatric Migraine Disability Assessment (PedMIDAS): To assess how much headaches impact daily life. State-Trait Anxiety Inventory (STAI): To screen for anxiety. Children's Depression Inventory (CDI): To screen for depression. |

**Table S1 (part 1 of 2).** Primary studies that met the inclusion criteria. *Note: Table S1 is presented in two parts; see part 2 for continuation.*

| | | | | | |
|---|---|---|---|---|---|
| **Blaauw et al. (2015)** | The relationship of anxiety, depression and behavioral problems with recurrent headache in late adolescence—a Young-HUNT follow-up study | To see if symptoms of anxiety, depression, and behavioral problems in early adolescence could predict who would have recurrent headaches four years later. | Population-based cohort of Norwegian adolescents aged 12–16 at baseline (Young-HUNT1); n=1,586 with complete data; headache interview in Young-HUNT2 (n=1,665). | No experimental control group; comparisons between headache categories (migraine, TTH, unclassified) vs no headache; sub-analysis among those with no headache at baseline (n=882). | SCL-5 for anxiety/depression; school adjustment items for attention and conduct problems (mean score 1–4); nurse-conducted 'recognition-based' headache interview using IHS criteria; headache frequency in 4 categories (monthly, monthly, weekly, daily)." |
| **Bonuccelli et al. (2023)** | Essential headaches in developmental age: What is changed before, during and after the lockdown for COVID-19 pandemic. Clinical study | Evaluate variations in primary-headache frequency and medication use before, during, and after COVID-19 lockdown, and assess whether changes correlate with lifestyle modifications; secondarily, compare patterns between school-age children and adolescents. | N=90 patients (5–18 years) with primary headaches (migraine ± aura, tension-type headache) followed at the University Hospital of Pisa, Jan 2018–Mar 2022; 51.1% female; 56.7% adolescents; 77.7% onset before age 10; 68.9% family history. | None; within-subject comparisons across the three periods, with stratified analyses by age (5–11 vs 12–18) and sex. | Author-designed 21-item questionnaire covering demographics; headache type/frequency; medication use (acute, prophylaxis); psychotherapy; lifestyle/quality-of-life proxies (diet, hours of sleep, sleep problems, physical activity); screen time (1–3, 4–6, 7–9, >10 h/day). |
| **Canfora et al. (2023)** | More Than a Headache: Lived Experience of Migraine in Youth | To understand and describe the real-life impact of migraine on children, adolescents, and their caregivers through their own words and experiences. | This was a qualitative study, so there was not an experimental group in the usual sense. The participants were 30 children and adolescents (median age 15) with migraine, along with 29 of their caregivers. | There was no control group in this study | Semi-structured interviews: The main method was in-depth interviews with both the youth and their caregivers to explore their experiences with migraine. Pediatric Migraine Disability Assessment Score (PedMIDAS): This was used to obtain a baseline measure of headache disability. |
| **Chiappedi et al. (2018)** | Intellectual Profile of Adolescents with Headache: A Case-Control Study Using the WISC-IV | To see if there are differences in the intellectual profiles of teenagers with primary headaches compared to teenagers without headaches. | 30 adolescents (aged 11-14) who were being treated for headaches at a clinic. This group included 16 teenagers with migraine and 14 with tension-type headache. | 30 healthy teenagers of the same age and sex who did not have frequent headaches. | Wechsler Intelligence Scale for Children—Fourth Edition (WISC-IV): A comprehensive IQ test that measures different areas of cognitive ability, including verbal comprehension, perceptual reasoning, working memory, and processing speed. |

**Table S1 (part 1 of 2).** Primary studies that met the inclusion criteria. *Note: Table S1 is presented in two parts; see part 2 for continuation.*

| | | | | | |
|---|---|---|---|---|---|
| **Clementi et al. (2023)** | Preliminary Exploration of a Multidimensional Sleep Health Composite in Adolescent Females with Frequent Migraine | To describe a new, multidimensional sleep health score for adolescent females with frequent migraines and to see how this overall score relates to their headaches and emotional health. | 60 female adolescents (aged 12-18) who had a diagnosis of high-frequency episodic or chronic migraine. | No control group | Actigraphy: A wrist-worn device that objectively measured sleep patterns over 10 days. Daily Electronic Surveys: Used to track daily headache details, sleep quality, anxiety, and sadness. Adolescent Insomnia Questionnaire (AIQ) and Sleep Disorders Inventory for Students-Revised-Adolescent (SDIS-R-A): Questionnaires to assess sleep problems and insomnia. Pediatric Migraine Disability Assessment (PedMIDAS): A questionnaire to measure how much migraines interfere with daily life. |
| **Colon et al. (2019)** | Migraine in the Young Brain: Adolescents vs. Young Adults | To evaluate age-related brain changes in resting-state functional connectivity (rs-FC) in patients with migraine compared to healthy controls, contrasting two developmental stages: adolescence and young adulthood. | Group 1: 18 adolescents (age 12-18) diagnosed with episodic migraine. Group 2: 18 young adults (age 19-27) diagnosed with episodic migraine. | Group 1: 18 healthy adolescents, matched for age and sex. Group 2: 18 healthy young adults, matched for age and sex. | Resting-state functional Magnetic Resonance Imaging (rs-fMRI): Utilized to measure the brain's resting-state functional connectivity (rs-FC) and to analyze Resting-State Networks (RSNs). Headache Questionnaires: Employed to collect clinical data on migraine characteristics such as age of onset, attack frequency, and duration. |
| **Costa-Silva et al. (2016)** | Cognitive functioning in adolescents with migraine | To investigate the cognitive performance of adolescents diagnosed with migraine by comparing them to a control group of individuals without a history of headache. | 28 adolescents (age 10-18) with a clinical diagnosis of migraine. | 26 healthy adolescents (age 10-18) with no history of headache, matched for socioeconomic status. | Rey Auditory Verbal Learning Test (RAVLT): To evaluate episodic memory and verbal learning. Trail-Making Test (TMT A and B): To evaluate executive functions, including attention and processing speed. Stroop Test: To assess executive functions, particularly selective attention and inhibitory control. Verbal Fluency Test (VFT): To assess language abilities. |

**Table S1 (part 1 of 2).** Primary studies that met the inclusion criteria. *Note: Table S1 is presented in two parts; see part 2 for continuation.*

| | | | | | |
|---|---|---|---|---|---|
| **Armony Domany et al. (2019)** | Clinical presentation, diagnosis and polysomnographic findings in children with migraine referred to sleep clinics | To describe the clinical presentation, polysomnographic (PSG) characteristics, and comorbid sleep disorder diagnoses in children with migraine who had been referred for evaluation at a sleep clinic. | 185 children (≤18 years of age) with a diagnosis of migraine who had been evaluated at both a headache center and a sleep center, where they underwent polysomnography. | 180 healthy children (age 5-14) from a research registry who had undergone polysomnography and were confirmed to have no significant sleep-disordered breathing. | Polysomnography (PSG): An objective, overnight sleep study used to measure sleep architecture (sleep stages), respiratory events, and limb movements. Pediatric Migraine Disability Assessment (PedMIDAS): A questionnaire to measure the functional disability caused by migraine. Retrospective Chart Review: Used to collect data on headache characteristics, clinical sleep diagnoses, and presenting sleep complaints. |
| **Donnelly et al. (2017)** | Common pediatric pain disorders and their clinical associations | To investigate the associations between common childhood pain conditions (e.g., migraine, growing pains), other persistent pains, and selected non-pain conditions (restless legs syndrome, iron deficiency, anxiety/depression). | Cohort of 2,530 pediatric individuals (age 3-18), including twins and their siblings. Case groups were defined by the presence of specific conditions, including:<br>- Growing Pains: 473 individuals (19%);<br>- Non-migraine Headache: 215 individuals (~14%);<br>- Migraine: 169 individuals (~7%);<br>- Recurrent Abdominal Pain: 294 individuals (~12%);<br>- Persistent Pain: 155 individuals;<br>- Low Back Pain: 176 individuals (~13%). | The control group for each analysis consisted of individuals from the same cohort who did not meet the criteria for the specific condition being investigated. | A series of parent- and child-completed questionnaires were used to assess the lifetime prevalence of various conditions. Diagnoses were based on established criteria (e.g., ICHD-II for migraine/headache, ROME III for a subset with abdominal pain). Psychological symptoms were assessed with the Anxious/Depressed syndrome subscale of the Achenbach System of Empirically Based Assessment (ASEBA) Child Behavior Checklist (CBCL). |
| **Finning et al. (2022)** | Mental health and school absenteeism in children with long-term physical conditions: A secondary analysis of the British Child and Adolescent Mental Health Surveys 2004 and 2007 | To investigate the short-term (cross-sectional) and long-term (longitudinal) associations between various long-term physical conditions (LTCs) and both mental health and school absenteeism in children and young people. | The study analyzed a large, nationally representative sample from a survey. The primary group of interest consisted of 2,341 children and young people (CYP) with at least one long-term physical condition, including subgroups for specific conditions like migraine/severe headaches, atopic conditions, and neurodevelopmental disorders. | 5,249 children and young people from the same survey who did not have any of the specified long-term physical conditions. | Strengths and Difficulties Questionnaire (SDQ): A parent-reported questionnaire used to screen for and measure the severity of psychopathology (emotional and behavioral problems). Development and Wellbeing Assessment (DAWBA): A diagnostic interview used to determine the presence of any formal psychiatric disorders. Parent-reported absenteeism: Parents reported the number of school days their child missed in the previous term. |

**Table S1 (part 1 of 2).** Primary studies that met the inclusion criteria. *Note: Table S1 is presented in two parts; see part 2 for continuation.*

| | | | | | |
|---|---|---|---|---|---|
| **Gatta et al. (2015)** | Alexithymic characteristics in pediatric patients with primary headache: a comparison between migraine and tension-type headache | To determine the prevalence of alexithymia in children and adolescents with either migraine or TTH, and to investigate the correlation between alexithymia in the children and their mothers. | Group 1: 47 children and adolescents (age 8-17) diagnosed with TTH. Group 2: 42 children and adolescents (age 8-17) diagnosed with migraine (M). | 32 healthy children and adolescents (age 8-17) with no history of headache. | Alexithymia Questionnaire for Children (AQC): A self-report questionnaire administered to the pediatric participants to measure alexithymic traits. Toronto Alexithymia Scale (TAS-20): A self-report questionnaire administered to the mothers of all participants to measure their own levels of alexithymia. |
| **Genizi et al. (2016)** | Pediatric mixed headache—The relationship between migraine, tension-type headache and learning disabilities—in a clinic-based sample | To assess the prevalence and characteristics of children who experience both migraine and tension-type headache (TTH), referred to as "mixed headaches," and to investigate the association between these headaches and learning disabilities. | 262 children and adolescents (age 5-18) who were all diagnosed with migraine. For the main analysis, the authors compared two subgroups from this cohort:<br>- Mixed Headache Group: 59 children who had both migraine and co-occurring episodic TTH .<br>- Migraine Alone Group: 182 children who had migraine but did not have co-occurring TTH. | This study did not include a healthy control group without headaches. The primary comparison was between different subgroups of children with migraine. | Semi-structured interviews, questionnaires, and headache diaries: Based on the International Classification of Headache Disorders (ICHD-II) criteria, these tools were used to diagnose headache types and gather clinical information. Formal Psycho-educational Assessment: Administered to children who reported learning difficulties to provide a formal diagnosis of a learning disability. |
| **Genizi et al. (2020)** | Sensory processing patterns affect headache severity among adolescents with migraine | To evaluate the relationship between pain catastrophizing, sensory processing patterns, and headache severity in adolescents with episodic migraine compared to healthy controls. | 40 adolescents (age 13-18) with a clinical diagnosis of episodic migraine. | 52 healthy adolescents (age 13-18) with no significant illness or history of migraine. | Adolescent/Adult Sensory Profile (AASP): A self-report questionnaire that measures behavioral responses to sensory input in daily life across four patterns: Sensation Seeking, Sensation Avoiding, Sensory Sensitivity, and Low Registration. Pain Catastrophizing Scale for Children (PCS-C): A questionnaire to assess catastrophic thinking related to pain, yielding a total score and three subscale scores: Rumination, Magnification, and Helplessness. Pediatric Migraine Disability Assessment (PedMIDAS): A questionnaire used to measure the level of headache-related disability. |

**Table S1 (part 1 of 2).** Primary studies that met the inclusion criteria. *Note: Table S1 is presented in two parts; see part 2 for continuation.*

| | | | | | |
|---|---|---|---|---|---|
| **Genizi et al. (2021)** | Migraine and Tension-Type Headache Among Children and Adolescents: Application of International Headache Society Criteria in a Clinical Setting | To assess the clinical applicability and effectiveness of the International Headache Society (IHS) diagnostic criteria for primary headaches in a real-world pediatric neurology clinic setting. | This was a retrospective study of a large clinical cohort. The main groups identified according to IHS criteria were as follows: Group 1: 256 children and adolescents (age 6-18) diagnosed with migraine. Group 2: 241 children and adolescents (age 6-18) diagnosed with tension-type headache (TTH). Group 3: 45 children and adolescents (age 6-18) diagnosed with mixed headache. | There was no healthy control group. The study focused on the diagnostic distribution and characteristics of patients presenting with headache. | The study was based on a retrospective review of medical records. Diagnoses were made by clinicians based on unstructured interviews guided by the International Classification of Headache Disorders (ICHD-3) criteria. No standardized psychological tests were administered as part of this study. |
| **Genizi et al. (2025)** | Executive Functions, Anxiety, Social Participation and Quality of Life in Children with Migraine During COVID-19 | To compare executive functions (EF), anxiety, social participation, and quality of life (QoL) between children with migraine and healthy controls during the COVID-19 pandemic, and to examine if these factors differed based on whether the child had contracted COVID-19. | 33 children and adolescents (age 6-17.5) with a clinical diagnosis of episodic migraine. | 51 healthy children and adolescents of a similar age with no neurological or developmental disorders. | Behavior Rating Inventory of Executive Functions (BRIEF): A parent-report questionnaire to measure executive functions as expressed in daily life, including behavioral regulation and metacognition. State-Trait Anxiety Inventory for Children (STAIC): A questionnaire to measure both immediate (state) and general (trait) anxiety. Child and Adolescent Scale of Participation (CASP): A questionnaire to measure participation in home, school, and community activities. Pediatric Quality of Life Inventory (PedsQL): A self-report questionnaire to assess health-related quality of life across physical, emotional, social, and school domains. |
| **Gerstl et al. (2021)** | Migraine and the development of additional psychiatric and pain disorders in the transition from adolescence to adulthood | To investigate whether adolescents diagnosed with migraine have a higher risk of developing additional psychiatric disorders or other pain syndromes over the next 10 years compared to adolescents with no history of headache. | 431 adolescents who were 15 years old in 2006 and had a confirmed diagnosis of migraine based on health insurance data. | 22,368 adolescents from the same health insurance database who were also 15 years old in 2006 but had no headache diagnoses recorded that year. | This study did not use psychometric tests. It was a retrospective analysis of a large health insurance database. International Classification of Diseases (ICD-10) codes: Used to identify individuals with migraine and to track the emergence of new psychiatric and pain disorder diagnoses over a 10-year period. |

**Table S1 (part 1 of 2).** Primary studies that met the inclusion criteria. *Note: Table S1 is presented in two parts; see part 2 for continuation.*

| | | | | | |
|---|---|---|---|---|---|
| **Gibler et al. (2024)** | Clinic-Based Characterization of Adolescents and Young Adults With Migraine: Psychological Functioning, Headache Days, and Disability | To characterize the headache features and psychological functioning of adolescents and young adults (AYAs) seeking care at a specialty headache clinic, and to examine the association between anxiety/depression symptoms and headache burden. | 283 adolescents and young adults (age 14-32) who presented for care at a tertiary headache clinic. | There was no control group in this study. | Headache Intake Questionnaire: To gather data on headache frequency, severity, duration of symptoms, and history of psychiatric diagnoses. Generalized Anxiety Disorder Scale-7 Item (GAD-7): A brief, validated screening tool to assess the severity of anxiety symptoms. Patient Health Questionnaire-9 (PHQ-9): A brief, validated screening tool to assess the severity of depressive symptoms, including an item on suicidal ideation. Pediatric Migraine Disability Assessment (PedMIDAS) and Migraine Disability Assessment (MIDAS): Age-appropriate questionnaires to measure the degree of headache-related functional disability. |
| **Giricz et al. (2021)** | Visually guided associative learning in pediatric and adult migraine without aura | To investigate if the deficits in visually guided associative learning previously found in adults with migraine are also present in children and adolescents with the same condition. | Group 1: 27 children and adolescents (mean age 14.1 ±2.9 years) newly diagnosed with migraine without aura. The study also re-analyzed data from a previous study for comparison: Group 2: 22 adults (mean age 40±11.76 years) with a history of migraine without aura. | Group 1: 27 healthy children and adolescents, matched for age, sex, and intelligence. Group 2 (from previous study): 22 healthy adults, matched for age and sex. | Rutgers Acquired Equivalence Test (RAET): A computer-based paradigm designed to measure a specific type of associative learning. It involves an "acquisition phase" (learning face-fish pairs with feedback) and a "test phase" (recalling learned pairs and generalizing the rule to new pairs without feedback). Raven's Progressive Matrices: Used to assess intelligence for matching pediatric participants. |
| **Goenka & Fonseca (2023)** | Rate and Predictors of Intractable Status Migrainosus among Patients Aged 13-18 Years | To assess clinical factors associated with intractable (treatment-refractory) status migrainosus in adolescents who required inpatient hospital care. | The study analyzed a cohort of 159 adolescents (age 13-18) who presented with status migrainosus and required an aggressive, three-tiered treatment protocol. This cohort was divided for analysis into: Group 1 (Non-responders): 34 patients whose pain did not improve by at least 50% after all three lines of treatment. Group 2 (Responders): 125 patients whose pain did improve by at least 50%. | There was no healthy control group. The study's comparison was between patients who responded to intensive treatment and those who did not. | Visual Analogue Scale (VAS): An 11-point scale used to measure self-reported pain intensity at admission and discharge. This was the primary outcome measure. Generalized Anxiety Disorder 7-item scale (GAD-7): A self-report scale used to assess the severity of anxiety symptoms in patients with a known comorbidity of anxiety. |

**Table S1 (part 1 of 2).** Primary studies that met the inclusion criteria. *Note: Table S1 is presented in two parts; see part 2 for continuation.*

| | | | | | |
|---|---|---|---|---|---|
| **Güler et al. (2017)** | The High Level of Psychiatric Disorders Associated with Migraine or Tension-type Headache in Adolescents | To evaluate the relationship between psychiatric disorders (and the severity of anxiety/depression symptoms) and two types of primary headaches—migraine and tension-type headache (TTH)—in an adolescent clinical population. | The study analyzed a cohort of 140 adolescents (age 12-18) with primary headaches, who were divided into two main groups: Group 1: 98 adolescents diagnosed with migraine. Group 2: 42 adolescents diagnosed with tension-type headache (TTH). | This study did not include a healthy control group without headaches. The primary comparison was between the migraine and TTH groups. | Face-to-face interviews: Conducted by a headache specialist to diagnose headache type according to the International Classification of Headache Disorders, 3rd edition beta version (ICHD-3 beta). Psychiatric assessment: Based on the Diagnostic and Statistical Manual of Mental Disorders, 4th Edition (DSM-IV) criteria, to diagnose comorbid psychiatric disorders. State-Trait Anxiety Scale for Children (STAI-C): A self-report questionnaire to measure both immediate (state) and general (trait) anxiety. Depression Scale for Children (DSC): A self-report questionnaire to measure the severity of depressive symptoms. |
| **Hartberg et al. (2015)** | Coping strategies among adolescents with chronic headache and mental health problems: a cross-sectional population-based study | To determine the prevalence of mental health problems in adolescents with chronic headache and to compare the coping strategies used by adolescents with chronic headache who do and do not have co-occurring mental health problems. | This was a large cross-sectional survey. For the analysis, participants were categorized into three main groups of interest: Group 1: 717 adolescents with chronic headache but no significant mental health problems (CH). Group 2: 212 adolescents with both chronic headache and mental health problems (CHMH). Group 3: 1,049 adolescents with mental health problems but no chronic headache (MH). | 17,143 adolescents from the same survey who had neither chronic headache nor significant mental health problems. | Single-item headache question: A question asking about headache frequency over the past 6 months was used to identify adolescents with chronic headache (defined as "almost every day"). Strengths and Difficulties Questionnaire (SDQ): A validated questionnaire used to assess mental health problems across four domains (emotional, conduct, hyperactivity, peer problems) and their impact on daily life. Custom Coping Questionnaire: A seven-item questionnaire asking how participants respond to "painful thoughts and feelings," with items categorized as either internal or external coping strategies. |
| **de Oliveira-Souza et al. (2022)** | The presence of migraine symptoms was associated with a higher likelihood to present eating disorders symptoms among teenage students | To investigate the association between migraine and the presence of signs and symptoms of eating disorders in a sample of adolescents. | 454 adolescents (age 11-18) with a diagnosis of migraine, which also included probable migraine and migraine combined with tension-type headache. | 153 adolescents (age 11-18) without migraine, a category that included those with tension-type headache, non-classifiable headache, or no headache at all. | Eating Attitudes Test-26 (EAT-26): A self-report questionnaire used to screen for symptoms of eating disorders. Bulimic Investigatory Test of Edinburgh (BITE): A self-report questionnaire used to identify bulimic episodes and related cognitive and behavioral factors. Headache Questionnaire: Based on the |

**Table S1 (part 1 of 2).** Primary studies that met the inclusion criteria. *Note: Table S1 is presented in two parts; see part 2 for continuation.*

| | | | | | |
|---|---|---|---|---|---|
| | | | | | International Classification of Headache Disorders (ICHD-II) criteria to identify and characterize headaches. |
| **Cerutti et al. (2016)** | Alexithymia and psychopathological symptoms in adolescent outpatients and mothers suffering from migraines: a case control study | To explore the relationship between migraine and alexithymia in a group of adolescents and their mothers, and to verify whether alexithymia can be a predictor of psychopathological symptoms. | 53 adolescents (mean age 13.39) with a diagnosis of migraine and their 53 mothers. | 53 healthy adolescents (mean age 12.51) and their 53 mothers, without a history of migraine. | Headache Questionnaire used to investigate the presence or absence of migraine according to the ICHD-3 beta criteria. Toronto Alexithymia Scale (TAS-20): A questionnaire to assess alexithymic traits. Symptom Checklist-90-R (SCL-90-R): A questionnaire to assess a wide range of psychopathological symptoms (e.g., somatization, depression, anxiety). |
| **Esposito et al. (2021)** | Neuropsychological Profile in Pediatric Migraine without Aura: A Pilot Study | To assess the neuropsychological skills of children affected by migraine without aura (MwoA). | 15 children (7 male, 8 female) with a mean age of 10.73±2,13), diagnosed with MwoA. | 38 typically developing children, comparable to the MwoA group in age, gender, language, and education level. | NEPSY-II: A comprehensive battery of neuropsychological tests used to assess skills across six domains: Attention and Executive Functions, Language, Memory and Learning, Sensorimotor Functions, Social Perception, and Visuospatial Processing. Wechsler Intelligence Scale for Children—Third Edition (WISC-III): Used to evaluate cognitive level and exclude intellectual disabilities. Visual Analogue Scale (VAS) and Pediatric Migraine Disability Assessment (PedMIDAS): Used to assess pain level and the degree of disability. |

**Table S1 (part 1 of 2).** Primary studies that met the inclusion criteria. *Note: Table S1 is presented in two parts; see part 2 for continuation.*

| | | | | | |
|---|---|---|---|---|---|
| **Fielding et al. (2016)** | Headache symptoms consistent with migraine and tension-type headaches in children with anxiety disorders | To examine the incidence of headache symptoms consistent with migraine and tension-type headache (TTH) in children with anxiety disorders. | 27 children with anxiety disorders (mean age 8.59±1.97 years). | 36 children without anxiety disorders (mean age 7.92±2.14 years). | Anxiety Diagnosis: Anxiety Disorders Interview Schedule: Child/Parent Versions (ADIS-IV-C/P). Anxiety Symptom Severity: Spence Children's Anxiety Scale, Parent and Child Versions (SCAS-P and SCAS-C). Headache Symptoms: A parent-report headache questionnaire developed by the researchers based on the International Classification of Headache Disorders (ICHD-II) criteria. |
| **Hommer et al. (2022)** | Headache and mental disorders in a nationally representative sample of American youth. | To examine the association between headache subtypes (migraine and non-migraine) and DSM-IV mental disorders in a large, nationally representative sample of American adolescents (ages 13-18). | 2,711 adolescents (ages 13-18) with a lifetime history of headache, subdivided into Migraine with aura (n=171), Migraine without aura (n=1,074), and Non-migraine headache (n=1,466). | 7,412 adolescents (ages 13-18) with no history of headache. | Data Source: National Comorbidity Survey-Adolescent Supplement (NCS-A). Assessment Interview: A modified version of the World Health Organization Composite International Diagnostic Interview (WHO-CIDI). Diagnostic Criteria: Headache subtypes were classified using modified ICHD-3 criteria; mental disorders were classified using DSM-IV criteria. |
| **Hsu et al. (2022)** | Attention deficit hyperactivity disorder and risk of migraine: A nationwide longitudinal study | To explore the longitudinal risk of developing migraine in children, adolescents, and young adults with ADHD and to assess if this risk is associated with ADHD medications. | 81,441 participants with ADHD (mean age 9.0 ± 4.1 years), of whom 71.8% were males. The cohort was analyzed across three age groups at enrollment: children (3-11 years), adolescents (12-17 years), and young adults (18-29 years). | 81,441 participants without ADHD (mean age 9.0 ± 4.1 years). This group was matched 1:1 to the ADHD group for age, sex, and specific physical/psychiatric comorbidities at the time of enrollment. | Data Source: Taiwan National Health Insurance Research Database (NHIRD). Diagnostic Codes: Diagnoses of ADHD and migraine were identified using International Classification of Diseases, Ninth Revision, Clinical Modification (ICD-9-CM) codes from the database. |
| **Jafari et al. (2023)** | The influence of anxiety and depression on headache in adolescent migraineurs: a case-control study | To compare the frequency of anxiety and depression in adolescents with and without migraine, and to evaluate how anxiety and depression relate to specific migraine characteristics like attack frequency and severity. | 112 adolescents with migraine (mean age 15.77 ± 2 years). | 122 non-migraine adolescents (mean age 15.39 ± 1.79 years). | Headache Assessment: A headache questionnaire and diary were used. Migraine diagnosis was confirmed by neurologists using the International Classification of Headache Disorders-3 (ICHD3) criteria. Headache severity was measured with a Numeric Rating Scale (NRS). Anxiety Assessment: Beck Anxiety Inventory (BAI). Depression Assessment: Children's Depression Inventory (CDI). |

**Table S1 (part 1 of 2).** Primary studies that met the inclusion criteria. *Note: Table S1 is presented in two parts; see part 2 for continuation.*

| | | | | | |
|---|---|---|---|---|---|
| **Kemper et al. (2016)** | Factors Contribute to Headache-Related Disability in Teens? | To describe the relationship between risk factors (stress, depression, anxiety), protective factors (mindfulness, self-compassion, resilience), and headache-related disability in adolescents. A secondary aim was to assess teens' interest in mind-body skills training for headache management. | 29 adolescents (mean age 14.8 ± 2 years) | This study was a single-cohort descriptive study and did not include a control group. | Headache Disability: Headache Impact Test (HIT-6). <u>Risk Factors</u>: - Stress: Cohen's 10-item Perceived Stress Scale. - Anxiety & Depression: PROMIS Pediatric Anxiety and Depression Scales. - Sleep Disturbance: PROMIS Sleep Disturbance Scale. <u>Protective Factors</u>: - Mindfulness: Cognitive and Affective Mindfulness Scale, Revised (CAMS-R). - Self-Compassion: Neff's Self-Compassion Scale (short form). - Resilience: Smith's 6-item Brief Resilience Scale. <u>Physiological Measures:</u> Heart Rate Variability (HRV) and blood biomarkers of inflammation (hsCRP and IL-6). |
| **Lateef et al. (2019)** | Headaches and sleep problems in US adolescents: Findings from the National Comorbidity Survey—Adolescent Supplement (NCS-A) | To examine the association between different headache subtypes (e.g., migraine with/without aura) and various sleep problems in a large, nationally representative sample of US adolescents. | 104 adolescents with migraine with aura (ages 13-18). 578 adolescents with migraine without aura (ages 13-18). | 586 adolescents with other non-migraine headache (ages 13-18). | Data Source: The National Comorbidity Survey Adolescent Supplement (NCS-A). Headache Assessment: Headache subtypes were classified using modified ICHD-III criteria based on survey questions. Sleep Assessment: Self-reported sleep duration, bedtime, and specific insomnia symptoms. Psychological Assessment: Mood and anxiety disorders were diagnosed based on the World Health Organization Composite International Diagnostic Interview (CIDI). |

**Table S1 (part 1 of 2).** Primary studies that met the inclusion criteria. *Note: Table S1 is presented in two parts; see part 2 for continuation.*

| | | | | | |
|---|---|---|---|---|---|
| **Law et al. (2019)** | Screening Family and Psychosocial Risk in Pediatric Migraine and Tension-Type Headache: Validation of the Psychosocial Assessment Tool (PAT) | To evaluate the reliability and validity of the Psychosocial Assessment Tool (PAT) as a brief screening instrument for identifying family and psychosocial risk in youth with recurrent headaches. | 157 participants from a neurology clinic (14 years, 9 months (SD 1.85)). 82 participants from the community (14 years, 3 months (SD 1.89)). | This was a validation study and did not include a healthy control group. | At baseline, children self-reported on headache related disability using the Pediatric Migraine Disability Assessment Scale (PedMIDAS). Primary Tool (being validated): Psychosocial Assessment Tool (PAT), a parent-report questionnaire designed to screen for psychosocial risk. Validation Measures: Established questionnaires assessing child emotional/behavioral functioning (CBCL), parent anxiety (STAIS), parent depression (BDI), and family functioning (FAD). |
| **Lee, S.M. et al. (2015)** | Screening for depression and anxiety disorder in children with headache | To investigate the importance of initial screening tests for depression and anxiety disorders in children with headache. To evaluate whether the Children's Depression Inventory (CDI) and Revised Children's Manifest Anxiety Scale (RCMAS) are suitable for screening symptoms of depression and anxiety. | 720 children aged 7–17 years (mean age 11.7 years). | This study did not include a control group. | Headache Assessment: A structured headache questionnaire and an 11-point pain scale. Depression Screening: Children's Depression Inventory (CDI). Anxiety Screening: Revised Children's Manifest Anxiety Scale (RCMAS). |
| **Lee, K.M. et al. (2022)** | Assessment of Parenting Attitudes by Children and Adolescents with Migraine | To investigate the parenting attitudes reported by children and adolescents with migraine and to examine the relationship between these perceived attitudes and headache characteristics. | 62 elementary-school children (mean age 10.32±1.30 years). 53 middle-school-age children (mean age 13.72±0.66 years). | This study did not include a control group. | Parenting Attitudes: Parenting Attitude Test-Youth (PAT-Y), a self-report scale completed by the child to assess their perception of parental attitudes. Headache Assessment: Visual Analog Scale (VAS) for severity and Monthly Frequency (MF) for the number of headache days. Psychological Assessment: Children's Depression Inventory (CDI), Child Behavior Checklists (CBCL), and an ADHD rating scale. |
| **Magnerou et al. (2024)** | Prevalence and impact of primary headaches among students aged 8–12 years in Sub-Saharan Africa: Cameroon experience | To investigate the prevalence and impact of primary headaches (migraine and tension-type headache) among primary school students aged 8-12 years in Douala, Cameroon. | 2,056 total students surveyed, 1,762 (85.7%) reported having a headache in the last 12 months. These 1,762 students with headaches were categorized as follows: 536 students with migraine, 311 students with tension-type headache (TTH), and 916 students with "Other headaches" (headaches that did not meet the criteria for migraine or TTH). | 294 students (2,056 total - 1,762 with headache) constituted the headache-free control group. | Headache Assessment: The pediatric version of the HARDSHIP questionnaire, which is based on the International Classification of Headache Disorders, 3rd ed (ICHD-3) criteria. Pain Assessment: Visual Analogue Scale (VAS) to measure pain intensity. |

**Table S1 (part 1 of 2).** Primary studies that met the inclusion criteria. *Note: Table S1 is presented in two parts; see part 2 for continuation.*

| | | | | | |
|---|---|---|---|---|---|
| **Maltese et al. (2017)** | Internalizing problems are related to sleep patterns disordered in children affected by primary headache | To investigate the relationship between psychological problems and sleep disturbances in children with primary headaches. | 64 subjects aged 8-12 years (mean age 9.4±1.03 years). headache subtype The distribution was as follows: migraine without aura (71.87%), episodic tension-type headache (7.81%), and chronic tension-type headache (7.81%). | This study did not include a control group. | Psychological Assessment: Child Behavior Checklist (CBCL), completed by parents to measure emotional and behavioral problems. Sleep Assessment: Sleep Disturbances Scale for Children (SDSC), completed by parents to identify sleep disorders. Headache Diagnosis: International Classification of Headache Disorders, 3rd Edition (ICHD-III) criteria. |
| **Margari et al. (2018)** | Non-Verbal Cognitive Abilities in Children and Adolescents Affected by Migraine and Tension-Type Headache: An Observational Study Using the Leiter-3 | To assess non-verbal cognitive skills, particularly memory and attention, in children and adolescents with primary headaches and to see how these skills relate to headache characteristics. | 35 children and adolescents (mean age 14.89±3.2 years) with a primary headache diagnosis. This group included patients with migraine (n=26) and tension-type headache (n=9). | 23 healthy children and adolescents (mean age 14.75±3.17 years), matched for age and sex, served as the control group. | Cognitive Assessment: Leiter International Performance Scale Third Edition (Leiter-3), a comprehensive non-verbal test of intelligence, attention, and memory. Headache Assessment: Pediatric Migraine Disability Assessment Score (PedMIDAS) to measure disability, and a questionnaire for headache frequency and duration. |
| **Öksüz et al. (2024)** | Internalizing disorders rather than ADHD are risk factors for chronicity in pediatric migraine patients | To compare the migraine characteristics of pediatric patients with comorbid internalizing disorders (e.g., depression, anxiety) versus those with comorbid externalizing disorders (e.g., ADHD) to identify risk factors for migraine chronification. | 173 patients with comorbid internalizing disorders (depression and/or anxiety) (mean age 14.52 ± 2.82 years). 107 patients with comorbid externalizing disorders (primarily ADHD) (mean age 13.2 ± 2.79 years). | The study's design was a comparison between two different clinical groups. | Data Source: Turkish headache database, Mersin Branch. Headache Assessment: Retrospective evaluation of pain characteristics (frequency, intensity via VAS) and migraine subtype based on ICHD-3 criteria. Psychological Assessment: Psychiatric diagnoses were made by a child and adolescent psychiatrist according to DSM-IV criteria. |
| **Nahman-Averbuch et al. (2022)** | New insight into the neural mechanisms of migraine in adolescents: Relationships with sleep | To examine if subjective and objective sleep difficulties could explain differences in brain functioning between adolescents with migraine and healthy controls.. | 20 adolescents with migraine (mean age 14.7±1.8 years). | 20 healthy adolescents (mean age 14.8±1.6 years). | Headache & Disability: Functional Disability Inventory (FDI), Pediatric Migraine Disability Assessment Scale (PedMIDAS). Subjective Sleep: Insomnia Severity Index (ISI) and Adolescent Sleep Hygiene Scale (ASHS). Objective Sleep: Wrist-worn actigraphy to measure sleep patterns (e.g., wake minutes after sleep onset [WASO]). Other: fMRI to assess brain connectivity (amygdala). |

**Table S1 (part 1 of 2).** Primary studies that met the inclusion criteria. *Note: Table S1 is presented in two parts; see part 2 for continuation.*

| | | | | | |
|---|---|---|---|---|---|
| **Natalucci, Faedda, Quinzi, Fegatelli, Vestri, et al. (2019)** | Alexithymia, Metacognition, and Theory of Mind in Children and Preadolescents With Migraine Without Aura (MWoA): A Case-Control Study | To compare levels of alexithymia, metacognition, and Theory of Mind (ToM) in children and preadolescents with migraine without aura versus a healthy control group. | 70 children and preadolescents (mean age 10.18±1.55 years) with a diagnosis of Migraine Without Aura. | 70 healthy children and preadolescents (mean age 10.57±1.66 years), matched for age and gender. | Alexithymia: Alexithymia Questionnaire for Children (AQC). Metacognition: Io e la Mia Mente (IeLMM) for ages 8-10 and Metacognitions Questionnaire for Children (MCQ-C) for ages 11-13. Theory of Mind (ToM): Social Perception subtests from the NEPSY-II neuropsychological battery. |
| **Natalucci, Faedda, Quinzi, Fegatelli, Fazi et al. (2019)** | Metacognition and theory of mind in children with migraine and children with internalizing disorders | To compare levels of alexithymia, metacognition, and Theory of Mind (ToM) between pre-adolescents with migraine without aura (MWoA) and a clinical group of children with internalizing disorders (anxiety or depression). | 34 pre-adolescents with Migraine Without Aura (MWoA) (mean age 11 years). 32 children and adolescents with an internalizing disorder (anxiety or depression) (mean age 10 years). | This study compared two different clinical populations and did not include a healthy control group. | Alexithymia: Alexithymia Questionnaire for Children (AQC). Metacognition: Metacognitions Questionnaire for Children (MCQ-C). Theory of Mind (ToM): Social Perception subtests from the NEPSY-II battery. Diagnoses: ICHD-3 beta criteria for migraine and ICD-10 for internalizing disorders. |
| **Nita et al. (2020)** | The Role of Polysomnography in Identifying Sleep Disorders in Children with Migraine | To evaluate the correlation between migraine and sleep disorders in children by objectively analyzing their sleep architecture with polysomnography (PSG). | 18 children and adolescents (age 5-17 years; mean age 12) with a diagnosis of migraine. | 16 age-matched children and adolescents (mean age 12.3 years) who had non-migraine headaches and were diagnosed with emotional disorders. | Sleep Assessment: In-patient, full-night polysomnography (PSG) to objectively measure sleep architecture (e.g., sleep stages, arousals, sleep latency). Headache Diagnosis: International Classification of Headache Disorders, 3rd edition (beta version) criteria. |
| **Operto et al. (2018)** | Parenting stress and emotional/Behavioral Problems in adolescents with Primary headache | To investigate parenting stress and emotional/behavioral problems in adolescents with primary headache and compare them to a healthy control group. | 35 adolescents (mean age 14.89±3.2 years) with a diagnosis of primary headache. | 23 healthy adolescents (mean age14.75±3.17 years), matched for age and sex. | Parenting Stress: Parent Stress Index-Short Form (PSI-SF), a parent-report questionnaire. Child Psychological Assessment: Child Behavior Checklist (CBCL), a parent-report measure of their child's emotional and behavioral problems. Headache Disability: Pediatric Migraine Disability Assessment Score (PedMIDAS). |
| **Operto et al. (2022)** | Migraine and epilepsy: Social cognition skills in pediatric population | To compare social cognition skills (Theory of Mind and Emotion Recognition) in children and adolescents with migraine, focal epilepsy, and typically developing peers, and to relate these skills to executive functions. | 57 children and adolescents with migraine (mean age 11.44±2.26 years). 62 children and adolescents with focal epilepsy (mean age 12.74±3.4 years). | 61 typically developing children and adolescents (mean age 11.56±3.28 years). | Social Cognition: The NEPSY-II battery to assess Theory of Mind (TM) and Emotion Recognition (ER). Executive Functions: EpiTrack Junior. Non-verbal Intelligence: Raven's Progressive Matrices (RPM). Diagnoses: ICHD-III beta for migraine and ILAE 2017 for epilepsy. |

**Table S1 (part 1 of 2).** Primary studies that met the inclusion criteria. *Note: Table S1 is presented in two parts; see part 2 for continuation.*

| | | | | | |
|---|---|---|---|---|---|
| **Orr et al. (2017)** | Migraine and Mental Health in a Population-Based Sample of Adolescents | To explore the relationship between migraine, mood/anxiety disorders, and perceived mental health in a large, population-based sample of Canadian adolescents. | The study analyzed data from 61,375 Canadian adolescents (ages 12-19) from a national health survey. The primary group of interest was those who self-reported a physician's diagnosis of migraine. | Adolescents from the same national survey who did not report a diagnosis of migraine. | Data Source: The Canadian Community Health Survey (CCHS). Psychiatric/Headache Assessment: Self-report questions on physician-diagnosed migraine, mood disorders, and anxiety disorders. A subset of participants also completed the Composite International Diagnostic Interview—Short Form (CIDI-SF) for depression. |
| **Philipp et al. (2019)** | Prevalence and burden of headache in children and adolescents in Austria—a nationwide study in a representative sample of pupils aged 10–18 years | To estimate the prevalence and attributable burden of primary headache disorders in a representative nationwide sample of children and adolescents in Austria. | The study surveyed 3,386 pupils (ages 10-18) across Austria. The key groups analyzed were those with a headache diagnosis, including 821 with migraine, 731 with tension-type headache (TTH), 101 with frequent headache (H15+), and 884 with undifferentiated headache (UdH)—a mild, short-lasting headache type. | Pupils from the same nationwide sample who did not report headaches in the last year (n=824) served as the control group. | Headache Assessment: The Headache-Attributed Restriction, Disability, Social Handicap and Impaired Participation (HARDSHIP) questionnaire, which applies ICHD-3 criteria. Quality of Life: The KIDSCREEN questionnaire to assess health-related quality of life (HrQoL). |
| **Rabner et al. (2018)** | Pediatric Headache and Sleep Disturbance: A Comparison of Diagnostic Groups | To examine whether sleep disturbance differs across different pediatric headache diagnoses (migraine, tension-type headache [TTH], and new daily persistent headache [NDPH]) and to assess if this difference persists after accounting for other psychosocial factors. | The study included 527 children and adolescents (ages 7-17; mean age mean 13.3±52.7 years) with a primary headache diagnosis, who were divided into three comparison groups: 278 patients with migraine, 157 patients with tension-type headache (TTH), and 92 patients with new daily persistent headache (NDPH). | This study's design was a comparison among three different clinical headache groups. | Sleep Assessment: Sleep Hygiene Inventory for Pediatrics (SHIP), a parent-report measure. Psychological Assessment: Children's Depression Inventory (CDI) and Revised Children's Manifest Anxiety Scale-Second Edition (RCMAS-2). Disability Assessment: Functional Disability Inventory (FDI). Headache Diagnosis: International Classification of Headache Disorders (ICHD-2) criteria. Pain: A patient-reported rating of typical headache intensity utilizing the numerical rating scale (NRS) was obtained during the evaluation with the psychologist. NRS headache intensity was coded from “no pain” (0) to “worst pain experienced” (10). |
| **Rizvi et al. (2024)** | Anxiety and depressive symptoms and migraine-related outcomes in children and adolescents | To explore the longitudinal relationship between anxiety and depressive symptoms and future migraine outcomes (headache frequency and disability) in children and adolescents. | The study followed a single clinical cohort of 123 children and adolescents (median age 14 years) with a diagnosis of migraine. | This study was a single-cohort longitudinal study and did not include a control group. | Migraine Outcomes: Headache frequency (days/month) and the Pediatric Migraine Disability Assessment (PedMIDAS). Psychological Assessment: The Patient-Reported Outcomes Measurement Information System (PROMIS) pediatric scales for Anxiety |

**Table S1 (part 1 of 2).** Primary studies that met the inclusion criteria. *Note: Table S1 is presented in two parts; see part 2 for continuation.*

| | | | | | |
|---|---|---|---|---|---|
| | | | | | and Depressive Symptoms. Headache Diagnosis: International Classification of Headache Disorders, 3rd edition (ICHD-3) criteria. |
| **Romano et al. (2020)** | Primary headache in childhood associated with psychiatric disturbances: an update | To report the frequency of behavioral and emotional comorbidities in children with primary headaches compared to a healthy control group. | 475 children and adolescents (ages 4-14; 61% male) with a primary headache diagnosis, subdivided into 114 with migraine and 361 with tension-type headache (TTH). | 475 children (ages 4-14) without a history of headache, matched for age, sex, race, and socioeconomic status. | Psychiatric/Behavioral Assessment: Information on comorbidities (ADHD, learning disabilities, tics, anxiety, depression, OCD) was collected through direct interviews with children and their parents. Headache Diagnosis: International Classification of Headache Disorders, 2nd edition (ICHD-II) criteria. |
| **Sciruicchio et al. (2023)** | Clinical Correlates of Osmophobia in Primary Headaches: An Observational Study in Child Cohorts | To evaluate the prevalence of osmophobia (intolerance to odors) in children with primary headaches and investigate its association with headache severity, disability, and psychological factors like anxiety, depression, and pain catastrophizing. | The study included 645 children and adolescents (ages 8-15) with a primary headache diagnosis. The primary comparison was between two subgroups from this cohort: 186 patients who experienced osmophobia and 459 patients who did not experience osmophobia. | This study's design was a comparison between subgroups of patients with headaches. | Psychological Assessment: Psychiatric Self-Administration Scales for Youths and Adolescents (SAFA) for anxiety and depression, and the Child Version of the Pain Catastrophizing Scale. Headache Assessment: Clinical interviews based on ICHD-3 criteria to assess headache characteristics, allodynia, and osmophobia. Disability Assessment: Pediatric Migraine Disability Score (PedMIDAS). |
| **Sciruicchio et al. (2019)** | Pain Catastrophizing in Childhood Migraine: An Observational Study in a Tertiary Headache Center | To evaluate pain catastrophizing in children with episodic versus chronic migraine and to test its association with disability, psychological symptoms (anxiety, depression), and signs of central sensitization. | The study included 190 children and adolescents with a diagnosis of migraine, who were divided for analysis into two groups: 146 with episodic migraine (EM) and 44 with chronic migraine (CM). | The study's design was a comparison between episodic and chronic migraine groups. | Pain Catastrophizing: Child version of the Pain Catastrophizing Scale (PCS-C). Psychological Assessment: Psychiatric Self-Administration Scales for Youths and Adolescents (SAFA) for anxiety and depression. Disability/Quality of Life: Pediatric Migraine Disability Assessment (PedMIDAS) and Pediatric Quality of Life Inventory (PedsQL). Headache/Sensitization Assessment: Allodynia questionnaire and Total Tenderness Score. |

**Table S1 (part 1 of 2).** Primary studies that met the inclusion criteria. *Note: Table S1 is presented in two parts; see part 2 for continuation.*

| | | | | | |
|---|---|---|---|---|---|
| **Shimomura et al. (2021)** | Emotional and behavioral problems in pediatric patients with migraine and tension-type headache | To investigate the association between the psychopathological characteristics of children with primary headaches (measured by the SDQ) and their subsequent treatment outcomes. | The study initially included 124 children and adolescents (median age 11 years) with a primary headache diagnosis, divided into two groups: 98 with migraine. 26 with tension-type headache (TTH). A subgroup of 90 patients was followed for at least one year to assess treatment outcomes. | The study's design was a comparison between two clinical headache groups. | Psychological Assessment: The Strengths and Difficulties Questionnaire (SDQ), a parent-report measure of emotional and behavioral problems. Headache Diagnosis: International Classification of Headache Disorders, 3rd Edition (ICHD-3) criteria. Treatment Outcome: Defined as a 50% or greater reduction in headache frequency. |
| **Öztop et al. (2016)** | Assessment of Psychopathology and Quality of Life in Children and Adolescents With Migraine | To investigate comorbid psychiatric disorders, anxiety and depression levels, and quality of life in children with migraine, and to assess their relationship with the migraine condition. | 35 children and adolescents (ages 9-16; mean age 12.2±1.95 years) with a diagnosis of migraine. | 35 healthy children (mean age 12.2+1.95 years) and adolescents, matched for age and sex. | Psychiatric Diagnosis: A semi-structured interview, the Kiddie Schedule for Affective Disorders and Schizophrenia (K-SADS-PL). Psychological Symptoms: Children's Depression Inventory (CDI) and State-Trait Anxiety Inventory for Children (STAI/STAI-C). Disability/Quality of Life: Pediatric Migraine Disability Assessment Tool (PedMIDAS) and Pediatric Quality of Life Questionnaire (PedsQL). Pain: Visual Analog Scale (VAS) (0-10). |
| **Strong et al. (2021)** | New Daily Persistent Headache in a Pediatric Population | To describe the clinical characteristics of New Daily Persistent Headache (NDPH) in pediatric patients presenting to a headache program at a tertiary referral center. | 245 pediatric patients (mean age 14.7±2.8 years) diagnosed with New Daily Persistent Headache (NDPH). | This was a descriptive, retrospective study, and therefore did not include a control group. | Data were collected through a retrospective review of medical records from an IRB-approved patient registry. A standardized headache history intake form was used for patient evaluation. Pain intensity was assessed using the Wong–Baker FACES scale. Diagnoses of NDPH and medication overuse headache were based on the International Classification of Headache Disorders, 3rd Edition (ICHD-3) criteria. |
| **Syed & Mullick (2025)** | Psychiatric Co-Morbidities Among Children and Adolescents With Headache: Findings From a Cross-Sectional Study in Bangladesh | To explore the prevalence, types, and correlates of psychiatric disorders among children and adolescents with primary headaches in Bangladesh, and to examine the relationship between headache characteristics and psychiatric co-morbidities. | 151 children and adolescents (ages 5 to 17 years), with primary headaches. | The study did not include a control group. | Headache types were classified using the International Classification of Headache Disorders, 3rd edition beta version (ICHD-III beta). Psychiatric diagnoses were generated using the validated Bangla version of the Development and Well-Being Assessment (DAWBA), based on DSM-IV criteria. |

**Table S1 (part 1 of 2).** Primary studies that met the inclusion criteria. *Note: Table S1 is presented in two parts; see part 2 for continuation.*

| | | | | | |
|---|---|---|---|---|---|
| **Tarantino et al. (2015)** | Migraine equivalents and related symptoms, psychological profile and headache features: which relationship? | The primary aim was to compare the psychological profiles (specifically anxiety and somatization) of children with migraine who also experience migraine equivalents and related symptoms (MERS) against those who do not. A secondary aim was to investigate, within the MERS group, the relationship between psychological factors and headache frequency/intensity. | 101 children and adolescents (age 8-17; mean age 11.5 ± 2.3 years) diagnosed with migraine without aura who also had a history of migraine equivalents and related symptoms (MERS). | 35 children and adolescents (mean age 11.5 ± 2.3 years) with migraine without aura who did not have a history of migraine equivalents and related symptoms (MERS) | Headache Classification: International Classification of Headache Disorders, 3rd edition (ICHD-III). Clinical Data: A two-month headache diary was used to record attack frequency and intensity. Psychological Assessment: The SAFA (psychiatric scales for self-administration for youths and adolescents) battery, specifically the SAFA-A scale for anxiety and the SAFA-S scale for somatization, were administered. |
| **Tarantino et al. (2017)** | Role of the Attachment Style in Determining the Association Between Headache Features and Psychological Symptoms in Migraine Children and Adolescents. An Analytical Observational Case–Control Study | To investigate the role of attachment style on headache severity (frequency and intensity) and psychological symptoms (anxiety, depression, somatization) in children and adolescents with migraine, and to explore the association between these three factors. | A cohort of 90 children and adolescents (age 8-18; mean age 12.262.6 years) diagnosed with migraine without aura. For analysis, this group was subdivided based on headache frequency, headache intensity, and attachment style. | The study did not include an external control group (e.g., healthy children); comparisons were made between subgroups within the patient cohort. | Headache Classification: International Classification of Headache Disorders, 3rd edition (ICHD-III).Clinical Data: A two-month headache diary. Attachment Style Assessment: Separation Anxiety Test (SAT), a semi-projective interview. Psychological Assessment: The SAFA battery of self-report questionnaires for Anxiety (SAFA-A), Depression (SAFA-D), and Somatization (SAFA-S). |
| **Togha et al. (2023)** | The prevalence and impact of tension-type headache in school-aged children in Iran | To investigate the prevalence of tension-type headache (TTH) among students aged 6 to 18 in various geographical regions of Iran and to assess the impact of these headaches on their quality of life. | 950 children and adolescents (age 6-18) with Tension-Type Headache (TTH). This group was further specified as 873 individuals (mean age 12.9±2.9 years) with low-frequency episodic TTH (LFET); 58 individuals (mean age 14.2±2.3 years) with high-frequency episodic TTH (HFET); and 19 individuals (mean age 14.7±1.9 years) with chronic TTH. | 1,081 children and adolescents (mean age 10.82±3.404 years) from the same schools who did not have any form of headache | A structured, self-completed questionnaire (Persian version of the HARDSHIP questionnaire) was used to gather data. Diagnoses of TTH were confirmed by neurologists according to the International Classification of Headache Disorders, 3rd edition (ICHD-3) criteria. |
| **Torres-Ferrus et al. (2019)** | Headache, comorbidities and lifestyle in an adolescent population (The TEENs Study) | To evaluate the prevalence and characteristics of headache in an adolescent population and to assess its relationship with medical comorbidities and lifestyle factors. | 494 adolescents (mean age 14.72±1.79 years) who reported suffering from recurrent headaches. For further analysis, this group was subdivided into 184 adolescents with probable migraine and 310 adolescents with other headaches. | 1125 adolescents (mean age 14.31±1.79 years) from the same school-based sample who did not report recurrent headaches. | A custom anonymous questionnaire was used to collect data on demographics, lifestyle, and headache features. Headache-related disability was measured with the Pediatric Migraine Disability Assessment Scale (PedMIDAS), and behavioral/emotional problems were screened using the Strengths and Difficulties Questionnaire (SDQ). Probable migraine was defined using ICHD-3 beta criteria. |

**Table S1 (part 1 of 2).** Primary studies that met the inclusion criteria. *Note: Table S1 is presented in two parts; see part 2 for continuation.*

| | | | | | |
|---|---|---|---|---|---|
| **Uçar et al. (2020)** | Irritability and its relationships with psychological symptoms in adolescents with migraine: a case-control study | To investigate self- and parent-reported irritability in adolescents with migraine and to evaluate the relationship between irritability and other psychological symptoms, such as anxiety and emotional problems. | 71 adolescents (mean age 14.45±2.48 years) diagnosed with migraine without aura. Their parents also participated in the study. | 41 age- and sex-matched healthy adolescents (mean age 15.24±1.67 years) with no history of psychiatric, neurological, or chronic physical disorders. Their parents also participated. | Headache diagnosis was based on ICHD-3 beta criteria. Irritability was assessed using the Affective Reactivity Index (ARI) (self- and parent report). Anxiety was measured with the Screen for Child Anxiety-Related Emotional Disorders (SCARED) (self-report). Emotional and behavioral problems were screened with the Strengths and Difficulties Questionnaire (SDQ) (parent report). Pain severity was rated using a Visual Analog Scale (VAS). |
| **Uyar Cankay & Besenek (2021)** | Negative effects of accompanying psychiatric disturbances on functionality among adolescents with chronic migraine | To examine the effect of psychiatric symptoms on headache severity and functionality loss among adolescents with chronic migraine. The primary aim was to compare psychiatric diagnoses and symptoms in adolescents with chronic migraine against those of healthy controls. | 50 adolescents (mean age 15.5±1.45 years.) who received a first-time diagnosis of chronic migraine. | 50 healthy adolescents who were classmates of the patients and were matched for gender, age, and socioeconomic level. | Socio-demographical Questionnaire. Headache diagnosis was based on ICHD-3 criteria. Headache severity was measured with the Visual Analog Scale (VAS), and disability was assessed with the Pediatric Migraine Disability Assessment Scale (PedMIDAS). Psychiatric symptoms were screened with the DSM-5 Level 1 Cross-Cutting Symptom Measure (CCSM-5), and diagnoses were confirmed with the K-SADS-PL-DSM-5 semi-structured interview. |
| **Villa et al. (2016)** | Visual Attention in Children With Migraine: The Importance of Prophylaxis | To compare the visual attention performance of children newly diagnosed with migraine against children undergoing migraine prophylaxis and a healthy control group. | The study included two experimental groups of children aged 8 to 12 (mean age, 10.8 ±1.5 years):<br>- Untreated Migraine Group: 30 children newly diagnosed with migraine who were not on any prophylactic medication;<br>- Migraine Prophylaxis Group: 22 children with migraine who had been on prophylactic treatment for 4 to 6 months. | 30 healthy children (mean age 9.9+1.3 years) with no history of headache, recruited from public schools. | Headache diagnosis was based on ICDH-II criteria. Visual attention was assessed using a battery of tests, including the Trail Making Test (Parts A and B), the Letter-Cancellation Test, and the computerized Test of Visual Attention 3rd Edition. The WISC-III was used for general psychological evaluation and IQ screening. |
| **von Gontard et al. (2019)** | Incontinence and headache in preschool children | To examine the associations between headache, psychological symptoms, and different types of incontinence (nocturnal enuresis, daytime urinary incontinence, fecal incontinence) in a population-based sample of preschool children. | 585 preschool children (mean age 5.8±0.4 years). For analysis, the following groups of interest were identified:<br>- Headache Group (n=158): Comprising children with primary headache (n=66) and secondary headache (n=92);<br>- Incontinence Group (n=54): Comprising children with nocturnal enuresis (n=45), daytime urinary incontinence (n=14), and fecal incontinence (n=7). | The control/comparison groups were derived from the same population sample: No Headache Group (n=422). Continent Children (n=523). | A custom parent-completed questionnaire was used to collect data on headache (based on ICHD criteria) and incontinence (based on ICCS and DSM-5 criteria). Psychological symptoms were assessed using the parent version of the Strength and Difficulties Questionnaire (SDQ). |

**Table S1 (part 1 of 2).** Primary studies that met the inclusion criteria. *Note: Table S1 is presented in two parts; see part 2 for continuation.*

| | | | | | |
|---|---|---|---|---|---|
| **Wagner et al. (2015)** | Neurodevelopmental and mental health comorbidities in children and adolescents with epilepsy and migraine: a response to identified research gaps | To determine the distribution and risk of neurodevelopmental and mental health comorbidities among children and adolescents (age 6-18) with epilepsy or migraine, using a group with lower extremity fractures (LEF) as a comparison. | The study included two case groups of children and adolescents (age 6-18) identified from a statewide healthcare database: Epilepsy Group: 6,730 individuals (mean age 14y 2mo [SD 4y 5mo]). Migraine Group (Neurological Comparison): 10,495 individuals (mean age 15y 6mo [SD 2y 6mo]). | 15,305 children and adolescents (mean age 13y 11mo [SD 2y 11mo]) from the same database who were diagnosed with a lower extremity fracture (LEF). This group was intended to represent the general pediatric population. | Data were extracted from a statewide surveillance database of hospital, outpatient, and emergency department visits. Diagnoses for the case groups and comorbidities were identified using International Classification of Disease, 9th revision, Clinical Modification (ICD-9-CM) codes. |
| **Walter et al. (2021)** | Obesity, Migraine, and Overlapping Comorbidities in a Rural Pediatric Population | To report the prevalence of obesity and overlapping comorbidities (obstructive sleep apnea, depression, and anxiety) in a rural population of children and adolescents with migraine. | The study's primary group of interest consisted of 171 children and adolescents (age 7-17) from a rural clinic who had a diagnosis of migraine and met the criteria for obesity (BMI ≥ 95th percentile). | The comparison group consisted of 477 children and adolescents from the same clinic who had a diagnosis of migraine but did not meet the criteria for obesity. The study did not include a healthy, non-migraine control group. | Data were extracted from a university's electronic medical record database (Epic Clarity system). Diagnoses of migraine and comorbidities were identified using ICD-9 or ICD-10 codes. Body mass index (BMI) was calculated using height and weight data from the medical records, with obesity defined according to CDC growth charts. |
| **Yavuz et al. (2023)** | Assessment of headache in children with psychiatric symptoms | To evaluate the relationship between headaches and psychiatric symptoms in a psychiatric clinic. | 102 children and adolescents (age 8-16) who were presenting to a university child and adolescent psychiatry clinic for the first time. | 100 children and adolescents of a similar age (age 8-16) who were classmates and neighbors of the study group and reported no psychiatric complaints. | Headache burden and type were evaluated with the Headache-Attributed Restriction, Disability, Social Handicap, and Impaired Participation (HARDSHIP) Questionnaire. Psychiatric symptoms were assessed with multiple standardized scales, including the Children's Depression Inventory (CDI), State/Trait Inventory for Children (STAI), Strength and Difficulties Questionnaire (SDQ), Conners' Parent Rating Scale (CPRS-RS), and various sleep questionnaires (SQS and SVQ). |

**Table S1 (part 1 of 2).** Primary studies that met the inclusion criteria. *Note: Table S1 is presented in two parts; see part 2 for continuation.*

| | | | | | |
|---|---|---|---|---|---|
| **Miscioscia et al. (2022)** | Emotional Experience and Regulation in Juvenile Primary Headaches: A Cross-Sectional Pilot Study | To examine the interplay between emotional experience, emotion regulation, and internalizing symptoms in children with primary headaches and their caregivers, and to identify potential predictors of internalizing symptoms. | 50 children and adolescents (mean age 11.66±2.25 years) with a diagnosis of primary headache, subdivided into Migraine (n=31) and Tension-Type Headache (n=19), and their mothers (mean age 44.55±4.71). | 51 healthy children and adolescents (mean age 11.73±2.32 years), and their mothers (mean age 44.90±5.66 years). | Headache diagnosis was based on ICHD-3 criteria. Psychological functioning was assessed with multiple self- and parent-report questionnaires, including the Strength and Difficulties Questionnaire (SDQ) for psychological adjustment, the Emotion Awareness Questionnaire (EAQ), the Positive and Negative Affects Schedule (PANAS), and the Difficulties in Emotion Regulation Scale (DERS) for parents. |
| **Galli et al. (2017)** | Headache and psychological disorders in children and adolescents: a cross-generational study | To analyze the familial recurrence of headache and to compare the psychopathological profile of children with headache against healthy controls. A secondary aim was to compare children's and mothers' perceptions of psychopathological symptoms. | 130 patients (mean age 12.32±2.69) with a headache diagnosis, recruited from a headache center. This group was further subdivided into Migraine (n=104) and Other headache sub-types (n=26). | 87 healthy subjects (mean age 12.63±2.95) without a significant history of headache, recruited from local schools. | Headache diagnoses were established using a structured interview based on ICHD-II criteria. Psychopathology was assessed using both a parent-report measure, the Child Behavior Checklist (CBCL), and a child self-report measure, the Self Administrated Psychiatric Scales for Children and Adolescents (SAFA), focusing on anxiety and depression. |
| **Onofri et al. (2022)** | Correlation between primary headache and learning disabilities in children and adolescents | To evaluate the relationship between primary headaches (H) and learning disabilities (LD), considering the influence of psychopathology and the impact on quality of life via school absences. | 93 children and adolescents (mean age 10y 7m). The sample was divided into three groups for comparison:<br>- Group 1 (H only): 122 patients with a primary headache diagnosis but no LD. N=46 with migraine without auraMwoA); N=22 with migraine with aura (MwA); N=19 with chronic migraine (CM); N=16 with TTH; N=9 with chronic tension headache (CTH); N=10 with double diagnosis (DD).<br>- Group 2 (LD only): 37 patients with a learning disability diagnosis but no primary headache<br>- Group 3 (H+LD): 34 patients with diagnoses of both primary headache and a learning disability. | The study did not include a traditional healthy control group of children with neither headaches nor learning disabilities. It used a comparative design by dividing its clinical sample of 193 children into three distinct groups, which were then compared against each other. | Headache diagnosis was based on ICHD-3 criteria. Learning disabilities were assessed with standardized Italian assessment batteries (e.g., MT-Tests, AC-MT). Psychopathology was screened for with a clinical interview and the Self Administrated Psychiatric Scale for Children and Adolescents (SAFA). Impact on quality of life was measured by school absences. |

**Table S1 (part 1 of 2).** Primary studies that met the inclusion criteria. *Note: Table S1 is presented in two parts; see part 2 for continuation.*

| | | | | | |
|---|---|---|---|---|---|
| **Şentürk et al. (2023)** | The prevalence and impact of primary headaches in orphaned children: a cross-sectional, observational study in a boarding school | To investigate the prevalence, clinical characteristics, and daily life impact of migraine and tension-type headache in orphaned children with low income attending a boarding school. | 415 orphaned children (age 12-17) from a single boarding school in Turkey. For analysis, this cohort was divided into three main groups: - Migraine Group: 100 students (24.1%) with a diagnosis of migraine. - Tension-Type Headache (TTH) Group: 148 students (35.7%) with a diagnosis of TTH. No Headache Group: 167 students (40.2%) who did not report primary headaches. | No Headache Group: 167 students (40.2%) who did not report primary headaches. | A Structured Headache Questionnaire was used to gather data and diagnose headache types based on ICHD-3 beta criteria. Headache severity was measured with the Visual Analogue Scale (VAS), disability with the Pediatric Migraine Disability Assessment Scale (PedMIDAS), and psychological issues with the Strength and Difficulties Questionnaire (SDQ). |
| **Erdoğan et al. (2025)** | Restless legs syndrome and headache cause sleepiness and consequent poor school performance: a community-based study from Turkey | To estimate prevalence of restless legs syndrome (RLS) and headache (with type) in adolescents; to examine link between daytime sleepiness (ESS) and academic success. | 3.2% of 4,151 adolescents RLS-positive; 46.9% of adolescents with headache (5.2% with migraine, 17,7% with TTH); adolescents with high daytime sleepiness. | RLS-negative, no headache, lower ESS adolescents. | Demographic interview form; RLS diagnostic criteria; headache questions to classify migraine vs TTH; Epworth Sleepiness Scale (ESS) for daytime sleepiness; academic success metric. |

**Table S1 (part 2 of 2).** Primary studies that met the inclusion criteria (continued from part 1).

| Authors and Year | Cognitive/Psychological Aspects Investigated | Methods (experimental design and analysis) | Results | Limitations |
|---|---|---|---|---|
| **Caruso et al. (2022)** | The study focused on several psychological constructs: health mindsets (fixed vs. growth), cognitive appraisals of treatment setbacks (as a threat or challenge), coping strategies (active vs. passive), quality of life, and life satisfaction. | Cross-sectional, correlational study. Analysis: Pearson correlations, multiple linear regressions, and path analysis (Structural Equation Modeling) to test for direct and indirect associations. | Children with higher growth health mindsets were less likely to appraise treatment setbacks as threatening and reported fewer quality-of-life problems, higher life satisfaction, and lower functional disability. An indirect relationship was found where a growth mindset was associated with less passive coping through the mechanism of lower threat appraisal of setbacks. | The sample size (N=88) was relatively small, limiting statistical power. The cross-sectional, correlational design does not allow for determining causality. Data was collected at a single time point, and the sample lacked gender, ethnic, and racial diversity. The use of vignettes for assessing responses to setbacks may not perfectly reflect actual behavior. |
| **Açıkel et al. (2021)** | Depression, anxiety, somatization (physical symptoms without a clear medical cause), anxiety sensitivity (fear of anxiety-related sensations); sleep disturbances; quality of Life (QoL). | Comparative study where children from a neurology clinic and a healthy control group completed several psychological questionnaires. Parents also completed questionnaires about their child's quality of life and sleep habits. The researchers then compared the results between the three groups. | The migraine group had more issues (depression, somatization, sleep problems) and lower quality of life (QoL) than the other groups. The tension-type headache (TTH) group only differed from controls in having more sleep problems. For children with headaches, depression was the strongest predictor of poor psychosocial QoL. | The study's sample size was small, especially for the TTH group. Participants were recruited from a clinic, not from the general community, which may limit how much we can generalize the findings. Also, the data on sleep and psychiatric symptoms came from questionnaires, not objective measures or clinical interviews. |
| **Agessi et al. (2017)** | Temporal Processing: The brain's ability to perceive the timing and order of sounds. This was assessed with the GIN and DPT tests; auditory Selective Attention: The ability to focus on specific sounds while filtering out others, also known as the "figure-ground" skill. This was assessed with the SSI and NVDT tests. | The study compared two groups of children. Each child underwent four different auditory tests in a sound-treated room. The researchers then statistically compared the test scores between the migraine group and the control group. | Children with migraine performed significantly worse than the control group on three of the four tests: the DPT, SSI, and NVDT. This suggests that they have more difficulty with ordering sounds and with focusing on specific sounds in a noisy or distracting environment. There was no significant difference between the groups on the GIN test, which measures the ability to detect gaps in noise. | The study had a small sample size (only 14 children in each group). The distribution of sexes within the groups was not equal, which could have influenced the results. |
| **Güler Aksu et al. (2022)** | Early Maladaptive Schemas (EMS): The study focused on identifying specific negative thought patterns. These are grouped into five domains: 1) Disconnection and Rejection (e.g., feelings of abandonment, shame); 2) Impaired Autonomy and Performance (e.g., feelings of dependence, failure); 3) Impaired Limits (e.g., lack of self-control); 4) Other-Directedness (e.g., self-sacrifice, seeking approval); 5) Hypervigilance and Inhibition (e.g., negativity, high standards). | This was a cross-sectional study that analyzed data from 171 adolescents with migraine from a clinic database. The researchers compared the clinical features of the migraines and the scores on the EMS questionnaire between males and females. | Females with migraine scored significantly higher on a wide range of maladaptive schemas, particularly those related to feeling disconnected, rejected, ashamed, dependent, vulnerable, and pessimistic. Males with migraine only scored significantly higher on one schema: insufficient self-control/self-discipline. A history of sexual abuse was significantly related to higher scores on several schemas, but the presence of other psychiatric disorders (like anxiety or depression) was not. | The study was conducted at a single clinic, so the results might not apply to all adolescents with migraine. There was no control group of healthy adolescents, which makes it hard to say if these schemas are specific to migraine. The data on schemas was self-reported, which could lead to bias. The study did not use formal psychological tests to assess for things like childhood adversity. |

**Table S1 (part 2 of 2).** Primary studies that met the inclusion criteria (continued from part 1).

| | | | | |
|---|---|---|---|---|
| **Güler Aksu et al. (2023)** | Early Maladaptive Schemas (EMS): The core focus was on identifying dysfunctional thought patterns related to disconnection, impaired autonomy, etc. Depression and Anxiety: Investigated as both outcomes and potential contributing factors. | A cross-sectional study that compared questionnaire results from adolescents with episodic migraine and those with chronic migraine from a single clinic's database. | Adolescents with chronic migraine had significantly higher scores on schemas related to feeling defective/ashamed, mistrustful, abandoned, and overly enmeshed with others. They also scored higher in the "disconnection/rejection" and "other-orientation" schema domains. Adolescents with chronic migraine also reported higher levels of both depression and anxiety. A history of sexual abuse was related to higher schema scores, but having a psychiatric diagnosis was not. | The study had a small number of participants with chronic migraine. It was conducted at a single clinic, which limits the generalizability of the findings. The data relied on self-reported questionnaires, which can be biased. The study design was cross-sectional, so it could not determine cause and effect. |
| **Amouroux et al. (2017)** | Anxiety: The study tracked symptoms of anxiety over the five-year period. Depression: It also tracked symptoms of depression to see if they were linked to headache outcomes. Headache-related Disability: The researchers looked at how headaches affected school attendance and other activities. | Longitudinal study | After five years, a little over 20% of the children no longer had headaches. Many of the children who still had headaches had fewer of them each month. The most important finding was that children with a higher depression scores at the start of the study were more likely to have worse headache-related disability five years later. Interestingly, anxiety scores did not predict how the headaches would change over time. | Because the children in the study were all from a specialized headache clinic, the results might not apply to all kids with headaches. The study used questionnaires to measure symptoms, not official psychiatric diagnoses. The headache diagnoses at the five-year follow-up were based on self-reported questionnaires, not a doctor's examination. |
| **Arruda et al. (2015)** | Psychosocial Adjustment: This was the main focus, looking at a child's ability to adapt to their environment. It was broken down into five areas: Emotional symptoms (like being worried or unhappy); Conduct problems (like fighting or having tantrums); Hyperactivity/inattention; Peer problems (like being solitary or bullied); Prosocial behavior (like being helpful and kind). | Teachers first provided information on the students' school performance. Then, the teachers interviewed the parents using standardized questionnaires to gather information about the children's headaches and their psychosocial adjustment. | Children with migraines were much more likely to have problems in almost all areas measured by the SDQ, including emotional symptoms, conduct problems, hyperactivity, and issues with peers, when compared to both healthy children and those with TTH. Children with TTH were also more likely to have emotional symptoms than healthy children, but not to the same extent as those with migraines. For children with migraines, the factors most strongly linked to these psychosocial problems were headache frequency, nausea, sensitivity to light and sound, prenatal exposure to tobacco, and poor school performance. | The headache diagnoses were based on information from the mothers, not on a direct interview with the children themselves. The study only classified the most severe type of headache a child had, so it might have missed some cases of TTH in children who also had migraines. The official criteria used to diagnose headaches might not be perfect for separating migraine and TTH in young children. |

**Table S1 (part 2 of 2).** Primary studies that met the inclusion criteria (continued from part 1).

| | | | | |
|---|---|---|---|---|
| **Arruda et al. (2017)** | ADHD: The study looked at the overall prevalence of ADHD, as well as its two main components: Inattention Hyperactivity-impulsivity | Large study conducted in Brazil with over 5,600 children. Researchers interviewed parents and teachers using standardized questionnaires to gather information about the children's headaches and any symptoms of ADHD. | ADHD overall, as well as hyperactivity-impulsivity and inattention were comorbid with migraine but not with TTH; the risk of comorbidity is higher in children with chronic migraine; a number of risk factors to the association were identified, including male gender, prenatal exposure to tobacco, headache frequency, and below average school performance. | The headache diagnoses were based on reports from the mothers, not a direct medical evaluation of the children. The study only classified the most severe type of headache a child had, so it might have missed some cases of tension-type headache in children who also had migraines. The study did not differentiate between migraine with or without aura. |
| **Attygalle et al. (2020)** | ADHD: The study looked for a connection between clinically diagnosed ADHD and migraines. It also looked at symptoms of hyperactivity, impulsivity, and inattention. Screen Time: The researchers asked parents how much time their children spent on devices like phones, laptops, and TVs. | This was a comparative study where researchers compared a group of children with migraines to a group without migraines. Both groups were screened for ADHD, and parents were asked about their children's daily screen time. | Children with migraines had higher scores on the ADHD screening test. Even though their scores were higher, the number of children who were officially diagnosed with ADHD was about the same in both groups. A significantly higher proportion of children with migraine reported having daily screentime of more than 1 h, compared to those without migraine. In contrast, children with or without clinically diagnosed ADHD failed to show such an association. | Small sample size. Because the children were all from a single hospital, the results might not apply to all children. The information about screen time was reported by the parents, so it might not be completely accurate. The study did not rule out other conditions that can look like ADHD. |
| **Balottin et al. (2018)** | Personality Functioning: The core structure of personality. Emotion Regulation: How adolescents manage and express their feelings. Coping and Stress Management: How they deal with situational stress. Self-Perception: Self-esteem and self-awareness. Thought Processes: The maturity and clarity of their thinking. | A comparative study where three groups (migraine, epilepsy, healthy) were assessed using a multi-method approach combining questionnaires and the Rorschach test. The results were then statistically compared across the groups. | According to parents (CBCL), adolescents with migraine had more internalizing problems (anxiety, somatic complaints) than the other groups. The Rorschach test revealed a specific personality profile for the migraine group: they showed significant difficulty in modulating their emotions, often resulting in impulsive and intense emotional expression. Adolescents with migraine also displayed more immature thought processes and a more negative self-image compared to the other groups. | The sample sizes for all groups were small, which means the results cannot be easily generalized. The epilepsy group included many different types of epilepsy, making it a very diverse comparison group. |
| **Basile et al. (2023)** | Personality Traits: The study focused on the "Big Five" personality traits to see if they changed during the lockdown. Mental Health: The researchers also looked at how things like friendships, school problems, and bullying might have been affected. | Longitudinal study. The researchers also interviewed teenagers to learn about other parts of their lives. | After the lockdown, the teenagers in the study showed a significant increase in conscientiousness. The study also found that many teenagers reported that they had fewer problems with school and bullying during the lockdown period. | Small sample size (only 15 participants), which means the results might not apply to a larger group of people. Because there was no control group of teenagers without headaches, it is hard to know if the changes were specific to kids with headaches or if they would have happened in any teenager. |

**Table S1 (part 2 of 2).** Primary studies that met the inclusion criteria (continued from part 1).

| | | | | |
|---|---|---|---|---|
| **Albanês Oliveira Bernardo et al. (2020)** | Osmophobia: Aversion to odors during a headache attack. Odor Triggers: Investigating if specific smells could start a headache. Headache-related Disability: The functional impact of the headaches. Anxiety and Depression: Assessed as co-occurring conditions. | Cross-sectional study. A neurologist evaluated each participant using standardized questionnaires. | Osmophobia is a strong indicator of migraine. In total, 98.5% of the children who experienced osmophobia had migraine. Odor-triggered headaches were 100% specific to migraine. Every single child who reported that a smell triggered their headache was a migraineur. Both osmophobia and odor-triggered headaches were linked to more severe migraines (higher pain intensity, longer duration, more vomiting). Osmophobia was also associated with a greater negative impact on the child's life and a higher likelihood of needing emergency care. | The study was cross-sectional, so it cannot determine cause and effect. It was conducted at a single hospital, which may limit the generalizability of the findings. The data relied on patient recall rather than a headache diary, which could introduce bias. |
| **Blaauw et al. (2015)** | Anxiety and depression symptoms; attention difficulties; conduct problems. | Prospective cohort (Young-HUNT1→Young-HUNT2, 4 years); hierarchical logistic regressions adjusted for age, sex, and living with both parents; outcomes: recurrent headache overall and subtypes; frequency analysis; incident cases among participants without baseline headache. | Higher baseline anxiety/depression predicted recurrent headache at follow-up, particularly for migraine and unclassified headache; anxiety/depression also related to higher attack frequency. Among adolescents without baseline headache, anxiety/depression was associated with new-onset migraine; attention difficulties were linked to unclassified headache. | Self-reported, non-diagnostic psychological measures (SCL-5); potential misclassification in recognition-based diagnoses; lack of childhood stress/trauma measures; not all invitees were interviewed; possible questionnaire repetition effects; moderate reliability for the conduct scale. |
| **Bonuccelli et al. (2023)** | Self-reported psychological status (e.g., stress/anxiety/depression mentions), sleep quantity/quality, and lifestyle correlates relevant to mental health. | Retrospective observational study; email questionnaire completed for all 90 participants regarding the three periods; categorical summaries and Cohen's Kappa to assess concordance across time points; SPSS v28; stratified analyses by age and sex. | Lockdown saw a sharp drop in physical activity and rise in screen time; headache types stayed stable and overall frequency shifts were small; post-lockdown showed fewer "no-headache" reports and a modest uptick in prophylaxis/acute medication use. | Retrospective, single-center design; reliance on an ad hoc, non-validated questionnaire and self-report (recall bias); no external control group; concordance analyses do not quantify effect sizes or causality; limited generalizability. |

**Table S1 (part 2 of 2).** Primary studies that met the inclusion criteria (continued from part 1).

| | | | | |
|---|---|---|---|---|
| **Canfora et al. (2023)** | Emotional well-being: Including feelings of isolation, depression, and irritability. Daily life: How symptoms and their unpredictability affect day-to-day activities. School: The impact of migraine on attendance, performance, and the school environment. | This was a descriptive qualitative study. Researchers conducted semi-structured interviews with 30 youth–caregiver pairs from a pediatric migraine registry. They analyzed the interview transcripts to find common themes and patterns in their experiences. | Impact on emotional well-being: Participants frequently talked about feeling isolated, depressed, and irritable. They also stressed the importance of having friends and family who understand and provide support. Impact on daily life: Migraines made it hard to participate in sports, hobbies, and even simple daily activities like chores or driving. The unpredictability of attacks made planning things very difficult. Impact on school: The school environment, with its bright lights and noise, was a common trigger for migraines. This led to frequent absences and difficulty concentrating, which hurt their grades. | The participants were from a specialized migraine registry, so they might have more severe migraines than the average child. The group was not very diverse, being mostly White and female. The interviews took place during the COVID-19 pandemic, which could have influenced how the participants talked about their experiences, especially with school. |
| **Chiappedi et al. (2018)** | General Intelligence (Full Scale IQ): The study looked at the overall cognitive ability of the teenagers. Specific Cognitive Skills: The researchers were particularly interested in four key areas: Verbal Comprehension, Perceptual Reasoning, Working Memory, Processing Speed. | Case–control study where researchers compared a group of teenagers with headaches to a group without headaches. | Overall, the teenagers with headaches had IQ scores that were within the normal range, and there was no significant difference in their Full Scale IQ compared to the control group. However, the teenagers with headaches did score significantly lower in one specific area: Working Memory. This was especially true on a subtest called Digit Span, which involves remembering and repeating back numbers. They also scored lower on a verbal subtest called Similarities, which measures abstract verbal reasoning. | The study had a small sample size, which makes it hard to draw firm conclusions. The researchers did not look at the emotional functioning of the participants, which could have influenced the results. The study did not look at medication overuse, which can also affect cognitive skills. |
| **Clementi et al. (2023)** | Multidimensional Sleep Health: The main focus was a composite score based on the "Ru-SATED" framework, which looks at six aspects of sleep: Regularity, Satisfaction, Alertness, Timing, Efficiency, and Duration. Headache Characteristics: The study measured headache frequency, severity, and related disability. Emotional Health: The researchers looked at daily ratings of anxiety and sadness. | Observational study | Half of the participants had poor overall sleep health, with at least four of the six sleep dimensions being rated as "unhealthy". The most common issues were with alertness, sleep duration, and sleep regularity. A worse overall sleep health score was significantly associated with higher headache-related disability and higher anxiety. However, the sleep health score was not significantly linked to headache frequency or severity in this study. | The study was a preliminary exploration and not a full validation of the sleep health score. Because it was an observational study, it could not determine if poor sleep causes more disability or if more disability causes poor sleep. The data was collected during the COVID-19 pandemic, which could have influenced the results. The sample was limited to adolescent females, so the findings may not apply to other groups. |

**Table S1 (part 2 of 2).** Primary studies that met the inclusion criteria (continued from part 1).

| | | | | |
|---|---|---|---|---|
| **Colon et al. (2019)** | Resting-State Functional Connectivity (rs-FC): The investigation focused on alterations in the brain's intrinsic activity during a state of rest. Resting-State Networks (RSNs): Major brain networks were examined, including the Default Mode Network (DMN), Sensorimotor Network (SMN), and Executive Control Network (CEN), to identify differences between groups. | A cross-sectional neuroimaging study that compared four groups. | Compared to controls, adolescents with migraine showed more widespread alterations in functional connectivity than young adults. Adolescents exhibited greater connectivity changes in the Default Mode Network (DMN) and stronger connectivity in the cerebellum network compared to young adults. Young adults, in turn, showed stronger functional connectivity in the executive and sensorimotor networks. Disease duration and frequency were associated differently with brain connectivity in the two age groups. | The study design was cross-sectional, not longitudinal. The age-based division of groups was described as "somewhat arbitrary". Sex differences were not analyzed due to insufficient numbers in the subgroups. The study did not differentiate between migraine with and without aura. The effects of acute medications taken over the years were not controlled for. |
| **Costa-Silva et al. (2016)** | Verbal memory (short- and long-term); Attention (selective and divided); Executive functions; Speed of information processing; Language and verbal fluency. | Case–control study that compared two groups. | The group of adolescents with migraine exhibited significantly poorer performance compared to the control group on measures of short- and long-term verbal memory, attention, executive function, and information processing speed. Specifically, they scored lower on the RAVLT, took longer to complete the TMT and parts of the Stroop Test, and showed deficits in semantic verbal fluency. | The study design was cross-sectional and featured a small sample size. Participants were recruited from a specialized clinical referral center, which may not be representative of the general population of adolescents with migraine. The inclusion criteria were restrictive (e.g., excluding psychiatric comorbidities), which posed challenges for recruitment. |
| **Domany et al. (2019)** | Sleep Disorders: The prevalence of conditions such as obstructive sleep apnea (OSA), insomnia, and periodic limb movement disorder (PLMD). Sleep Architecture: The objective structure of sleep, particularly the percentage of time spent in different sleep stages (e.g., NREM2, NREM3/slow-wave sleep). | Retrospective chart review. Experimental group was statistically compared to that of a healthy control group, adjusting for potential confounding variables like age and obesity. | Among children with migraine referred for a sleep evaluation, common sleep disorder diagnoses were obstructive sleep apnea (40%), insomnia (27%), and periodic limb movement disorder (15%). A significant difference in sleep architecture was found between the migraine group and the control group. After statistical adjustments, children with migraine had a significantly higher percentage of NREM2 sleep and a significantly lower percentage of NREM3 (slow-wave sleep). | The study was retrospective and involved a sample of patients already suspected of having a sleep disorder, which introduces selection bias and limits generalizability to all children with migraine. The control group was not matched for age or BMI with the migraine group, although statistical adjustments were made. Complete baseline headache data was not available for 35% of the patient cohort. |

**Table S1 (part 2 of 2).** Primary studies that met the inclusion criteria (continued from part 1).

| | | | | |
|---|---|---|---|---|
| **Donnelly et al. (2017)** | The study specifically assessed anxious and depressive symptoms using the Anxious/Depressed subscale of the Child Behavior Checklist (CBCL). | Cross-sectional, questionnaire-based survey of a twin and family cohort. Analysis: Random-effects logistic regression modeling was used to estimate univariate and multivariate associations between conditions, which account for the correlation between related family members. | Significant associations were found between most of the pain conditions themselves and also between the pain conditions and the non-pain conditions (restless legs syndrome, iron deficiency, anxiety/depression). In multivariate analyses, many associations remained significant. For example, restless legs syndrome was strongly associated with growing pains (OR 8.50), and iron deficiency was significantly associated with migraine (OR 2.38). | The cross-sectional design does not allow for causal conclusions. Diagnoses were based on questionnaires, not clinical assessments. The response rate was relatively low, which could have introduced selection bias. The study could not always determine the dates of onset for the conditions, thus preventing temporal analysis. |
| **Finning et al. (2022)** | Mental Health: Assessed in two ways: dimensionally (the severity of symptoms via SDQ scores) and categorically (the presence of a formal psychiatric disorder via DAWBA). School Absenteeism: Measured as both the total number of days missed and as "persistent absence" (missing 10% or more of school days). | The study used data from a baseline survey in 2004 and a follow-up survey in 2007. Regression models were used to examine the associations between having an LTC at baseline and mental health and school absenteeism outcomes at both baseline and the 3-year follow-up. | Children with any LTC had worse mental health (higher SDQ scores and higher odds of a psychiatric disorder) and more school absenteeism compared to children without an LTC, both at baseline and at the 3-year follow-up. Among the specific conditions, migraines/severe headaches, neurodevelopmental disorders, and atopic conditions were the most strongly and consistently associated with both poorer mental health and higher school absenteeism. | The identification of long-term physical conditions was based on parent-report (single yes/no questions) and was not confirmed by medical records. The severity of the physical conditions was not accounted for. School absence data were also parent-reported and may have been subject to recall bias. |
| **Gatta et al. (2015)** | Alexithymia: The central focus of the study, defined as a personality trait involving: Difficulty identifying feelings. Difficulty describing feelings. An externally oriented (concrete) thinking style. | Case–control study that compared three groups (TTH, Migraine, and Healthy Controls). | Children and adolescents with tension-type headache (TTH) had significantly higher rates of alexithymia compared to both the migraine group and the healthy controls. Specifically, they had the most difficulty identifying their feelings. There was no significant difference in alexithymia levels between the migraine group and the healthy control group. There were no significant differences in alexithymia levels among the mothers of the three groups. A significant positive correlation was found between the alexithymia scores of children and their mothers in the migraine group and the control group, but not in the TTH group. | The study relied exclusively on self-report questionnaires, which may not capture the full complexity of emotional difficulties. The sample sizes were limited, and there was a higher proportion of females, which reflects the general prevalence of headache, but may limit the ability to analyze gender differences in depth. Data on paternal alexithymia was not collected. |

**Table S1 (part 2 of 2).** Primary studies that met the inclusion criteria (continued from part 1).

| | | | | |
|---|---|---|---|---|
| **Genizi et al. (2016)** | Learning Disabilities: The prevalence of formally diagnosed learning disabilities was assessed and compared between the headache groups. | Retrospective, multi-center, clinic-based chart review. Analysis: Chi-square tests, Fisher's exact test, t-tests, and multiple logistic regression to compare groups and calculate odds ratios. | Nearly a quarter (22.5%) of children with migraine also met the criteria for episodic tension-type headache, creating a "mixed headache" profile. The presence of mixed headaches was significantly less common in children who had migraine with aura compared to those with migraine without aura. This suggests a potential distinction between the underlying mechanisms of these two migraine types. Children with mixed headaches were 2.7 times more likely to have a learning disability than children with migraine alone. | The study's design was retrospective and clinic-based, which may not be representative of the general pediatric population. Data were drawn from three different clinics, which could have introduced variability in the clinical approach, despite using the same diagnostic criteria. |
| **Genizi et al. (2020)** | Sensory Processing: How adolescents process and respond to sensory stimuli (e.g., sound, touch, visual input) in their everyday environment. Pain Catastrophizing: The tendency to ruminate on, magnify, and feel helpless about painful experiences. Headache-related Disability: The functional impact of migraine on the adolescent's life. | Case–control study. The scores were then statistically compared between the groups, and regression analyses were used to identify predictors of migraine disability. | Adolescents with migraine showed a significantly lower tendency for Sensation Seeking compared to healthy controls. The migraine group also had significantly higher levels of pain catastrophizing, specifically in the areas of Rumination and Helplessness. Within the migraine group, higher levels of Rumination and Helplessness were correlated with greater migraine pain severity. Sensation Avoiding was the only significant predictor of headache-related disability (PedMIDAS score), accounting for 26% of the variance. | The study utilized a relatively small sample size, which may limit the generalizability of the findings and the ability to detect more subtle differences between groups. |

**Table S1 (part 2 of 2).** Primary studies that met the inclusion criteria (continued from part 1).

| | | | | |
|---|---|---|---|---|
| **Genizi et al. (2021)** | The study's focus was on clinical diagnosis rather than specific psychological functions. However, the presence of stress, anxiety, and depression as reported during clinical interviews was noted. | Retrospective chart review | Versus TTH, migraine showed more nausea/vomiting, photo/phonophobia, moderate–severe pain (97% vs 13%), greater activity impairment (60% vs 9%), and more frequent analgesic use. Emotional problems were more common in TTH (36% vs. 21%). ADHD comorbidity was frequent across groups (≈15–27%), highest in adolescents with migraine (27%). Within each diagnosis, headache features were broadly similar in children vs. adolescents. Unclassified headaches were more unilateral/pressing, shorter, interfered with activities more than TTH, and never self-resolved. | The study was retrospective and conducted at a single clinic, which may limit the generalizability of the findings. Data were collected through unstructured clinical interviews rather than through standardized questionnaires, which could introduce variability. The exclusion of children under 6 years old means the findings do not apply to very young patients. |
| **Genizi et al. (2025)** | Executive Functions (EF): Including skills like inhibition, emotional control, initiation, working memory, and planning. Anxiety: Both state (temporary) and trait (personality-based) anxiety. Social Participation: The extent to which children engage in age-appropriate activities. Quality of Life (QoL): Overall well-being, including physical, emotional, social, and school functioning. | Prospective case–control study | Compared to healthy controls, children with migraine showed significantly poorer executive functions (particularly in behavioral regulation), higher trait anxiety, and lower quality of life in physical, emotional, and school domains. Within the migraine group, poorer executive functions were correlated with higher anxiety, reduced social participation, and lower quality of life. An unexpected finding related to COVID-19: Healthy children who had contracted COVID-19 showed significantly worse executive functions (inhibition, initiation, metacognition) than the children with migraine who had contracted COVID-19. | The study used a small convenience sample from a single center. The groups were not perfectly matched on sociodemographic measures. The study did not examine other factors that could affect the outcomes, such as depression or stress levels. The data was collected during the unique circumstances of the COVID-19 pandemic. |

**Table S1 (part 2 of 2).** Primary studies that met the inclusion criteria (continued from part 1).

| | | | | |
|---|---|---|---|---|
| **Gerstl et al. (2021)** | The study focused on the long-term incidence of new clinical diagnoses, including affective or mood disorders; neurotic, stress-related, and somatoform disorders; behavioral syndromes; back pain; and irritable bowel syndrome. | Retrospective cohort study | Adolescents who had migraine at age 15 had a significantly higher risk of developing additional disorders over the next 10 years compared to the control group. Specifically, they had a 2.1-fold higher risk of developing an affective or mood disorder, a 1.8-fold higher risk for neurotic, stress-related, and somatoform disorders, a 1.8-fold higher risk for behavioral syndromes, a 1.6-fold higher risk for back pain, and a 1.5-fold higher risk for irritable bowel syndrome. | The study was a retrospective analysis of insurance data, and diagnoses were based on physician coding (ICD-10) rather than standardized clinical assessments, which could be prone to error. The severity of the migraine could not be assessed from the available data. There is a possibility that individuals with migraine visit physicians more often, increasing their chances of receiving additional diagnoses (detection bias). It is possible that some individuals had undiagnosed psychiatric conditions before the study's start year of 2006. |
| **Gibler et al. (2024)** | Anxiety Symptoms: Assessed via the GAD-7 screener. Depressive Symptoms: Assessed via the PHQ-9 screener. Suicidal Ideation: Assessed with a specific item on the PHQ-9. Headache-related Disability: The functional impact of migraine on school, work, and home activities. | Cross-sectional, retrospective observational study. Descriptive statistics and correlational analyses were used to characterize the sample and examine associations between variables. | The sample reported a high burden of disease, with an average of more than 19 headache days per month and nearly half reporting severe disability. A significant portion of patients reported a previous diagnosis of anxiety (26.1%) or depression (23.3%). More than one-third of the sample scored above the clinical cutoff on the GAD-7 and PHQ-9 screening tools. Higher scores on both the anxiety (GAD-7) and depression (PHQ-9) screeners were associated with greater headache frequency. Over 10% of patients endorsed current suicidal ideation, which was not found to be related to the level of headache disability. | The study was cross-sectional, preventing conclusions about causality. The sample was recruited from a single, specialized tertiary care clinic and was predominantly White and female, limiting the generalizability of the findings. Data were collected to inform clinical care, so some information (e.g., gender identity, reasons for emergency department visits) was not systematically captured. Formal psychiatric diagnoses were not assigned via structured clinical interviews; the study relied on screening tools and patient self-reports of prior diagnoses. |
| **Giricz et al. (2021)** | Associative Learning: Specifically, "acquired equivalence," which is the ability to learn that two different stimuli are functionally equivalent because they lead to the same outcome. Learning and Memory Processes: The test assesses different phases, including initial learning (acquisition), memory recall (retrieval), and applying a learned rule to new situations (generalization). | Case–control study. The scores were then statistically compared between the groups, and regression analyses were used to identify predictors of migraine disability. | The primary finding was that, unlike the adult migraine patients who showed significant deficits, there was no significant difference in performance on the learning task between the pediatric migraine patients and their healthy peers. The children and adolescents with migraine performed just as well as the controls in all phases of the task, including initial learning, retrieval, and generalization. This contrasts sharply with the adult data, where patients with migraine performed significantly worse, particularly in the generalization phase. | The study had a relatively low number of participants in each group. The adult dataset used for comparison was from a previous study, not collected concurrently. |

**Table S1 (part 2 of 2).** Primary studies that met the inclusion criteria (continued from part 1).

| | | | | |
|---|---|---|---|---|
| **Goenka & Fonseca (2023)** | Treatment Response: The primary focus was on identifying factors that predict a poor response to intensive, inpatient migraine treatment. Psychiatric Comorbidities: The study specifically examined the prevalence of Attention-Deficit/Hyperactivity Disorder (ADHD) and the severity of Generalized Anxiety Disorder (GAD) as potential predictors of treatment outcome. | Retrospective cohort study. Patients were categorized as "responders" or "non-responders" based on the reduction in their pain scores from admission to discharge. | Approximately 21.4% (34 out of 159) of patients were non-responders to the intensive, three-tiered treatment protocol. Non-responders had a significantly higher prevalence of comorbid ADHD compared to responders (41.2% vs. 13.6%). Among patients who also had an anxiety disorder, non-responders were significantly more likely to have severe anxiety (GAD-7 score ≥15) than responders (42.9% vs. 5.1%). Non-responders had longer hospital stays and significantly higher rates of readmission within 7 days. | The study's design was retrospective, which relies on the quality and completeness of data in the medical records. The sample was from a single, hospital-based setting, which limits the generalizability of the findings. The GAD-7 was analyzed only for a subset of patients who already had a known comorbidity of anxiety. |
| **Güler et al. (2017)** | Psychiatric Comorbidity: The prevalence of formal psychiatric diagnoses, particularly anxiety disorders, depression, and ADHD. Anxiety and Depression Symptoms: The severity of anxiety and depressive symptoms as measured by standardized scales. | Cross-sectional, clinical-based study. The study then compared the prevalence and characteristics of psychiatric comorbidities between the migraine and TTH groups. | A very high rate of psychiatric comorbidity (82.2%) was found across the entire sample of adolescents with headaches. The most common type of comorbid psychiatric disorder was an anxiety disorder. There was no significant difference in the overall rates of psychiatric disorders between the migraine and TTH groups. In patients with migraine, the co-occurrence of an anxiety disorder or ADHD was associated with a higher frequency of throbbing-type pain. In patients with TTH, co-occurring anxiety was associated with a higher likelihood of pain worsening with physical activity. | The study was cross-sectional, which does not allow for conclusions about causality. The sample was recruited from a hospital clinic, which may have resulted in a higher prevalence of comorbidities compared to the general population (selection bias). The socioeconomic and sociocultural level of the patient sample was noted to be low, which could be a contributing factor. |
| **Hartberg et al (2015)** | Mental Health Problems: The prevalence of significant emotional and behavioral difficulties as measured by the SDQ. Coping Strategies: The primary focus was on the types of coping strategies used, categorized as follows: Internal: Keeping feelings inside, substance use, trying to talk oneself out of problems. External: Seeking help from health services, talking to family or friends. | Cross-sectional survey. Logistic regression was used to compare the use of different coping strategies among the four defined groups (CH, CHMH, MH, and control). | Adolescents with chronic headaches had a much higher prevalence of mental health problems (23%) compared to those without chronic headaches (6%). The group with both chronic headache and mental health problems (CHMH) was the most vulnerable. Compared to controls, this group was significantly more likely to use internal coping strategies, such as keeping feelings inside and using substances. Adolescents in all three clinical groups (CH, CHMH, and MH) were significantly less likely to use external coping strategies, such as talking to family or friends, compared to the healthy control group. | The study's design was cross-sectional, so it cannot determine causality. The definition of "chronic headache" was based on a single question about frequency and was not a formal clinical diagnosis based on established criteria. The questions used to assess coping strategies were not from a previously validated, standardized scale. The data was based entirely on self-reports without clinical validation. |

**Table S1 (part 2 of 2).** Primary studies that met the inclusion criteria (continued from part 1).

| | | | | |
|---|---|---|---|---|
| **Oliveira-Souza et al. (2022)** | The co-occurrence (comorbidity) between a physical condition (migraine) and psychological and behavioral aspects related to eating disorders, specifically the symptoms of bulimia nervosa. | Cross-sectional, analytical study. Associations between the variables were examined using statistical analyses, including chi-square tests and logistic regression. | Adolescents with migraine showed a significantly higher prevalence of eating disorder symptoms (evaluated by the EAT-26) and bulimia nervosa symptoms (evaluated by the BITE) compared to the non-migraine group. After statistical adjustment, adolescents with migraine were 1.51 times more likely to present with symptoms of bulimia nervosa. Female adolescents had 1.85 times higher odds than males of presenting with bulimia nervosa symptoms. | The study's cross-sectional design does not allow for establishing a cause-and-effect relationship between migraine and eating disorders. The study did not evaluate for the presence of depression, which could be a confounding factor in the observed association. Diagnoses were based on screening questionnaires and not on direct clinical evaluation by a specialist, which is a methodological limitation. |
| **Cerutti et al. (2016)** | Alexithymia: A personality trait characterized by difficulty in recognizing, describing, and processing one's own emotions. Psychopathological Symptoms: The presence of psychological distress in areas such as somatization, anxiety, and depression. | Case–control study. The mean scores were then compared between groups, and linear regression was used to test the predictive role of alexithymia on psychopathology. | Both adolescents and mothers with migraine demonstrated significantly higher levels of alexithymia compared to their respective control groups. Adolescents with migraine reported more symptoms of somatization, depression, and anxiety. Mothers with migraine reported more symptoms in nearly all psychopathological areas. Alexithymia was found to be a significant predictor of the presence of psychopathological symptoms in both adolescents and mothers with migraine. | The clinical sample was from a third-level center, which may not be representative of the general population. The study relied solely on self-report questionnaires. The sample size was relatively small. The cross-sectional design does not allow for definitive conclusions about causal links. |
| **Esposito et al. (2021)** | The study investigated a wide range of neuropsychological skills, including attention, executive functions, language, memory, learning, sensorimotor functions, social perception, and visuospatial processing. | Pilot study comparing 15 children with MwoA to 38 healthy controls. The scores from the two groups on the NEPSY-II subtests were statistically compared using unpaired t-tests. | Children with MwoA showed significantly lower performance than healthy controls on several NEPSY-II subtests. These deficits were noted in tasks related to executive functions (inhibition, visuomotor precision), language (comprehension of instructions), memory (immediate and delayed memory for names), sensorimotor skills (manual motor sequences), and visuospatial processing (design copying, geometric puzzles). Conversely, the MwoA group performed significantly better than controls on tasks involving visual attention and delayed memory for faces. The frequency and intensity of migraine attacks were significantly correlated with poorer performance on tasks of visuomotor accuracy and delayed memory of faces. | The primary limitation acknowledged is the small sample size, characteristic of a pilot study, which may affect the generalizability of the results. |

**Table S1 (part 2 of 2).** Primary studies that met the inclusion criteria (continued from part 1).

| | | | | |
|---|---|---|---|---|
| **Fielding et al. (2016)** | The co-occurrence of anxiety disorders and headache symptoms (migraine and TTH) in children. Differences in headache incidence based on specific anxiety diagnoses (e.g., separation anxiety disorder). The association between anxiety symptom severity and the presence of headaches. | Between-groups, cross-sectional design. Analyses: T-tests, Pearson's $\chi^2$ test, Fisher's exact test, and one-way between-subjects ANOVAs | Children with anxiety disorders had a significantly higher incidence of headache symptoms consistent with primary headache disorders (56%) compared to control children (25%). The association was particularly strong for migraine-like symptoms. Girls with anxiety disorders had a significantly higher incidence of headaches than girls in the control group. Children with a diagnosis of separation anxiety disorder were more likely to have headaches than anxious children with other diagnoses. Children with both anxiety and headaches reported the highest levels of anxiety symptom severity. | The sample size was small. The study relied solely on parent reports for headache symptoms, which may not fully capture the child's subjective experience of pain. Headaches were not clinically diagnosed by a physician, nor were headache diaries used. The study did not include a child self-report measure for headaches. The underlying mechanisms common to both anxiety and headaches were not investigated. Parent factors, such as their own history of anxiety or headaches, were not assessed. |
| **Hommer et al. (2022)** | The comorbidity between headache subtypes and a wide range of mental disorders (mood, anxiety, eating, substance use, ADHD, and behavior disorders). Sex-specific differences in the association between headache and mental illness. The relationship between headache severity (e.g., migraine with aura) and the number of co-occurring mental disorders. | Cross-sectional analysis of data from a nationally representative population-based survey. Analyses: Cross-tabulations to estimate lifetime prevalence and multivariate logistic regression to examine associations, controlling for demographic variables and comorbid disorders. | 26.9% of adolescents reported a lifetime history of headache, with a higher prevalence in females. Youth with headache were over twice as likely (OR 2.74) to have a DSM-IV disorder compared to those without headache. Migraine, particularly with aura, showed the strongest associations with mood disorders and anxiety disorders. Significant associations were also found for headache with behavior and substance use disorders. No association was found between headache and ADHD. Adolescents with migraine, especially with aura, were more likely to have multiple mental disorders. | The cross-sectional design prevents conclusions about causality. Headache assessment was based on WHO-CIDI questions that were not designed to perfectly match ICHD-3 diagnostic criteria, limiting the characterization of headache subtypes. The initial screening question only captured "frequent or very bad headaches," likely underestimating the total prevalence by missing milder cases. The classification of migraine with aura relied only on visual symptoms, potentially missing some cases. |
| **Hsu et al. (2022)** | The risk of receiving a new migraine diagnosis over time in individuals with a pre-existing ADHD diagnosis. The potential influence of ADHD medications (methylphenidate and atomoxetine) and their cumulative dosage on the risk of developing migraine. Differences in migraine risk among ADHD patients based on age group and sex. | A nationwide, longitudinal case-cohort study using retrospective data from 2001 to 2011. Analyses: Cox regression analysis was used to calculate the hazard ratio (HR) for developing migraine, adjusting for demographics and comorbidities. Pearson's $\chi^2$ test and F-test were used for between-group comparisons. | Individuals with ADHD had a significantly higher incidence of migraine compared to the control group (0.6% vs. 0.3%). After adjusting for confounders, the risk of developing migraine was nearly double in the ADHD group (HR = 1.92). This increased risk was statistically significant for children (HR = 2.01) and adolescents (HR = 1.94) with ADHD, but not for young adults with ADHD. The cumulative dosage of ADHD medication was not found to be associated with the risk of migraine. | The use of a claims database may underestimate the true prevalence of both ADHD and migraine, as it only includes cases that sought medical care. The restrictive diagnostic criteria in the database might have resulted in a more severe ADHD sample, potentially limiting generalizability. Diagnoses based on ICD codes may not perfectly align with official diagnostic criteria (like ICHD for migraine) and could miss cases treated outside of the formal system. Data on medication usage reflects filled prescriptions, not patient adherence. Important potential confounders like sleep patterns, lifestyle, or stress were not available in the database. |

**Table S1 (part 2 of 2).** Primary studies that met the inclusion criteria (continued from part 1).

| | | | | |
|---|---|---|---|---|
| **Jafari et al. (2023)** | The prevalence and severity of anxiety and depression symptoms in adolescents with migraine compared to those without. The association between the level of anxiety and depression and the characteristics of migraine attacks (frequency, severity, duration). | Case–control study. Analyses: The chi-square test was used to compare proportions (e.g., levels of anxiety) between groups, and the independent two-sample t-test was used to compare means (e.g., age, headache frequency). | Adolescents with migraine had significantly higher rates of mild, moderate, and severe anxiety compared to the control group. A significantly greater percentage of adolescents in the control group had non-depressive scores compared to the migraine group. Within the migraine group, individuals with severe anxiety experienced significantly more frequent and longer-lasting attacks than those with moderate anxiety. Migraineurs with depression reported higher attack severity and a marginally higher frequency of attacks than those without depression. | The study was conducted during the COVID-19 pandemic, which may have increased the baseline levels of anxiety and depression in all participants. Some data were collected through online questionnaires, which might be less accurate than in-person interviews. |
| **Kemper et al. (2016)** | The correlation between headache-related disability and various psychological states, including depression, anxiety, stress, mindfulness, resilience, and self-compassion. The relationships among these different psychological risks and protective factors. Adolescents' interest in learning mind-body techniques to manage their headaches and stress. | Cross-sectional survey. Analyses: Descriptive statistics were calculated for all measures. Pearson's or Spearman's correlation coefficients were used to assess the relationships between headache disability, psychological factors, and physiological biomarkers. | The only psychological factor that was significantly correlated with higher headache-related disability was depression (r = 0.52). Depression was positively correlated with stress, anxiety, and sleep disturbance, and negatively correlated with mindfulness, self-compassion, and resilience. The physiological biomarkers of inflammation and vagal tone (HRV) were not significantly associated with headache disability or depression. The vast majority of teens (86%) expressed interest in learning stress management skills, particularly slow, deep breathing exercises supported by a smartphone app. | The study had a very small sample size (n=29). Participants were recruited from a single tertiary care institution, which limits the generalizability of the findings. The cross-sectional design means that no causal relationships can be determined. The study only included adolescents aged 12-18 and its findings cannot be extrapolated to younger children. Fonti |
| **Lateef et al. (2019)** | The association between headache subtypes and specific sleep problems (e.g., shorter sleep duration, insomnia symptoms). The mediating effect of co-occurring anxiety and mood disorders on the relationship between headaches and sleep. | Cross-sectional analysis of a large, population-based survey. Analyses: Multivariate regression models were used to assess the associations between headache status and sleep variables, adjusting for demographics and psychiatric comorbidities. | Adolescents with migraine had shorter sleep duration, earlier wake-up times, and significantly more insomnia symptoms than their peers without headaches. Youth with migraine with aura reported the most severe sleep problems, including difficulty maintaining sleep and daytime fatigue. However, these associations between headache and sleep problems were significantly weakened (and often became non-significant) after controlling for co-occurring anxiety and mood disorders, suggesting that these psychiatric conditions are a key link between the two. | The cross-sectional design cannot determine causality (i.e., whether headaches cause sleep problems or vice versa). Headache and insomnia classifications were based on survey questions that approximated only formal diagnostic criteria. The headache sample was limited to adolescents who endorsed "severe and frequent" headaches, potentially missing milder cases. |

**Table S1 (part 2 of 2).** Primary studies that met the inclusion criteria (continued from part 1).

| | | | | |
|---|---|---|---|---|
| **Law et al. (2019)** | The study assessed whether the PAT could accurately measure and predict a range of psychosocial issues, including child behavioral problems, parent emotional distress (anxiety and depression), and overall family functioning in the context of pediatric headache. | Longitudinal validation study. Analyses: Internal consistency (alpha) was calculated to assess reliability. Spearman correlations were used to establish convergent validity (correlations at baseline) and predictive validity (correlation of baseline PAT scores with 6-month outcomes). | The PAT demonstrated strong internal consistency and was found to be a reliable measure. The tool showed good convergent validity, as PAT scores were significantly correlated with measures of child, parent, and family distress at baseline. The tool also showed good predictive validity, as higher PAT scores at baseline successfully predicted worse psychosocial outcomes for the child, parent, and family at the 6-month follow-up. | The sample was predominantly Caucasian and middle class, limiting generalizability to other demographic groups. The study focused on youth with frequent headaches, so the results may not apply to those with less frequent headache patterns. The study did not assess whether PAT scores predicted long-term physical health outcomes like headache frequency or disability. |
| **Lee et al. (2015)** | The prevalence of clinically significant depression and anxiety symptoms among children presenting with headaches. The formal psychiatric diagnoses received by children who screened positive for high psychological distress. | Retrospective chart review of patients seen in a pediatric neurology clinic over a two-year period. Analyses: Descriptive statistics were used to summarize patient data. Spearman rank correlation was used to assess the relationship between screening scores and headache characteristics. | Out of 720 children with headaches, 19 (2.6%) had clinically significant scores on the depression and/or anxiety screening tools. Following psychiatric evaluation, 2.8% of the total sample received a formal psychiatric diagnosis, most commonly major depression (1.7%) and generalized anxiety disorder (1.1%). There was no significant correlation between the severity of depression or anxiety scores and the frequency, duration, or intensity of the headaches. | The study design was retrospective. The screening did not include a comprehensive assessment of all behavioral problems. The study did not include a follow-up to evaluate the effects of psychiatric treatment on headache outcomes. |
| **Lee et al. (2022)** | The study focused on how children and adolescents with migraine perceive their parents' attitudes (e.g., supportive, achievement-focused, punitive). It analyzed how these perceived attitudes correlated with the severity and frequency of their headaches. | A retrospective chart review of patient medical records. Analyses: Pearson correlation analysis was used to examine the relationships between the PAT-Y scores and headache severity metrics for each age group. | The relationship between perceived parenting attitudes and headache severity differed significantly by age. In the younger group (elementary school), parenting attitudes focused on high achievement and expectations were associated with lower headache severity. In the older group (middle school), the opposite was true: a perceived parental focus on achievement ("achievement press") was associated with higher headache frequency and severity. A perceived "neglectful" attitude was also linked to greater headache severity in this group. | The study design was retrospective. There was no control group of healthy children, which makes it difficult to determine if the observed parenting attitudes are unique to families of children with migraine. The correlational findings do not establish causality. |

**Table S1 (part 2 of 2).** Primary studies that met the inclusion criteria (continued from part 1).

| | | | | |
|---|---|---|---|---|
| **Magnerou et al. (2024)** | The study focused on the impact and burden of primary headaches on children's lives, measuring school absenteeism, interruption of daily activities, overall quality of life, and emotional state (e.g., fear of headache). | A cross-sectional, school-based study conducted in 52 primary schools in Douala, Cameroon. Analyses: Descriptive statistics were used for prevalence rates. Chi-square tests and Student's t-tests were used to compare groups, and logistic regression was used to analyze factors associated with poor quality of life. | The prevalence of any headache in the past year was extremely high at 85.7%. The prevalence of migraine was 26.1%, while tension-type headache (TTH) was 15.1%. Migraine had a significantly greater impact than TTH, causing more school absenteeism (32.8% of migraineurs) and interruption of activities (57.6% of migraineurs). Students with migraine were 2.84 times more likely to report a poor quality of life compared to students without headaches. | The study's reliance on self-report from children could introduce memory or recall bias. Findings from an urban area may not be generalizable to rural populations. Differentiating primary from secondary headaches can be difficult in this region due to the high prevalence of other conditions like malaria. |
| **Maltese et al. (2017)** | The study examined the correlation between overall psychological problems (particularly internalizing symptoms like anxiety and depression) and specific types of sleep disturbances in children with headaches. | A cross-sectional, observational study. Analyses: Correlations were calculated to assess the statistical relationship between the scores on the psychological (CBCL) and sleep (SDSC) scales. | A significant positive correlation was found between higher levels of psychological problems in children and the presence of specific sleep disorders. Greater psychological distress was associated with difficulty initiating and maintaining sleep (DIMS), disorders of arousal (DA) (e.g., night terrors, sleepwalking), and sleep–wake transition disorders (SWTD). | The cross-sectional design does not allow for determining causality (i.e., whether psychological issues cause sleep problems, or vice versa). The sample size was small (n=64). The study lacked a healthy control group for comparison. |
| **Margari et al. (2018)** | The study focused on non-verbal cognitive functions, including overall non-verbal IQ, non-verbal memory, and various attention skills (e.g., sustained attention, processing speed). | Observational, case–control study. Analyses: The Mann–Whitney U test was used to compare cognitive scores between the two groups. Spearman's correlation and multivariate regression were used to analyze the link between headache features and cognitive performance. | There were no significant overall differences in non-verbal cognitive abilities (including non-verbal IQ) between the headache group and the healthy control group. However, within the headache group, higher headache frequency and greater headache-related disability (PedMIDAS score) were significantly correlated with poorer performance in specific domains of non-verbal memory and sustained attention. | The sample size was small (35 patients, 23 controls), which limits the generalizability of the results. The study did not include a longitudinal follow-up to track cognitive performance over time. |

**Table S1 (part 2 of 2).** Primary studies that met the inclusion criteria (continued from part 1).

| | | | | |
|---|---|---|---|---|
| **Öksüz et al. (2024)** | The study compared how migraine characteristics differ depending on the type of comorbid psychiatric disorder, specifically contrasting patients with internalizing disorders (e.g., depression, anxiety) against those with externalizing disorders (e.g., ADHD). | A retrospective, observational, comparative study of patient data collected. Analyses: The Mann–Whitney U test and chi-square test were used for group comparisons, and multiple logistic regression was used to identify associated factors. | Chronic migraine (CM) was significantly more common in the group with internalizing disorders, while episodic migraine (EM) was more frequent in the group with externalizing disorders (ADHD). Patients with internalizing disorders had significantly higher pain intensity and greater headache frequency, suggesting a more severe headache burden. The group with internalizing disorders was significantly older and had a higher proportion of females. Photophobia was more prevalent in the group with externalizing disorders (ADHD). | The study was retrospective and had a relatively small sample size. It was conducted at a single, specialized headache clinic, which may have created a selection bias toward more severe cases. There was no control group of children with migraine but without psychiatric disorders for comparison. The potential impact of medication use on migraine characteristics was not investigated. |
| **Nahman-Averbuch et al. (2022)** | The study primarily focused on the differences in subjective sleep difficulties (insomnia symptoms) and objective sleep patterns between adolescents with and without migraine, and how these factors related to functional disability. | Observational, case–control study. Analyses: Independent t-tests were used to compare groups on sleep and disability measures. | Adolescents with migraine reported significantly greater subjective sleep difficulties (higher insomnia scores) compared to healthy controls. While not statistically significant, the migraine group also showed a trend towards more objective sleep disruption (e.g., more time awake after falling asleep) based on actigraphy. Within the migraine group, a higher degree of perceived sleep difficulty was strongly correlated with greater functional disability. There were no significant differences in anxiety or depression levels between the two groups. Although differences in brain connectivity were found, these were not explained or mediated by sleep difficulties, suggesting that sleep problems and brain alterations may be independent factors in migraine. | he sample size was small (20 participants per group). The cross-sectional design prevents determining if sleep problems contribute to migraine or vice versa. The exclusion of participants with psychiatric conditions like depression or anxiety may limit how representative the sample is of the general pediatric migraine population. |

**Table S1 (part 2 of 2).** Primary studies that met the inclusion criteria (continued from part 1).

| | | | | |
|---|---|---|---|---|
| **Natalucci et al. (2019a)** | Alexithymia: The ability to identify and describe one's own emotions. Metacognition: The ability to reflect on one's own thought processes. Theory of Mind (ToM): The ability to understand the mental states of others. | Observational, case–control study. Analyses: Non-parametric tests (Mann–Whitney, Kruskal–Wallis) were used to compare scores between the groups. | Children with migraine scored significantly higher on measures of alexithymia than healthy controls, specifically showing more "Difficulty Identifying Feelings" and "Difficulty Describing Feelings." There were no significant differences between the migraine group and the control group on measures of metacognition or Theory of Mind. The study confirms a link between pediatric migraine and alexithymia but does not support an association with deficits in metacognition or ToM. | The sample size was relatively small, which limited the analysis of how headache characteristics (e.g., frequency) might relate to the findings. The sample was recruited from a single specialist center, which could create selection bias. The study relied on self-report measures, which can be influenced by social desirability. |
| **Natalucci et al. (2019b)** | Alexithymia: The difficulty in identifying and describing one's own emotions. Metacognition: Beliefs about one's own thought processes, such as worry. Theory of Mind (ToM): The ability to understand the mental states and intentions of others. | An observational, comparative study of two distinct clinical groups. Analyses: T-tests were used to compare the mean scores on the psychological questionnaires between the two groups. | There were no significant differences between the migraine group and the internalizing disorders group in their levels of alexithymia or their Theory of Mind (ToM) performance. The group with internalizing disorders scored significantly higher on measures of dysfunctional metacognition, specifically on negative beliefs about worry (NMW) and cognitive monitoring (CM), compared to the migraine group. The findings suggest that while both groups may show similar difficulties with emotional awareness, maladaptive metacognitive beliefs are more characteristic of primary internalizing disorders than of migraine in this age group. | The study did not include a healthy control group for comparison. The authors suggest that future studies should compare different headache types and include patients with a dual diagnosis (migraine + internalizing disorder). |
| **Nita et al. (2020)** | The study focused exclusively on objective sleep architecture as measured by PSG. It did not use subjective questionnaires for cognitive or psychological states, but instead measured parameters like sleep stage percentages, arousals, and sleep latency to identify sleep disorders. | Prospective, observational, case–control study. Analyses: T-tests and other statistical methods were used to compare PSG parameters between the migraine and control groups. | Compared to the control group, the children with migraine showed a significantly altered sleep architecture, characterized by: A decreased percentage of REM sleep. An increased percentage of N1 sleep (the lightest stage of sleep). More frequent arousals from sleep. Shorter sleep latency (they fell asleep more quickly). The odds of having a PSG-defined sleep disorder were 5.6 times higher in the migraine group compared to the control group. | The sample size was very small (18 patients and 16 controls). The control group was not healthy; they had other headache types and emotional disorders, which could have influenced their sleep patterns and confounded the results. The PSG protocol used did not allow for the evaluation of other potential sleep issues like sleep apnea or periodic limb movement disorder. |

**Table S1 (part 2 of 2).** Primary studies that met the inclusion criteria (continued from part 1).

| | | | | |
|---|---|---|---|---|
| **Operto et al. (2018)** | Parenting Stress: The level of stress parents experience in their role as caregivers. Adolescent Emotional/Behavioral Problems: Specifically, internalizing problems (e.g., anxiety, depression, somatic complaints) and externalizing problems. | Observational, case–control study. Analyses: The Mann–Whitney U test was used to compare scores between the two groups. Spearman's correlation was used to assess relationships between parenting stress and child behavior within the headache group. | There was no significant difference in overall parenting stress levels between the parents of adolescents with headaches and the parents of healthy controls. However, adolescents with headaches had significantly more parent-reported internalizing problems, particularly affective problems, anxiety, and somatic complaints, compared to the control group. Within the headache group, a strong correlation was found: the more emotional and behavioral problems an adolescent had, the higher the level of stress their parents reported. Higher headache frequency was also correlated with higher parenting stress. | The sample size was small (35 patients and 23 controls), limiting the generalizability of the findings. The study's correlational design cannot determine causality (e.g., whether child behavior problems cause parental stress or vice-versa). The small sample did not allow for separate analyses of different headache subtypes. |
| **Operto et al. (2022)** | Social Cognition: The ability to understand others' mental states (TM) and identify emotions from faces (ER). Executive Functions. Non-verbal intelligence. | Cross-sectional, observational study comparing three groups. Analyses: Non-parametric tests (Kruskal–Wallis, Mann–Whitney U) were used to compare test scores among the groups. Spearman correlation was used to assess relationships between cognitive domains. | Both the migraine group and the epilepsy group performed significantly worse than the healthy control group on tests of Emotion Recognition and Theory of Mind. The migraine and epilepsy groups performed similarly to each other on these social cognition tasks, with no significant difference between them. Both clinical groups also showed poorer executive function performance compared to the controls. There were no significant differences in non-verbal intelligence among the three groups. In the migraine group, poorer social cognition skills were significantly correlated with poorer executive functions. | The sample sizes for the clinical groups were relatively small. The cross-sectional design does not provide information on how these skills develop over time. The study did not evaluate the potential impact of anti-seizure medication on the cognitive performance of the epilepsy group. |
| **Orr et al. (2017)** | The study assessed the statistical association between having a migraine diagnosis and the presence of a diagnosed mood disorder (e.g., depression), a diagnosed anxiety disorder, and adolescents' own perceived mental health. | Cross-sectional analysis of data from six cycles of a large, population-based survey. Analyses: Multivariate logistic regression was used to model the relationship between migraine and the mental health variables, adjusting for demographic factors. | A strong and consistent association was found between migraine and mental health disorders. Adolescents with a diagnosed mood disorder were approximately 3 to 4.6 times more likely to also have migraine. Adolescents with a diagnosed anxiety disorder were approximately 1.9 to 4.2 times more likely to have migraine. Conversely, adolescents who reported high perceived mental health were significantly less likely to have migraine. | The study relied on self-reported diagnoses for both migraine and psychiatric conditions, which carries a risk of misclassification bias. The cross-sectional design cannot determine causality (e.g., whether migraine leads to anxiety, or vice versa). The survey data did not differentiate between episodic and chronic migraine. |

**Table S1 (part 2 of 2).** Primary studies that met the inclusion criteria (continued from part 1).

| | | | | |
|---|---|---|---|---|
| **Philipp et al. (2019)** | The study focused on the burden and impact of primary headaches, measuring: Health-Related Quality of Life (HrQoL). Impact on school (e.g., missed days). Restriction of daily activities. | Cross-sectional, nationwide, school-based study. Analyses: Descriptive statistics were used for prevalence. Logistic regression and general linear models were used to analyze the associations between headache type, sociodemographic factors, and measures of burden. | The 1-year prevalence of any headache was very high at 75.7%. The most common specific diagnosis was undifferentiated headache (UdH) at 26.1%, followed by migraine (24.2%) and TTH (21.6%). Headaches were associated with a significant burden, with 42% of affected students reporting restrictions in daily activities and 50% using acute medication. Health-related quality of life was significantly lower for all headache types (migraine, TTH, H15+) compared to controls, with the exception of UdH, which showed no significant impact. A clear gradient of burden was observed, with the most severe impact in those with frequent headaches (H15+), followed by migraine, then TTH. The 1-year prevalence of any headache was very high at 75.7%. The most common specific diagnosis was undifferentiated headache (UdH) at 26.1%, followed by migraine (24.2%) and TTH (21.6%). Headaches were associated with a significant burden, with 42% of affected students reporting restrictions in daily activities and 50% using acute medication. Health-related quality of life was significantly lower for all headache types (migraine, TTH, H15+) compared to controls, with the exception of UdH, which showed no significant impact. A clear gradient of burden was observed, with the most severe impact in those with frequent headaches (H15+), followed by migraine, then TTH. | The study had a high non-participation rate (55.7%), which creates a risk of selection bias. The cross-sectional design does not allow for determining causality. The reliance on self-report questionnaires may be subject to recall bias. The exclusion of children under 10 may have led to an underestimation of the prevalence of UdH. |

**Table S1 (part 2 of 2).** Primary studies that met the inclusion criteria (continued from part 1).

| | | | | |
|---|---|---|---|---|
| **Rabner et al. (2018)** | The study compared the severity of several psychosocial factors across the three headache diagnoses: Sleep disturbance. Anxiety and depressive symptoms. Functional disability. | Retrospective chart review of patients from a multidisciplinary headache clinic. Analyses: One-way ANOVA was used to compare scores across the three headache groups. Hierarchical linear regression was used to identify predictors of sleep disturbance. | Sleep disturbance was significantly greater in patients with TTH and NDPH compared to patients with migraine. Across all headache types, greater sleep disturbance was significantly associated with higher levels of functional disability, anxiety, and depression. After controlling for all other factors, a diagnosis of NDPH, older age, higher disability, and higher depression scores remained the strongest significant predictors of greater sleep disturbance. | The study was retrospective and conducted at a single specialty clinic, which may limit generalizability. Sleep was measured only by parent-report, not by the child's self-report or objective measures like actigraphy. The potential confounding effect of medication use on sleep was not controlled for. The sample was predominantly female and Caucasian. |
| **Rizvi et al. (2024)** | The study investigated whether the severity of anxiety and depressive symptoms at an initial clinic visit could predict changes in migraine outcomes (headache frequency and disability) at a subsequent follow-up visit. | Prospective longitudinal clinical cohort study. Analyses: Multivariable linear regression models were used to estimate the association between baseline mental health symptoms and changes in migraine outcomes, controlling for covariates like age, sex, and treatment. | Contrary to the study's hypothesis and common clinical belief, baseline levels of anxiety and depressive symptoms did not significantly predict the change in either headache frequency or migraine-related disability at the follow-up visit. In exploratory analyses, changes in anxiety or depression symptoms over time were also not significantly associated with changes in migraine outcomes. | A significant number of potential participants were excluded due to missing data, creating a risk of sampling bias. The study was conducted at a single tertiary care center, which may limit the generalizability of the findings. The time interval between the baseline and follow-up visits was inconsistent across patients, reflecting real-world practice rather than a standardized study design. The study may have been underpowered to detect a weak (but potentially still meaningful) association. |
| **Romano et al. (2020)** | The study assessed the frequency of several psychiatric comorbidities, with a primary focus on: Anxiety and depression. Attention-deficit/hyperactivity disorder (ADHD). Learning disabilities. Tics and obsessive-compulsive disorder (OCD). | Case–control study. Analyses: Chi-square tests and logistic regression were used to compare the prevalence of comorbidities between the headache and control groups. | A strong, statistically significant association was found between primary headaches and internalizing disorders. 27% of children in the headache group had anxiety and/or depression, compared to only 8.3% of children in the control group. The odds of having anxiety or depression were 4.2 times higher for children with primary headaches than for their healthy peers. In contrast, there were no significant differences found between the two groups in the prevalence of ADHD, learning disabilities, tics, or OCD. | The assessment of psychiatric comorbidities was based on direct interviews rather than standardized, validated questionnaires, which may limit the reliability of the findings. The study used the older ICHD-II criteria for headache diagnosis. The cross-sectional design does not allow for determining causality. |

**Table S1 (part 2 of 2).** Primary studies that met the inclusion criteria (continued from part 1).

| | | | | |
|---|---|---|---|---|
| **Sciruicchio et al. (2023)** | The study focused on the clinical symptom of osmophobia and its correlation with psychological factors, including anxiety and depression symptoms, pain catastrophizing (a negative cognitive response to pain), and headache-related disability. | A multicenter, observational, cross-sectional study. Analyses: Chi-square tests and MANOVA were used to compare clinical and psychological variables between the osmophobic and non-osmophobic groups. | Osmophobia was present in 28.8% of children with primary headaches, with the highest prevalence in those with migraine (35%). The presence of osmophobia was associated with a more severe headache profile, including longer headache duration, higher pain intensity, and a strong association with allodynia (pain from non-painful stimuli). In a subgroup analysis of migraine patients, those with osmophobia reported significantly higher levels of disability, anxiety, depression, and pain catastrophizing compared to migraine patients without osmophobia. | The number of patients with tension-type headache was much smaller than the number with migraine, which is a common bias in specialty headache clinics. A simple interview question was used to identify osmophobia, rather than a detailed, structured questionnaire. The detailed psychological assessments were conducted only on a subgroup of patients with migraine, not the entire sample. |
| **Sciruicchio et al. (2019)** | The study focused on pain catastrophizing, which is a negative cognitive and emotional response to pain, and its relationship with anxiety and depression symptoms, migraine-related disability and general quality of life, and physical symptoms of central sensitization (e.g., allodynia). | Observational, cross-sectional study. Analyses: ANOVA was used to compare scores between the episodic and chronic migraine groups. Pearson correlation was used to assess the relationship between pain catastrophizing and other clinical variables. | There was no significant difference in the level of pain catastrophizing between children with episodic migraine and those with chronic migraine. Pain catastrophizing was not correlated with headache frequency or migraine-specific disability (PedMIDAS). However, higher levels of pain catastrophizing were significantly correlated with poorer general quality of life, higher levels of anxiety and depression, and more severe signs of central sensitization (allodynia and pericranial tenderness). | The study was conducted in a single tertiary headache center, so the results may not be generalizable to the broader pediatric migraine population. The cross-sectional design cannot determine if pain catastrophizing is a risk factor for migraine chronification over time. The Italian version of the pain catastrophizing scale used was not yet formally validated at the time of the study. |
| **Shimomura et al. (2021)** | The study focused on emotional and behavioral problems (e.g., emotional symptoms, conduct problems, peer problems) and whether a child's baseline psychological profile could predict how quickly they would respond to headache treatment. | Prospective cohort study. Analyses: The Mann-Whitney U test and Fisher's exact test were used to compare SDQ scores between the headache groups and between fast vs. slow treatment responders. | At baseline, patients with migraine showed significantly more overall difficulties and specifically more emotional symptoms on the SDQ compared to patients with TTH. The baseline psychological profile was associated with the time to treatment response, but this differed by headache type: Among migraine patients, those who took longer to improve (>3 months) had significantly more peer problems at baseline. Among TTH patients, those who took longer to improve (>3 months) had significantly fewer conduct problems at baseline. | The follow-up analysis had a 27% attrition rate. The treatments provided were not standardized according to a strict protocol. All patients were treated by a single physician, which could limit the generalizability of the findings. The number of patients in the TTH group was small. |

**Table S1 (part 2 of 2).** Primary studies that met the inclusion criteria (continued from part 1).

| | | | | |
|---|---|---|---|---|
| **Öztop et al. (2016)** | The study assessed the prevalence of formal psychiatric disorders and the severity of anxiety and depressive symptoms. It also measured the impact of migraine on quality of life. | Observational, case–control study. Analyses: T-tests and Mann–Whitney U tests were used to compare scores between the migraine and control groups. Correlation analyses were used to examine the relationships between variables within the migraine group. | A formal psychiatric diagnosis was made in 40% of the children and adolescents with migraine. The migraine group had significantly higher depression scores compared to the healthy control group. There was no significant difference in anxiety scores between the two groups. Quality of life was significantly poorer in the migraine group compared to the controls. Within the migraine group, poorer quality of life was correlated with higher pain severity and greater disability. | The sample size was small (35 patients and 35 controls). The study did not assess migraine frequency, a key clinical variable. The cross-sectional design does not allow for determining causality between migraine and the psychological factors. |
| **Strong et al. (2021)** | The study documented the prevalence of comorbid psychological conditions, primarily anxiety (reported by 19% of patients) and depression (10%). It also noted the potential for negative impacts on school performance and social functioning due to the persistent nature of the pain. | Retrospective chart review. Analyses: Descriptive statistics were then used to analyze the collected data. | NDPH was diagnosed in 14% of the clinic's total headache population. The patients were predominantly female (78%) and white (73%), with a median age of 14.8 years. The median pain intensity was 6/10. A vast majority of patients reported migrainous features, including photophobia (85%), phonophobia (85%), and reduced activity levels (88%). The condition was highly refractory to treatment, with 56% of patients having failed at least one abortive medication and 36% developing comorbid medication overuse headache. Regarding psychological comorbidities, anxiety was the most commonly reported, present in 19% of patients, followed by depression (10%) and ADD/ADHD (6%). Both anxiety and depression were reported more frequently in female patients than in male patients. | The study's generalizability is limited because it was conducted at a single tertiary care center, meaning that the patient population may not be representative of the general population. The retrospective nature of the study led to incomplete data in some records, which could have affected the accuracy of the findings. The study was unable to determine if medication overuse preceded or followed the onset of NDPH. The family history and comorbidity data collected were not sufficiently detailed. |
| **Syed & Mullick (2025)** | The study assessed for a range of psychiatric disorders, including anxiety disorders (e.g., separation anxiety, phobias), depressive disorders, behavioral disorders (e.g., ODD, conduct disorder), PTSD, OCD, and self-harm. | Descriptive, cross-sectional study. Analyses: Chi-square tests and logistic regression were used to analyze the relationships between headache characteristics and psychiatric co-morbidities. | Psychiatric co-morbidities were identified in 39.7% of the participants. The most common headache type was Tension-Type Headache (TTH) (62.9%). Anxiety disorders (19.9%) and depressive disorders (12.6%) were the most prevalent psychiatric conditions. A significant association was found between higher headache frequency and the presence of psychiatric co-morbidities (p=0.020). Headache frequency was identified as a minor but significant | The final sample size (151) was smaller than the target (216) due to the COVID-19 pandemic, which may have reduced statistical power. The cross-sectional design prevents conclusions about causality. The lack of a control group limits comparison with the general pediatric population. Potential for reporting bias exists, as data was self- or caregiver-reported, and genetic/epigenetic factors were not assessed. |

**Table S1 (part 2 of 2).** Primary studies that met the inclusion criteria (continued from part 1).

| | | | | |
|---|---|---|---|---|
| | | | predictor of having a psychiatric disorder (OR = 1.06). | |
| **Tarantino et al. (2015)** | The study focused on anxiety and somatization. This included sub-scales for generalized anxiety, social anxiety, separation anxiety, school anxiety, somatic symptoms, and hypochondria. | Cross-sectional, comparative study. Analysis: Analysis of variance (ANOVA), t-tests, and Pearson correlation analyses. | Children with MERS reported significantly higher levels of total anxiety (p=0.001) and total somatization (p=0.024) compared to those without MERS. Within the MERS group, a low frequency of headache attacks was associated with significantly higher levels of separation anxiety (p=0.034). No significant differences were found in the "Hypochondria" subscale between the groups. | The findings are from a single tertiary headache center and may not be generalizable to the wider population. The reporting of MERS was retrospective, introducing a risk of recall bias. The psychological assessment was based on self-report questionnaires, which can screen for symptoms but cannot provide a formal psychiatric diagnosis. Since all participants had migraine, it is difficult to attribute the psychological differences solely to MERS. |
| **Tarantino et al. (2017)** | The study investigated attachment styles (secure, avoidant, ambivalent, disorganized/confused) and psychological symptoms, including anxiety, depression, and somatization. | Analytical observational case–control study (using internal comparison groups). Analysis: Chi-square tests, one-way and two-way ANOVAs, and t-tests with Bonferroni's correction for post hoc analysis. | A significant relationship was found between attachment style and migraine features. The ambivalent attachment style was most common among patients with high-frequency attacks (51%) and severe pain (50%). Patients with an ambivalent attachment style also showed significantly higher levels of anxiety, depression, and somatization. Within the ambivalent group, a high attack frequency was associated with higher anxiety levels. | The study's sample was recruited from a single tertiary headache center, which may limit the generalizability of the findings. The psychological assessment tools are self-report screening instruments and cannot provide a formal psychiatric diagnosis. The study did not assess the attachment style of the caregivers, nor did it include a healthy control group for comparison. |

**Table S1 (part 2 of 2).** Primary studies that met the inclusion criteria (continued from part 1).

| | | | | |
|---|---|---|---|---|
| **Togha et al. (2023)** | The study's primary focus was on the impact of headache on quality of life. It also noted the common association of TTH with triggers like stress and anxiety. | Cross-sectional survey. Analysis: Chi-square test and independent two-sample t-test. | The prevalence of TTH was 32.1% among the participants. The mean age of the TTH group was significantly higher than the control group. Quality of life was significantly lower for individuals with TTH compared to controls, and it decreased as headache frequency increased. Higher headache frequency was also associated with more missed school days and greater use of analgesic medication. The study findings shed light on the profound impact that tension headaches have on various aspects of individuals' daily lives. As the number of headache days escalates, participants reported an increased number of absences from both school and work. Consequently, this has led to a rise in the number of parental leave days taken by caregivers in order to address their children's condition. | The article does not contain a dedicated limitations section. However, potential limitations inherent in the design include reliance on self-report questionnaires and a cross-sectional design that cannot establish causality. |
| **Torres-Ferrus et al. (2019)** | The study used the Strengths and Difficulties Questionnaire (SDQ) to screen for psychological issues across five scales: emotional symptoms, conduct problems, hyperactivity/inattention, peer relationship problems, and prosocial behavior. It also gathered data on previously diagnosed mental health problems. | Cross-sectional, observational study. Analysis: Chi-square tests, Student's t-test, and logistic regression models were used to compare groups and identify risk factors associated with headache. | The prevalence of recurrent headache was 30.5%. Headache was significantly associated with unhealthy lifestyle factors (e.g., poor sleep, smoking, less physical activity) and comorbidities like allergies, chronic pain, and mental health problems. Adolescents with headache had significantly worse scores on the emotional, conduct, hyperactivity, and peer relationship scales of the SDQ. 44.1% of those with headache reported some degree of headache-related disability. | Headache diagnosis was based on a self-report survey, not a clinical interview, which may affect prevalence estimates. The reliance on self-reported information on lifestyle habits might not be entirely reliable. The sample had lower participation from rural areas and students who left school after age 16, potentially limiting the generalizability of the findings. |
| **Uçar et al. (2020)** | The study focused on irritability, anxiety, and emotional/behavioral problems (including conduct problems, hyperactivity, peer problems, and emotional symptoms). | Single-center, cross-sectional, case–control study. Analysis: T-tests, chi-square tests, Pearson's correlation, MANCOVA/ANCOVA to control for confounders, and logistic regression. | Adolescents with migraine showed significantly higher self- and parent-reported irritability scores compared to healthy controls ($p\<0.001$). The migraine group also had significantly higher levels of anxiety and emotional problems. In the migraine group, higher irritability was moderately correlated with higher levels of anxiety, emotional problems, and attention deficit/hyperactivity problems. Self-reported irritability and emotional | The study had a modest sample size. It did not include formal psychiatric evaluations for participants with high scores on the screening tools. The cross-sectional design does not allow for determining causality. Parent-report scales were completed by only one parent, not necessarily the same one for all participants. |

**Table S1 (part 2 of 2).** Primary studies that met the inclusion criteria (continued from part 1).

| | | | | |
|---|---|---|---|---|
| | | | problems were significant predictors of migraine. | |
| **Uyar Cankay & Besenek (2021)** | The study screened for 12 psychiatric symptom clusters, including depression, anxiety, aggression, irritability, attention problems, and somatic symptoms. It also provided formal diagnoses for conditions like Major Depressive Disorder (MDD) and Generalized Anxiety Disorder (GAD). | Cross-sectional, case–control study. Analysis: T-tests, ANOVA, Mann–Whitney U, Kruskal–Wallis, and chi-square tests for group comparisons. Pearson and Spearman tests for correlation analysis. | All psychiatric symptom scores on the CCSM-5 (except psychotic symptoms) were significantly higher in the chronic migraine group. 58% of adolescents with chronic migraine received at least one psychiatric diagnosis (most commonly MDD and GAD). However, having a psychiatric diagnosis did not significantly affect headache severity (VAS) or disability (PedMIDAS). Higher scores for attention problems were positively correlated with both greater headache severity and disability. | The study had a relatively small sample size. The psychiatric screening tool (CCSM-5) only assessed symptoms over the previous two weeks. The cross-sectional design prevents the determination of causality. Clinical and environmental risk factors were not assessed in the control group, limiting direct comparisons of these factors' roles in migraine pathophysiology. |
| **Villa et al. (2016)** | The study focused on various components of visual attention, including selective attention, sustained attention, alternate attention, mental flexibility, and psychomotor speed. | Comparative, case–control study. Analysis: One-way analysis of variance (ANOVA) with the post-hoc Tukey test was used to compare the means of the three groups. | Although all groups performed within the normal range, the untreated migraine group performed significantly worse on several visual attention tests compared to both the control group and the group receiving prophylaxis. The migraine prophylaxis group's performance was similar to that of the healthy control group, suggesting that effective prophylactic treatment might reverse or normalize these attention deficits. | The sample sizes for the groups were small, which limited the statistical power to analyze differences between migraine subtypes (with/without aura) or between different prophylactic medications. The study was cross-sectional, meaning the same children were not assessed before and after starting treatment. |

**Table S1 (part 2 of 2).** Primary studies that met the inclusion criteria (continued from part 1).

| | | | | |
|---|---|---|---|---|
| **von Gontard et al. (2019)** | The study used the SDQ to assess psychological symptoms across five scales: emotional symptoms, conduct problems, hyperactivity, peer problems, and prosocial behavior. These were also combined into internalizing and externalizing problem scores. | Population-based, cross-sectional study.<br>Analysis: Chi-square tests, t-tests, ANOVA, and regression analysis. | Primary headache was not significantly associated with any type of incontinence but was significantly associated with constipation. Children with primary headache had significantly more internalizing problems, while children with incontinence had significantly more externalizing problems (especially conduct problems). Primary headache was a significant predictor of internalizing symptoms, whereas constipation and fecal incontinence were predictors of externalizing symptoms. | The study had a modest participation rate (53.2%). Data was collected solely through parental questionnaires, without direct clinical examination of the children. There was a risk of underreporting sensitive issues like incontinence by parents. Constipation was assessed with a single question. |
| **Wagner et al. (2015)** | The study investigated the prevalence of diagnosed comorbidities based on ICD-9-CM codes, categorized as:<br>- Mental Health Comorbidities: Depression, anxiety, suicidal ideation, substance misuse, psychosis, bipolar disorder, etc.<br>- Neurodevelopmental Comorbidities: ADHD, autism spectrum disorder, intellectual disability, and cognitive disorder. | Retrospective, population-based case–control study using surveillance data. Analysis: ANOVA and chi-square tests for group comparisons. Polytomous logistic regression was used to calculate odds ratios (OR) for the association between the primary condition (epilepsy or migraine) and comorbidities. | Comorbidities were highly prevalent in the epilepsy group (29.7% mental health, 30.8% neurodevelopmental). Compared to the LEF group, the adjusted odds of having a mental health comorbidity were significantly higher for both the epilepsy group (OR=2.20) and the migraine group (OR=1.60). The risk for neurodevelopmental comorbidities was substantially higher in the epilepsy group than in the migraine or LEF groups. The current study shows that the proportion of mental health comorbidities is comparable in children aged 6 to 12 years with epilepsy (17.9%) and migraine (13.7%). However, adolescents with epilepsy are more likely to have a mental health comorbidity than adolescents with migraine. This is supported by significantly elevated odds of any mental health comorbidity (and each specific mental health comorbidity) in adolescents with epilepsy compared with the migraine control group after adjusting for neurodevelopmental disorders and demographic and clinical covariables. | The study relied on administrative billing data (ICD-9-CM codes), which may contain coding errors or variations and might not accurately reflect clinical diagnoses. The data lacks corroboration from medical records or patient reports, potentially introducing information bias. Differences between the ICD-9 and DSM classification systems could also affect the accuracy of mental health diagnoses. |

**Table S1 (part 2 of 2).** Primary studies that met the inclusion criteria (continued from part 1).

| | | | | |
|---|---|---|---|---|
| **Walter et al. (2021)** | The study investigated the prevalence of diagnosed depression and anxiety as comorbidities by searching for their respective ICD codes in the patient records. | Cross-sectional, descriptive, secondary data analysis (retrospective chart review). Analysis: Chi-square test for comparing categorical variables and Student's t-test for comparing the mean number of comorbidities between the obese and nonobese groups. | Of 648 children with migraine, 26.4% were classified as obese. There were no significant differences between the obese and nonobese groups regarding the prevalence of anxiety or depression. However, the prevalence of obstructive sleep apnea syndrome (OSAS) was significantly higher in the obese migraine group (20% vs. 9.9%). Obese children with migraine also had a significantly higher mean number of total comorbidities compared to their nonobese peers. There was no significant difference in anxiety or depression between the children and adolescents with migraine who were classified as obese or nonobese. However, approximately 40% of obese migraineurs and 34% of nonobese migraineurs were diagnosed with either depression or anxiety. | The study used a cross-sectional design, which cannot establish causality. It relied on diagnostic codes from medical records, which may not accurately capture the true prevalence of comorbidities and were not confirmed with validated screening tools. Data on medications, a potential confounder, was not collected. The use of a single, rural, clinic-based sample limits the generalizability of the results. |
| **Yavuz et al. (2023)** | The study assessed a range of psychiatric symptoms, including depression, anxiety, sleep problems, attention-deficit/hyperactivity, and general emotional and behavioral problems. | Case–control study. Analysis: T-tests, Mann–Whitney U tests, and chi-square tests were used for group comparisons. ANOVA was used for comparing subgroups, and Spearman's correlation was used to assess relationships between variables. | The psychiatric clinic group reported a significantly higher frequency of headaches compared to the control group. Within the clinic group, headache frequency was positively correlated with depression, anxiety, and sleep problems. Also, within the clinic group, children with a migraine diagnosis had significantly higher scores for depression, anxiety, and emotional problems. Migraine was associated with a lower quality of life in both groups. | The study relied on psychometric scales, which are not sufficient for formal psychiatric diagnoses. The cross-sectional design does not allow for determining a cause-and-effect relationship. There was a significant gender imbalance between the study (more boys) and control (more girls) groups. The study took place during the COVID-19 pandemic, which could have affected the quality of life data for all participants. |
| **Miscioscia et al. (2022)** | The study focused on emotional experience (positive and negative affect), emotion regulation/awareness (e.g., verbal sharing, bodily awareness), and psychological adjustment (internalizing and externalizing symptoms). | Cross-sectional, case–control pilot study. Analysis: Mann–Whitney U tests for group comparisons and multiple linear regression to identify predictors of internalizing symptoms. | Compared to controls, the clinical group reported significantly higher negative affect and internalizing symptoms and lower bodily awareness of emotions. A higher frequency of headaches was associated with greater emotion regulation difficulties. For children with headaches, their internalizing symptoms were predicted by higher self-reported negative affect, higher parent-reported internalizing symptoms, and lower self-reported ability to verbally share emotions. | As a pilot study, the sample size was small, which limits generalizability and statistical power. The cross-sectional design does not allow for determining causality. The study relied on questionnaires, some of which were not validated for the Italian pediatric population. The clinical sample was from a single tertiary center. |

**Table S1 (part 2 of 2).** Primary studies that met the inclusion criteria (continued from part 1).

| | | | | |
|---|---|---|---|---|
| **Galli et al. (2017)** | The study focused on psychopathological symptoms, specifically internalizing problems (anxiety, depression, somatic complaints) and externalizing problems (rule-breaking, aggressive behavior), as measured by both parent and child reports. | Case–control study. Analysis: Chi-square tests, ANOVA, correlations, and independent-samples t-tests. | Headache showed familial recurrence, particularly on the paternal side. The headache group had significantly higher scores for psychopathology on both the CBCL (parent-report) and SAFA (self-report) compared to the control group, with notable differences in internalizing, externalizing, and total problem scores. However, there were no significant differences in psychopathology between the migraine and other headache subgroups. In the headache group, but not the control group, children's self-reported anxiety and depression correlated with their mothers' reports of internalizing symptoms. | The study was clinic-based, which may introduce a self-selection bias. Headache diagnoses in parents were based on questionnaires, not clinical examinations. The control group was not matched for environmental factors, which could be confounders. |
| **Onofri et al. (2022)** | The study focused on the diagnosis of learning disabilities (LD) and screened for comorbid psychopathologies, including anxiety disorders, depression, and obsessive-compulsive disorder (OCD). | Cross-sectional, comparative, clinic-based observational study. Analysis: Chi-square test and Wilcoxon signed-rank test. | The prevalence of LD in the headache sample (9.44%) was higher than in the general Italian population. The group with both headache and LD had a significantly higher prevalence of anxiety disorders compared to the group with only headache. In patients with chronic tension headache (CTH), co-occurring LD was associated with a much higher rate of school absences (50% vs. 22.2%). | The study is cross-sectional and observational, so it cannot establish causality. The sample was recruited from a single, specialized Headache Center, which may not be representative of the general population. School absence is a limited proxy for overall quality of life. |
| **Şentürk et al. (2023)** | The study used the SDQ to assess for behavioral and emotional problems across five subscales: emotional symptoms, conduct problems, hyperactivity/inattention, peer problems, and prosocial behavior, as well as a total difficulties score. | Cross-sectional, observational study. Analysis: T-tests, Mann-Whitney U test, chi-square test, and Spearman correlation. | The overall prevalence of primary headache was 59.8%. The migraine group had significantly higher pain severity (VAS) and disability (PedMIDAS) scores compared to the TTH group. Psychologically, the migraine group had significantly higher (worse) scores for emotional problems and total difficulties on the SDQ compared to both the TTH and no-headache groups. | The cross-sectional design does not allow for determining causality. The study relied on self-reported data from children, which can be subject to recall bias. It did not identify children with co-occurring migraine and TTH ("mixed headache"). The single-center design (one boarding school) may limit generalizability. |
| **Erdoğan et al. (2025)** | Daytime sleepiness (EDS) and academic performance. | Cross-sectional, community-based survey. Prevalence estimates and group comparisons; specific statistical tests not detailed in the abstract. | RLS 3.2%; headache 46.9%; TTH 17.7%; migraine 5.2%. ESS higher in RLS+ vs RLS−; RLS more frequent among adolescents with headache/migraine; higher ESS → lower academic success. | Cross-sectional (no causality), reliance on self-report; single-province sample; no objective sleep measures; exact subgroup counts and mean age not available in accessible text layer. |

**Table S2 (part 1 of 2).** Reviews and/or meta-analyses that met the inclusion criteria. *Note: Table S2 is presented in two parts; see part 2 for continuation.*

| Authors and Year | Title | Main Purpose of the Review | Research Question(s) of the Review | Methods | Number & Type of Studies Included |
|---|---|---|---|---|---|
| **Ayta et al. (2016)** | Quality of Life in Children and Adolescents With Primary Headache Disorders | To raise awareness about the significant impact of primary headache disorders on the quality of life (QoL) of children and adolescents by summarizing existing evidence. | How do primary headaches (migraine, TTH) affect the QoL (general, psychological, academic, social) of children and adolescents? | As a narrative review, the article does not specify a systematic search method. | The authors do not state the specific number of articles analyzed for the review. |
| **Basile, V. et al. (2024)** | Primary Headaches and Sleep Disorders: Review of Literature about Comorbidity in Children and Adolescents | To review the existing literature on the co-occurrence of primary headaches and sleep disorders in children and adolescents and to summarize the findings. | The study aimed to investigate the presence of comorbidity between primary headaches and sleep disorders in the pediatric population. | This was a systematic review following PRISMA guidelines. The authors searched the PubMed database for relevant studies published in the last 20 years and used specific inclusion and exclusion criteria to select articles for analysis. | The authors explicitly state that they included 12 studies in their final review, which collectively involved 16,474 subjects between the ages of 2 and 18. |
| **Dosi et al. (2015)** | Sleep and headache | To review the literature concerning the interaction and high rate of comorbidity between sleep disorders and primary headaches in children and adolescents. | The review addresses the nature of the relationship between sleep and headaches, the specific sleep disorders commonly associated with pediatric headaches, and the potential shared pathophysiological mechanisms. | Narrative review. The authors synthesize and discuss findings from previously published literature to provide a comprehensive overview of the topic. A systematic search protocol is not described. | The authors do not state a specific number of articles that were formally selected for analysis, but they base their discussion on a broad range of scientific literature. |
| **Dyb et al. (2015)** | Psychiatric Comorbidity in Childhood and Adolescence Headache | To summarize clinical and epidemiological evidence for psychiatric comorbidity in children and adolescents with headaches and to describe potential pathways involved in the development of these co-occurring conditions. | 1. What is the evidence for psychiatric comorbidity in pediatric headaches? 2. What pathways are involved in the development of these comorbidities? 3. What clinical elements are helpful for assessment and treatment planning? | This is a narrative review. The authors synthesize findings from a wide range of clinical and epidemiological studies to build a conceptual model and provide clinical recommendations. A systematic search protocol is not described. | The authors do not state a specific number of articles analyzed, but they base their discussion on a broad review of the existing literature. |
| **Gelfand (2015)** | Psychiatric comorbidity and paediatric migraine: examining the evidence | To critically review the evidence for the commonly assumed association between psychiatric comorbidities (like depression and anxiety) and pediatric migraine. | Is there strong evidence for an association between psychiatric disorders and migraine in children and adolescents? | This is a narrative review. The author synthesizes and critically appraises findings from the existing literature, including systematic reviews and population-based studies. A systematic search protocol is not described. | The author does not state a specific number of articles analyzed but bases the discussion on a critical appraisal of the existing literature. |

**Table S2 (part 1 of 2).** Reviews and/or meta-analyses that met the inclusion criteria. *Note: Table S2 is presented in two parts; see part 2 for continuation.*

| | | | | | |
|---|---|---|---|---|---|
| **Genizi et al. (2017)** | Primary Headaches and School Performance—Is There a Connection? | To review the literature on the epidemiological and pathophysiological connections between primary headaches and school functioning in children and adolescents, and to provide a clinical approach. | Is there a connection between primary headaches and learning disabilities (LD)? What are the potential mechanisms (psychosocial or anatomical)? What is the clinical relevance for assessment? | This is a narrative review. The authors synthesize and discuss findings from previous clinical and population-based studies to explore the relationship between headaches and school performance. A systematic search protocol is not described. | The authors do not state a specific number of articles analyzed, but base their discussion on a critical review of existing studies on the topic. |
| **Eidlitz Markus & Toldo (2018)** | Obesity and Migraine in Childhood | To review the literature on the epidemiology and pathophysiology of pediatric obesity and migraine, the potential mechanisms linking them, and the effects of treatment. | Is there an association between obesity and migraine in the pediatric population? What are the shared pathogenic mechanisms? What is the impact of obesity treatments on migraine? | This is a narrative review. The authors synthesize and discuss findings from epidemiological studies, pathophysiological research, and clinical trials to provide a comprehensive overview of the topic. | The authors do not state a specific number of articles analyzed, but base their discussion on a broad review of the existing scientific literature. |
| **Falla et al. (2022)** | Anxiety and Depressive Symptoms and Disorders in Children and Adolescents With Migraine A Systematic Review and Meta-analysis | To determine if there is an association between internalizing symptoms and disorders and migraine in children and adolescents. | Is there an association between internalizing symptoms/disorders and a migraine diagnosis? Are such symptoms associated with worse migraine outcomes (e.g., higher frequency)? Do internalizing symptoms represent a risk factor for developing migraine? | A systematic review and meta-analysis were conducted following PRISMA guidelines. A search of four databases (MEDLINE, Embase, PsycINFO, CINAHL) was performed up to March 2022. The quality of the studies was assessed using the Newcastle–Ottawa Scales. | 80 observational studies (case–control, cohort, cross-sectional) were included. Of these, 51 were used for the meta-analyses. |
| **Langdon et al. (2020)** | Pediatric Migraine and Academics | To explore these several variables contributing to learning and school functioning in children and adolescents with headache and migraine. | The review explores the relationships between pediatric migraine and academics by investigating several key areas: The link between migraine, ADHD, and learning disabilities. The role of psychosocial factors such as sleep disorders, psychiatric comorbidities (e.g., anxiety, depression), adverse childhood experiences (e.g., trauma, bullying), and perfectionism. | This article is a narrative literature review. It synthesizes and summarizes findings from previously published research to provide an overview of the current understanding of how migraine affects academics in pediatric patients. | The review does not specify the total number of included articles. It draws upon a wide range of study types. |
| **O'Brien & Slater (2016)** | Comorbid Psychological Conditions in Pediatric Headache | To summarize the literature on the prevalence of comorbid psychological conditions, such as anxiety and depression, in children and adolescents with chronic daily headaches (CDH). | What is the prevalence of psychiatric conditions (anxiety, depression, ADHD) in youth with chronic headaches? What are the barriers to identifying these comorbidities? What screening tools are available for this population? | This article is a narrative literature review that synthesizes findings from previous studies on the epidemiology and diagnosis of psychological comorbidities in pediatric headache. | The total number of studies is not specified, but the review cites various sources, including prevalence studies and articles on diagnostic tools. |

**Table S2 (part 1 of 2).** Reviews and/or meta-analyses that met the inclusion criteria. *Note: Table S2 is presented in two parts; see part 2 for continuation.*

| | | | | | |
|---|---|---|---|---|---|
| **Paolino et al. (2015)** | Headache and ADHD in Pediatric Age: Possible Physiopathological Links | To analyze the complex and often contradictory literature on the association between primary headache and Attention-Deficit/Hyperactivity Disorder (ADHD) in children, and to explore potential shared underlying mechanisms. | The review investigates the evidence for a link between headache and ADHD and explores three main potential shared pathophysiological pathways: Dopaminergic system dysfunction. Brain iron deficiency. Sleep disturbances. | This article is a narrative literature review that synthesizes findings from epidemiological, neurobiological, and pathophysiological studies. | The total number of studies is not specified. The review draws upon a range of sources, including epidemiological surveys, genetic studies, and neuroimaging research. |
| **Polese et al. (2022)** | Psychological disorders, adverse childhood experiences and parental psychiatric disorders in children affected by headache: A systematic review | To systematically review the literature on the association between headaches in young children (up to 12 years old) and various psychosocial risk factors, including the child's mental health, parental psychiatric issues, and adverse childhood experiences (ACEs). | The review sought to synthesize evidence on the link between childhood headaches and: Psychological symptoms in the child (internalizing and externalizing). Parental and family factors (e.g., family conflict, parental mental health). Adverse life events (e.g., bullying, school stress). | This article is a Systematic Review that followed the PRISMA guidelines. The authors conducted a formal search of three scientific databases to identify relevant studies published up to April 2021. | A total of 28 studies were included in the final analysis. The included articles consisted of cross-sectional, longitudinal, and case–control studies. |
| **Salem et al. (2018)** | ADHD is associated with migraine: a systematic review and meta-analysis | To systematically review the literature and conduct a meta-analysis to determine if a comorbid association exists between Attention-Deficit/Hyperactivity Disorder (ADHD) and primary headaches, particularly migraine and tension-type headache (TTH). | The primary research question was whether there is a statistically significant association between ADHD and different types of primary headaches (migraine and TTH). | This article is a Systematic Review and Meta-analysis that followed the PRISMA guidelines. The authors searched four scientific databases for studies published between 2000 and 2017. | 14 original articles were included in the systematic review. 11 of these studies had sufficient data to be included in the quantitative meta-analysis. The majority of the included studies were cross-sectional or retrospective. |
| **Shimomura (2022)** | Emotional Problems in Pediatric Headache Patients | To summarize current reports on emotional problems—including those related to personality, psychiatric disorders, and neurodevelopmental disorders—in children and adolescents with primary headaches. | The review synthesizes literature to answer: What personality traits are associated with pediatric headaches? What is the link between headaches and psychiatric conditions like anxiety and depression? What is the relationship between headaches and neurodevelopmental disorders like ADHD and ASD? | This article is a narrative literature review that discusses and synthesizes findings from various published studies, including meta-analyses and clinical research. | The total number of studies is not specified. The review cites a range of sources, including meta-analyses, cross-sectional studies, and longitudinal cohort studies. |

**Table S2 (part 1 of 2).** Reviews and/or meta-analyses that met the inclusion criteria. *Note: Table S2 is presented in two parts; see part 2 for continuation.*

| | | | | | |
|---|---|---|---|---|---|
| **Guidetti e al. (2019)** | Migraine in childhood: an organic, biobehavioral, or psychosomatic disorder? | To analyze recent scientific findings on the neurological, psychological, and environmental factors that contribute to the cause and maintenance of migraine in children and adolescents. | The review explores the multifactorial nature of pediatric migraine by investigating: What are the known biological, neural, and genetic underpinnings of migraine? What is the role of psychological factors like stress, personality, and psychiatric comorbidities? How do family and environmental factors (e.g., family dysfunction, adverse childhood experiences) influence migraine? | This article is a narrative literature review that synthesizes findings from neuroimaging, genetic, and psychological research to provide a comprehensive overview of pediatric migraine. | The total number of studies is not specified. The review draws upon various study types, including fMRI studies, genome-wide association studies, twin studies, and clinical psychological research. |
| **Pizer et al. (2025)** | Neuropsychological Functioning in Pediatric Primary Headache Disorders: A Meta-Analysis | To quantitatively evaluate whether neuropsychological functioning is impaired among children and adolescents with primary headache disorders (PHD) when compared to peers without headaches. | Is neuropsychological test performance reduced in children with PHDs compared to healthy controls, both overall and within specific cognitive domains? The authors hypothesized that the largest deficits would be in executive function, attention, processing speed, and learning/memory. | The meta-analysis followed PRISMA guidelines. A literature search was conducted in PubMed, Embase, ProQuest Health & Medical, ProQuest Psychology Database, and PsycINFO up to February 2024. Two authors independently screened studies for inclusion and extracted data. Data were pooled using random-effects models, with Hedges' g as the effect size measure. Heterogeneity and publication bias were formally assessed. | 16 observational studies were included in the quantitative synthesis. These studies compared a pediatric sample (ages <20) with a primary headache disorder to a healthy control sample using validated clinical neuropsychological measures. |
| **Tarantino et al. (2022)** | Interictal Cognitive Performance in Children and Adolescents With Primary Headache: A Narrative Review | Interictal Cognitive Performance in Children and Adolescents With Primary Headache: A Narrative Review. | The review aimed to determine if neuropsychological difficulties, particularly those involving memory and attention, are common in pediatric migraine and if cognitive impairment depends on the frequency of attacks and the duration of the disease. | Narrative Review. A literature search was conducted on MEDLINE and Web of Science for studies published up to December 2021, using various keywords related to pediatric headache and cognitive functions. | 25 articles were included, comprising prospective, observational, and retrospective studies, as well as multicentric studies and clinical trials. |

**Table S2 (part 1 of 2).** Reviews and/or meta-analyses that met the inclusion criteria. *Note: Table S2 is presented in two parts; see part 2 for continuation.*

| | | | | | |
|---|---|---|---|---|---|
| **Yum & Chu (2025)** | Unraveling the connections between migraine and psychiatric comorbidities: A narrative review | To examine the relationship between migraine and various psychiatric comorbidities (specifically mood disorders, autism spectrum disorder [ASD], and ADHD), with a focus on shared mechanisms, diagnostic challenges, and integrated treatment approaches, particularly in pediatric populations. | The review explores: (1) The evidence for the comorbidity between migraine and psychiatric disorders like depression, anxiety, bipolar disorder, ASD, and ADHD; (2) The shared pathophysiological mechanisms that might explain these links; (3) The unique challenges in diagnosing migraine in patients with these comorbidities; and (4) How treatments for these conditions interact. | Narrative Review. The authors synthesized findings from a wide range of existing literature, including meta-analyses, genetic studies, and clinical trials to build a comprehensive overview of the topic. | As a narrative review, the article does not state a specific number of included studies. |
| **Orr (2024)** | Headache in Children and Adolescents | To reviews the assessment of children and adolescents presenting with headache, provides an overview of primary headache disorders, and reviews evidence-based management of headache in this age group. | The review addresses several key clinical questions, including: How should clinicians evaluate a child with headache? What are the diagnostic criteria and features of various primary headaches in this age group? What are the evidence-based acute and preventive treatment strategies? What is the role of comorbidities and lifestyle factors? | Narrative Review. The author synthesized information from the existing medical literature, including epidemiological data, diagnostic criteria from the International Classification of Headache Disorders (ICHD-3), and evidence-based guidelines, to provide a clinical overview. | As a narrative review, a specific number of included studies is not provided. |

**Table S2 (part 2 of 2).** Reviews and/or meta-analyses that met the inclusion criteria (continued from part 1).

| Authors and Year | Main Synthesized Results / Emerging Themes | General Conclusions of the Review | Identified Limitations (of the literature) | Future Research Directions |
|---|---|---|---|---|
| **Ayta et al. (2016)** | Impact on QoL: Headaches negatively impact QoL, often more than other chronic illnesses. Psychological Impact: There is a strong link to depression, anxiety, and stress. Academic Impact: Headaches are a major cause of school absenteeism and poor performance. Social Impact: Peer relationships and social activities are often impaired. | Early identification and treatment are crucial not just for pain relief but also for a child's overall academic, social, and psychological development. | The article highlights a general lack of academic knowledge, especially concerning secondary headaches and their impact on QoL. | More studies are needed to inform families and physicians. Research should focus more on secondary headaches and their consequences. |
| **Basile et al. (2024)** | There is a strong and bidirectional relationship between headaches and sleep disorders in children. A wide range of sleep problems (insomnia, difficulty falling asleep, night waking, daytime sleepiness) are linked to headaches. Worse headache characteristics (higher frequency or intensity) are correlated with more severe sleep problems, and vice versa. This connection is likely due to shared brain structures and neurochemical pathways. | The comorbidity is frequent and well-documented. Clinicians should assess for sleep problems in children with headaches (and assess for headaches in children with sleep problems) to provide effective treatment and improve quality of life. | The primary limitation noted was methodological: none of the reviewed studies used the official International Classification of Sleep Disorders criteria to diagnose sleep disorders, relying instead on questionnaires. | The conclusion emphasizes the need for clinicians to apply the current evidence in daily practice by using validated assessments for both headaches and sleep disorders to ensure early detection and better treatment strategies. |
| **Dosi et al. (2015)** | Bidirectional Relationship: The link between sleep and headaches is mutual; sleep disturbance can trigger headaches, while headaches disrupt sleep. Conversely, sleep can also terminate a migraine attack. Early Life Origins: The connection may begin in infancy, with conditions like infantile colic possibly representing an early form of migraine. Common Sleep Disorders: Headaches in children are frequently associated with parasomnias, sleep-disordered breathing, Restless Legs Syndrome (RLS), and Periodic Limb Movements during Sleep (PLMS). | A strong, genetically-based comorbidity exists between sleep disorders and headaches in the pediatric population. The clinical assessment of children with headaches must include a thorough evaluation of sleep. An integrated management approach that addresses both conditions is essential for effective treatment. | The review does not state its own limitations but notes that the relationship between sleep and headache is complex and that some aspects require further clarification through research. | The article implies a need for continued research into the shared neurobiological substrates of sleep and headache and underscores the clinical importance of an integrated assessment and management strategy. |
| **Dyb et al. (2015)** | Both migraine and TTH are linked to internalizing symptoms (anxiety, depression). The relationship is likely bidirectional, involving shared risk factors (like childhood adversity), headaches causing psychological issues, and psychological issues predicting headaches. The risk of comorbidity increases with headache frequency. | Psychiatric issues significantly worsen the quality of life in young headache patients. Clinical assessments should therefore include psychological and social factors to plan more effective, targeted treatments. | The literature is limited by the use of diverse measurement tools for psychiatric symptoms and a predominance of cross-sectional studies, which makes it difficult to determine causality | The authors suggest a need for more longitudinal studies to clarify causal pathways and for new interventions that treat both the physical and psychological aspects of the conditions. |
| **Gelfand (2015)** | Clinic vs. Population: Children with migraine seen in specialized clinics may have slightly higher anxiety/depression scores, but these scores are rarely in the clinically significant range. In the general population, scores are generally not elevated. | The majority of children and adolescents with migraine do not have a comorbid psychiatric disorder. There does not appear to be a clear, widespread association between pediatric migraine and clinical depression or anxiety. | The primary limitation identified is the use of suboptimal measurement tools (questionnaires) that are confounded by migraine symptoms. Another issue is the selection bias in clinic-based studies, which may not represent the general population of children with migraine | Future studies should use psychiatric assessment tools that do not include migrainous symptoms to get a more accurate picture. The review also suggests that the association between migraine with aura and anxiety warrants further investigation. |

**Table S2 (part 2 of 2).** Reviews and/or meta-analyses that met the inclusion criteria (continued from part 1).

| | | | | |
|---|---|---|---|---|
| **Genizi et al. (2017)** | LD are widespread in children with primary headaches, particularly migraine. They may present with specific cognitive deficits in memory, attention, and processing speed. The link is complex and could be both psychosocial (e.g., fear of failure) and neurobiological. | Learning disabilities have a high prevalence in children with primary headaches. The connection could stem from a common brain pathophysiology or be a consequence of poor quality of life. | The review highlights the scarcity of high-quality studies (both clinical and population-based) on this specific topic | There is an urgent need for further population-based and clinical studies to better understand the complex relationship between pediatric headaches and learning disabilities. |
| **Eidlitz Markus & Toldo (2018)** | The literature suggests an association between obesity and pediatric headaches, though the specific link to migraine is less clear. The connection is likely multifactorial, involving shared neurobiological mechanisms (e.g., hypothalamic dysfunction, altered serotonin levels) and pro-inflammatory states. Some studies indicate that weight loss interventions can reduce headache frequency. | Many open questions remain regarding the modifiable nature of the obesity-migraine relationship and its implications in clinical practice | The review notes that existing research on the association specifically between migraine and obesity in children is limited and has yielded some contradictory results. Furthermore, no wide-ranging meta-analyses have been conducted in this specific population. | Further studies are needed to clarify the modifiable nature of the obesity-migraine relationship and its implications for clinical practice. |
| **Falla et al. (2022)** | Children with migraine have significantly higher levels of anxiety symptoms (large effect) and depressive symptoms (moderate effect) compared to healthy controls. The odds of having a clinical diagnosis of an anxiety disorder or depressive disorder were found to be approximately double in youth with migraine. The results did not significantly change when comparing studies from clinical samples versus those from the general population. | Children and adolescents with migraine are at greater risk for anxiety and depressive symptoms and disorders compared to their healthy peers. Consequently, routine screening for these disorders in clinical practice could be beneficial. | Many included studies did not adjust their analyses for potential confounding factors, and over a quarter of the studies were of low quality. Additionally, no studies were found that investigated the relationship between migraine and trauma- or stressor-related disorders, representing a significant gap in the literature. | Future research should clarify whether the presence of anxiety and depression negatively influences migraine outcomes or increases the risk of developing it. Furthermore, the potential association between migraine and trauma- and stressor-related disorders needs to be investigated. |
| **Langdon et al. (2020)** | Academic performance in youth with migraine is influenced by a network of factors. Migraine shows a positive correlation with ADHD (especially if chronic) and learning disabilities. Issues like sleep disorders, anxiety, and depression are common and worsen the clinical picture. Furthermore, psychosocial stressors such as bullying and the pressure related to perfectionism also play a role. | Children with migraine are at risk for academic difficulties, due to both the direct impact of pain and the frequent associated medical and psychiatric comorbidities. | The literature on the link between pediatric migraine, ADHD, and learning disabilities is still limited. There is also a lack of data on the impact of screen time. | Further studies are needed to clarify the shared biological and environmental mechanisms between migraine, psychiatric comorbidities, and academic difficulties. School-based support initiatives and specialized clinics could be beneficial. |
| **O'Brien & Slater (2016)** | High but Variable Prevalence: The prevalence of psychiatric disorders in youth with chronic headaches is high, though reported rates vary widely across studies. Some studies find that up to 65.5% of pediatric migraine patients have at least one psychiatric disorder. Anxiety and Depression: Rates of anxiety and depression are generally higher in children with migraine and tension-type headache (TTH) compared to the general pediatric population. Behavioral Disorders: Conditions like ADHD and Oppositional Defiant Disorder (ODD) also appear to be more common in the pediatric headache population. Barriers to Diagnosis: Accurately determining prevalence is difficult due to inconsistencies in the | Psychological conditions are common in children with chronic headaches, but the true prevalence remains unclear. Early identification and treatment of these comorbidities are important, as they may lead to improved headache outcomes. | The prevalence of anxiety and depression in pediatric migraine is not well established, with highly variable reported rates. There is a lack of validated screening tools specifically for psychiatric disorders in the pediatric headache population. Some general screening tools may be insensitive to the emotional effects of living with a chronic illness | The review highlights a need for more studies with consistent methodologies to clarify the true prevalence of psychiatric conditions in pediatric headache. It also underscores the importance of using validated, low-cost screening tools for early identification. |

**Table S2 (part 2 of 2).** Reviews and/or meta-analyses that met the inclusion criteria (continued from part 1).

| | | | | |
|---|---|---|---|---|
| | literature, a lack of validated screening tools for this specific group, and differences in patient samples (e.g., specialty clinic vs. community). | | | |
| **Paolino et al. (2015)** | Controversial Link: The review highlights that the scientific literature provides conflicting evidence on the epidemiological association between headache and ADHD in children. Dopaminergic System: A key theme is the potential role of the dopaminergic system. Dysfunction in dopamine pathways is central to ADHD and has also been strongly implicated in migraine pathophysiology, especially in premonitory symptoms. Brain Iron Metabolism: Abnormal brain iron metabolism is proposed as another link. Iron deficiency can impair dopamine synthesis (relevant to ADHD) and has also been associated with altered pain processing networks in the brain relevant to migraine. Sleep Disturbances: Sleep problems are common in both ADHD and headache. The review suggests that dysfunction in shared brain structures that regulate both sleep and pain, like the hypothalamus, could be a key factor connecting the two disorders. | While the epidemiological link between headache and ADHD is not definitively established, there are several plausible shared biological mechanisms. Dysfunction in the dopaminergic system, abnormal brain iron metabolism, and disordered sleep regulation appear to be key overlapping pathways that could explain comorbidity. | The existing literature on the association between headache and ADHD is described as "lacking and contradictory." | Further research, particularly functional neuroimaging studies focusing on the hypothalamus and dopaminergic systems, is needed to better define the complex association between headache, ADHD, and sleep disorders, and to understand their combined impact on child development. |
| **Polese et al. (2022)** | Child Psychological Factors: There is a strong and consistent association between headaches and the presence of both internalizing symptoms (anxiety, depression) and externalizing symptoms (behavioral problems) in children. Adverse Events: Stressful environments and adverse life events, especially bullying and school-related stress, are frequently linked to childhood headaches. Parental and Family Factors: Family conflict and unhappiness were commonly associated with headaches in children. However, factors like parental divorce or living in a single-parent family were generally not found to have a significant association. Sleep Disturbances: The majority of studies that looked at sleep found a positive association between sleep problems and headaches. | Headaches in childhood often co-occur with psychological distress in the child and their family. Therefore, a child presenting with headaches should be seen as a potential "alarm bell" for clinicians to investigate underlying psychological disorders or adverse environmental factors. | The studies included in the review were highly heterogeneous in their methods and assessment tools. The majority of studies did not clearly differentiate between specific headache types (e.g., migraine vs. tension-type headache), often grouping them together. There was a lack of data on how cultural factors might influence the findings. | Further studies are needed to better understand the specific role and directionality of the relationship between early-life adverse events, family factors, and the development of headaches in children. |
| **Salem et al. (2018)** | The meta-analysis revealed a statistically significant positive association between ADHD and migraine (Odds Ratio = 1.32), indicating that individuals with ADHD are more likely to have migraine. In contrast, there was no significant association found between ADHD and tension-type headache (TTH) or between ADHD and "headache" in general (when all types were pooled). The review discusses potential shared | The findings provide compelling evidence for a specific link between ADHD and migraine, but not with other primary headache types like TTH. The biological mechanisms for this association remain unclear and require further study. | The studies included in the review were highly heterogeneous in their methodologies and assessment tools. Most of the available research was cross-sectional, which prevents any conclusion about causality (i.e., whether ADHD leads to migraine or vice versa). The potential confounding effect of stimulant medication for ADHD (which can cause headaches as a side | There is a need for more high-quality, prospective longitudinal studies to clarify the nature and direction of the relationship between ADHD and migraine. |

**Table S2 (part 2 of 2).** Reviews and/or meta-analyses that met the inclusion criteria (continued from part 1).

| | | | | |
|---|---|---|---|---|
| | underlying mechanisms for the ADHD–migraine link, including dysfunction in the dopaminergic system and a possible shared genetic basis. | | effect) was often not addressed in the original studies. | |
| **Shimomura (2022)** | Personality Traits: Children with primary headaches often exhibit internalizing personality characteristics. Alexithymia (difficulty identifying and describing feelings) is a common trait that is thought to worsen both migraine and tension-type headache (TTH). Psychiatric Comorbidities: Anxiety and depression are the most significant psychiatric comorbidities. Headaches are about twice as common in depressed youth, and higher anxiety levels are linked to more frequent and severe migraines. Neurodevelopmental Disorders: There is a strong association between headaches and neurodevelopmental disorders. A meta-analysis confirmed a specific link between migraine and ADHD. Migraine is also common in children with Autism Spectrum Disorder (ASD), which may be related to sensory hyperreactivity. Management: There is little strong evidence for specific medications for headaches in children with comorbid emotional issues, highlighting the importance of non-pharmacological approaches like psychotherapy. | Children with primary headaches frequently have co-occurring emotional problems, including personality traits like alexithymia, psychiatric disorders (anxiety, depression), and neurodevelopmental disorders (ADHD, ASD). Recognizing and addressing these issues through a combination of medical and psychological treatments is crucial for improving headache outcomes. | There is a lack of research on personality traits in pediatric headache patients compared to adults. It is often difficult to determine causality (e.g., whether emotional problems are a cause or a consequence of chronic headaches). There is a scarcity of high-quality evidence for pharmacological treatments in this specific comorbid population. | More prospective studies are needed to clarify the causal relationship between personality and headache development. Research on the effectiveness of combining psychotherapy with medical treatment is also essential. |
| **Guidetti e al. (2019)** | Neurological Factors: Migraine is associated with structural and functional alterations in brain networks involved in pain, emotion, and cognition, such as the Default Mode Network (DMN) and the insula. These changes may contribute to mild cognitive difficulties in memory and verbal skills. Psychological Factors: Stress is a key trigger. Children with migraine often have comorbid internalizing disorders (anxiety, depression) and alexithymia (difficulty identifying/describing emotions). Personality traits like harm avoidance and perfectionism are also common. Environmental and Family Factors: The family environment plays a crucial role. Dysfunctional family dynamics, insecure attachment styles, parental psychiatric illness (especially maternal), and adverse childhood experiences (e.g., abuse, bullying) are all significant risk factors for migraine. | Migraine in childhood is best understood not as a purely organic disease but as a complex biobehavioral or psychosomatic disorder. It arises from an interaction between a child's genetic predisposition and various psychological and environmental factors. | The causal direction of many associations is unclear (e.g., whether migraine causes brain changes or vice-versa). The precise mechanisms linking environmental stress to biological changes in migraine are still under investigation. | The review emphasizes the need for an integrated approach that considers the interplay of biological, psychological, and environmental factors to fully understand and treat the complexity of pediatric migraine. |

**Table S2 (part 2 of 2).** Reviews and/or meta-analyses that met the inclusion criteria (continued from part 1).

| | | | | |
|---|---|---|---|---|
| **Pizer et al. (2025)** | Overall, children with PHD showed significantly worse neuropsychological performance compared to controls (Hedges' g=−0.31). Deficits were observed across several domains, including motor, executive function, learning/memory, language, processing speed, intelligence, and visuospatial skills. Sensitivity analyses revealed that these negative effects were primarily driven by samples with migraine, as the overall effect for tension-type headache (TTH) samples was not significant. | Pediatric samples with migraine demonstrate worse neurocognitive performance, both globally and in specific domains, compared to healthy controls. This suggests that cognitive deficits associated with migraine can manifest well before adulthood. The findings help to clarify the typical neurocognitive profile of pediatric migraine, which can inform diagnosis, treatment planning, and recommendations. | A primary limitation was the small number of included studies, particularly for TTH, which likely resulted in underpowered moderator analyses. Most studies failed to report on key potential moderators such as psychiatric comorbidities, medication use, race/ethnicity, or aura status. All included studies recruited participants from healthcare settings, which limits generalizability to community samples. Finally, the necessary categorization of complex neuropsychological tests into single domains is a simplification that may have influenced the results. | Further research is needed on cognitive performance in pediatric TTH, which may require recruiting community samples. Future studies should investigate the etiology and progression of these cognitive deficits, particularly the large effects seen in the motor domain of pediatric migraine. It is also recommended that future studies consistently report on demographic and clinical variables (e.g., psychiatric conditions, aura status, sample setting) to allow for more robust moderator analyses. |
| **Tarantino et al. (2022)** | Although considered a benign disease, pediatric primary headache may be associated with altered neuropsychological functioning in the interictal phase. While children with migraine generally have normal intelligence, they may exhibit a non-homogeneous cognitive profile characterized by difficulties in verbal skills (especially comprehension), attention, processing speed, and memory (particularly verbal memory). | Pediatric migraine can be associated with altered neuropsychological functioning in the pain-free period, involving language, attentional resources, processing speed, and verbal memory. Given the impact this can have on school performance, a cognitive screening for young patients with primary headache is pivotal. | The reviewed literature shows several limitations: most samples are recruited from specialized headache clinics and are not representative of the general population; samples are often small and heterogeneous; some studies lack a control group; the neuropsychological tools used are extremely variable; and the potential role of psychological symptoms has been scarcely investigated. | Additional neuropsychological research is needed that evaluates larger samples using more homogeneous methods to better understand the cognitive profile of these patients. |
| **Yum & Chu (2025)** | There is a strong, often bidirectional, link between migraine and psychiatric disorders, especially depression and anxiety. Shared mechanisms include genetic predisposition, neurotransmitter imbalances (e.g., serotonin), hormonal influences, and environmental factors. Diagnosing migraine in children with ASD and ADHD is particularly difficult due to communication barriers and overlapping symptoms like sensory hypersensitivity. Treatments for psychiatric conditions and migraine can interact; for example, some migraine prophylactics may worsen depression, while some antidepressants are effective for migraine prevention. | The relationship between migraine and psychiatric comorbidities is complex and requires an integrated clinical approach for effective management. Evaluating and treating psychiatric conditions is a crucial part of comprehensive migraine care, especially as the presence of both tends to result in a more severe clinical impact. | The review notes that reports on the comorbidity of migraine with ASD and ADHD are still scarce. While the link between migraine and depression is strong in children, the bidirectional relationship has not yet been confirmed through longitudinal studies, as it has in adults. | The review highlights the need for integrated clinical approaches, implying that future research should focus on developing and testing treatment strategies that simultaneously address both migraine and its psychiatric comorbidities to improve overall health outcomes. |

**Table S2 (part 2 of 2).** Reviews and/or meta-analyses that met the inclusion criteria (continued from part 1).

| | | | | |
|---|---|---|---|---|
| **Orr (2024)** | Primary headache disorders, particularly migraine, are highly common and disabling in children and adolescents, affecting approximately 10% of this population. Early diagnosis and effective treatment are crucial, as they may improve long-term headache and mental health outcomes. The clinical assessment must differentiate between primary and secondary headaches, with attention paid to red flags. Comorbidities are common, and all children with migraine should be screened for anxiety and depression. Evidence-based treatments include acute therapies (e.g., ibuprofen, triptans) and a range of preventive options, including pharmacologic, psychological, and neuromodulation interventions. | Headache is a significant health problem in children and adolescents that requires a comprehensive approach to diagnosis and management. Treatment decisions should be guided by the available evidence and made through a shared decision-making process with the patient and family. Effective and equitable care can reduce the current burden of the disease and has the potential to mitigate long-term disability into adulthood. | Progress in the field has been impeded by a significant lack of research funding compared to other pediatric diseases. Evidence for many treatments in children is limited, with many therapies being used off-label based on adult data. The evidence supporting lifestyle recommendations (e.g., related to sleep, diet, and exercise) is derived mainly from observational studies rather than robust clinical trials. | New treatments targeting the calcitonin gene-related peptide (CGRP) pathway and noninvasive neuromodulation devices, while primarily studied in adults, have emerging evidence for use in the pediatric population and represent a key area for future research. There is a critical need to improve equitable access to diagnosis and care for all children with headache disorders. |
